# Piperidine CD4-Mimetic Compounds Expose Vulnerable Env Epitopes Sensitizing HIV-1-Infected Cells to ADCC

**DOI:** 10.3390/v15051185

**Published:** 2023-05-17

**Authors:** Shilei Ding, William D. Tolbert, Huile Zhu, Daniel Lee, Lorie Marchitto, Tyler Higgins, Xuchen Zhao, Dung Nguyen, Rebekah Sherburn, Jonathan Richard, Gabrielle Gendron-Lepage, Halima Medjahed, Mohammadjavad Mohammadi, Cameron Abrams, Marzena Pazgier, Amos B. Smith, Andrés Finzi

**Affiliations:** 1 Centre de Recherche du CHUM, Montreal, QC H2X 0A9, Canada; 2 Infectious Disease Division, Department of Medicine, Uniformed Services University of the Health Sciences, Bethesda, MD 20814, USAdung.nguyen.ctr@usuhs.edu (D.N.);; 3 Department of Chemistry, School of Arts and Sciences, University of Pennsylvania, Philadelphia, PA 19104, USA; 4 Département de Microbiologie, Infectiologie et Immunologie, Université de Montréal, Montreal, QC H3T 1J4, Canada; 5 Department of Chemical and Biological Engineering, Drexel University, Philadelphia, PA 19104, USA

**Keywords:** HIV-1, small CD4 mimetic compounds (CD4mc), Phe43 cavity, envelope glycoproteins, neutralization, antibody-dependent cellular cytotoxicity (ADCC)

## Abstract

The ability of the HIV-1 accessory proteins Nef and Vpu to decrease CD4 levels contributes to the protection of infected cells from antibody-dependent cellular cytotoxicity (ADCC) by preventing the exposure of Env vulnerable epitopes. Small-molecule CD4 mimetics (CD4mc) based on the indane and piperidine scaffolds such as (+)-BNM-III-170 and (*S*)-MCG-IV-210 sensitize HIV-1-infected cells to ADCC by exposing CD4-induced (CD4i) epitopes recognized by non-neutralizing antibodies that are abundantly present in plasma from people living with HIV. Here, we characterize a new family of CD4mc, (*S*)-MCG-IV-210 derivatives, based on the piperidine scaffold which engages the gp120 within the Phe43 cavity by targeting the highly conserved Asp^368^ Env residue. We utilized structure-based approaches and developed a series of piperidine analogs with improved activity to inhibit the infection of difficult-to-neutralize tier-2 viruses and sensitize infected cells to ADCC mediated by HIV+ plasma. Moreover, the new analogs formed an H-bond with the α-carboxylic acid group of Asp^368^, opening a new avenue to enlarge the breadth of this family of anti-Env small molecules. Overall, the new structural and biological attributes of these molecules make them good candidates for strategies aimed at the elimination of HIV-1-infected cells.

## 1. Introduction

HIV-1 envelope glycoproteins (Env) mediate virus infection by binding to CD4 on the surface of host cells [1,2,3]. Env consists of a trimer of heterodimers made of a transmembrane (gp41) and surface (gp120) subunit. Upon CD4 binding, the gp120 ensues a series of conformational changes leading to the exposure of the coreceptor binding site (CoRBS) and the gp41 helical heptad repeat (HR1) [4]. These CD4-induced (CD4i) epitopes can be recognized by non-neutralizing antibodies (nnAbs) abundantly present in plasma of people living with HIV (PLWH) [5], some of which are able to mediate antibody-dependent cellular cytotoxicity (ADCC) [6,7].

Small-molecule CD4-mimetic compounds (CD4mc) trigger similar conformational changes as CD4 by engaging the gp120 within the highly conserved region of Env that accommodates the Phe43 of CD4 (referred to as the Phe43 cavity) [8,9,10,11]. Based on the original compounds NBD-556 and NBD-557, discovered by Debnath [12], derivatives such as JP-III-48 or (+)-BNM-III-170 were developed with a better capacity to neutralize HIV-1 virus particles and sensitize HIV-1-infected cells to ADCC [9,10,13,14,15,16,17]. Further screening of small molecules led to the finding of piperidine analog (*S*)-MCG-IV-210, which engages the Phe43 cavity in a similar manner to that of (+)-BNM-III-170 while being in closer proximity to the highly conserved CD4-binding residue Asp^368^ [18].

In this study, analogs of the piperidine (*S*)-MCG-IV-210 were designed and synthesized with the goal to improve their interaction with Asp^368^. The analogs’ development was guided by high-resolution structures of the complexes formed by newly developed CD4mc and a gp120 Env core that was stabilized in the gp120 CD4-bound confirmation [10,18,19,20]. The structures provide insights into the analogs’ interactions within the CD4-binding cavity and how they specifically interact with Asp^368^. The capacity of this new series of compounds to neutralize viral particles and sensitize infected cells to ADCC was measured.

## 2. Results

### 2.1. New Piperidine Analogs

**Figure 1:**
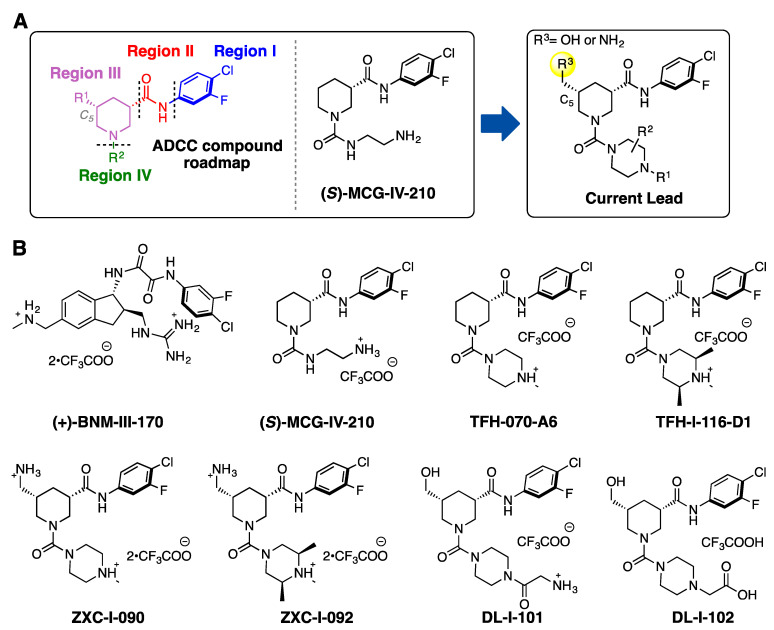
(**A**) A structural roadmap (i.e., Region I-IV) of the lead molecule scaffold. (**B**) Chemical structures of (+)-BNM-III-170, (*S*)-MCG-IV-210, TFH-070-A6, TFH-I-116-D1, ZXC-I-090, ZXC-I-092, DL-I-101, and DL-I-102.

Recently, we reported on the identification and development of a new class of piperidine-based small molecules, namely, (*S*)-MCG-IV-210 and derivatives thereof, exhibiting anti-viral properties that lead to the sensitization of HIV-1-infected cells to ADCC [18,21]. The structure of (*S*)-MCG-IV-210 features a pendant 4-chloro-3-fluoro-arene (Region I) which inserts deeply into the Phe43 cavity, an amide linker (Region II) that extends the molecule out of the Phe43 cavity, and a piperidine ring (Region III) that has been determined to be essential for the anti-viral activity (Figure 1A).

In this study, we present new analogs where the linear urea in Region IV of the molecule skeleton has been replaced with heterocyclic groups such as a methyl-substituted piperazine (TFH-070-A6, Figure 1B) or a functionalized piperazine (TFH-I-116-D1, Figure 1B). To enable the molecule to contact the Asp^368^ carboxylate side chain, we modified the C_5_ position of the piperidine by adding a hydrogen bonding promoter such as (i) primary amines on TFH-070-A6 or TFH-I-116-D1 to make ZXC-I-090 or ZXC-I-092 (Figure 1B) and (ii) hydroxy methyl groups found in DL-I-101 or DL-I-102, with further modifications to the Region IV based on the previously reported (*S*)-MCG-IV-210 scaffold.

### 2.2. The Design and Synthesis of New CD4mc Piperidine Analogs

**Figure 2:**
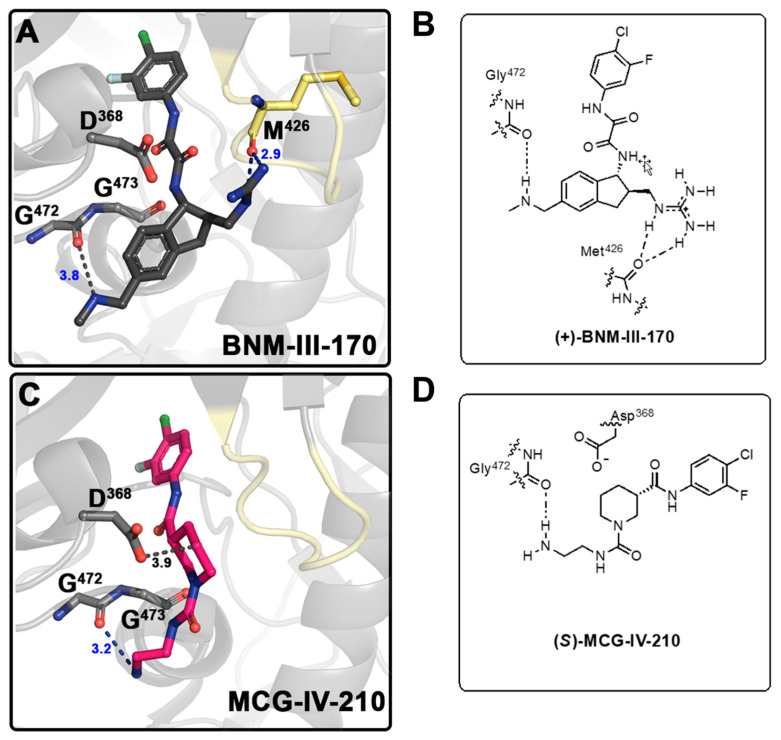
**Development of CD4mc Piperidine Analogs**. Structure of the complex of (+)-BNM-III-170 (PDB code: 6UT1) (**A**) and (S)-MCG-IV-210 (PDB code: 6USW) (**C**) bound to clade A/E gp120_CRF01_ LMHT core [10,18]. H-bonds are shown as blue dashed lines and other contacts to Gly^472^ Met ^426^and Asp^368^ as grey dashed lines. (**B**) Graphical illustration of (+)-BNM-III-170 in contact with Gly^472^ and Met^426^. (**D**) Graphical illustration of (S)-MCG-IV-210 in contact with Gly^472^.

We began the structure-based design of the new CD4mc piperidine analogs by comparing the high-resolution crystal structures of the complexes of LMHT gp120_CRF01_AE_ core_e_ (His 375 was replaced by Thr together with six co-evolving gp120 inner domain residues, pre-shaping the Phe-43 cavity for interaction with CD4mcs) [10,18,19,20] with (*S*)-MCG-IV-210 and indane CD4mc (+)-BNM-III-170 (Figure 2 and Figure S1). While both small molecules CD4mcs contacted Gly^472^, we observed in the gp120 complex of (*S*)-MCG-IV-210 that the linear urea in Region IV of the molecule did not engage the β20-21 loop (Figure 2C). However, we observed that the C_5_ position of the piperidine scaffold was in close proximity to the highly conserved Asp^368^ side chain residue within the Phe43 cavity on the gp120 complex.

To this end, we initiated our study via the derivatizations of various regions of the (*S*)-MCG-IV-210 scaffold with the aim of (i) continuing the structure optimization of Region IV to probe the β20-21 loop within the gp120 complex and (ii) incorporating a polar moiety on the C_5_ of the piperidine ring (Region III) to establish an electrostatic interaction with Asp^368^.

### 2.3. Biological Testing

**Figure 3:**
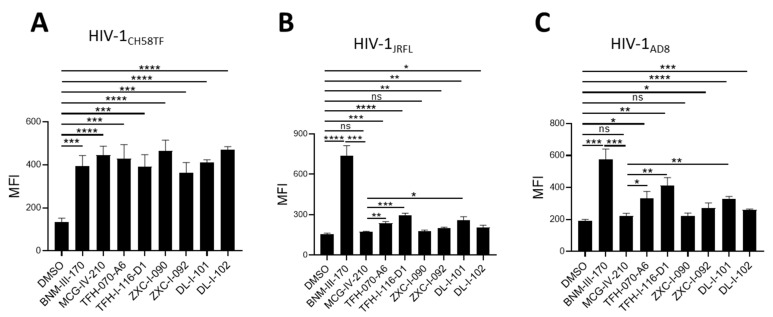
**Anti-CoRBS Ab binding**. Anti-CoRBS Ab—17b binding to (**A**) HIV-1_CH58TF_, (**B**) HIV-1_JRFL_, or (**C**) HIV-1_AD8_-infected primary CD4+ T cells was performed in the presence of 50 µM of the indicated CD4mcs or the same volume of vehicle (DMSO). The median fluorescence intensity (MFI) of 17b binding is reported for the productively infected cell (p24+) population. Data shown are the mean ± SD of at least three independent experiments. Statistical significance was evaluated using an unpaired *t* test (*, *p* < 0.05; **, *p* < 0.01; ***, *p* < 0.001; ****, *p* < 0.0001; ns, not significant).

We first tested the ability of the new piperidine analogs to expose the co-receptor binding site (CoRBS) by measuring the binding of the anti-CoRBS 17b mAb to the infected cells. Briefly, we infected primary CD4+ T cells with HIV-1_CH58TF_, HIV-1_JRFL_, or HIV-1_AD8_. Two days later, the infected cells were co-incubated with 50 µM (+)-BNM-III-170, (*S*)-MCG-IV-210, TFH-070-A6, TFH-I-116-D1, ZXC-I-090, ZXC-I-092, DL-I-101, and DL-I-102 or the same volume of DMSO, and the 17b interaction was measured by flow cytometry after intracellular anti-p24 to identify the infected cell population. As previously reported in [18], (*S*)-MCG-IV-210 exposed the CoRBS of cells infected with HIV-1_CH58_, but not the tier-2 HIV-1_JRFL_ (Figure 3A,B). Defined by the sensitivity to antibody-mediated neutralization (tier-1, 2, and 3), tier-2 viruses (HIV-1_JRFL_ and HIV-1_AD8_) are representative of biologically relevant circulating HIV-1 strains which are moderately neutralized by neutralizing antibodies [22,23,24,25]. Weak neutralizing antibodies, or first generation CD4mc, prevent the infection of viruses from tier-1, but not those from tier-2 or tier-3. Interestingly, the newly developed analogs—TFH-070-A6, TFH-I-116-D1, ZXC-I-092, DL-I-101, and DL-I-102—successfully exposed the CoRBS of HIV-1_JRFL_-infected cells, albeit to a lower level than the potent (+)-BNM-III-170 (Figure 3B). A similar phenotype was observed when using HIV-1_AD8_-infected cells (Figure 3C). This confirms the superior activity of the new analogs over the previous lead for this new class of CD4mc.

### 2.4. Structural Basis of Interaction of (S)-MCG-IV-210 Derivatives with a gp120 Core

**Figure 4:**
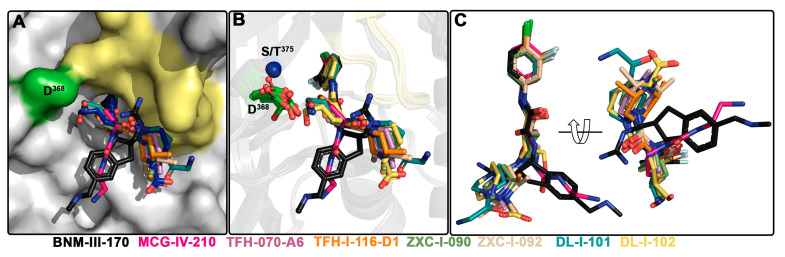
**Crystal structures of (+)-BNM-III-170, TFH-070-A6, TFH-I-116-D-1, TFH-II-151, ZXC-I-090, ZXC-I-092, DL-I-101, and DL-I-102 derivatives in complex with a gp120_CRF01_AE_ LMHS or LMHT core_e_**. (+)-BNM-III-170, (*S*)-MCG-IV-210, and piperidine analogs within the Phe^43^ cavity. (**A**) gp120 is shown as a surface with the β20-21 loop region and D^368^ colored pale yellow and green, respectively. (**B**) gp120 is shown as a ribbon with the β21-21 loop region colored yellow, and D^368^ and Ser/Thr^375^ are shown as green sticks or as a blue sphere. (**C**) Superimposition of the piperidine derivatives onto (+)-BNM-III-170 and (*S*)-MCG-IV-210.

To determine the molecular details of the interaction of the new piperidine analogs with Env, we solved high-resolution crystal structures of TFH-070-A6, TFH-I-116-D1, ZXC-I-090, ZXC-I-092, DL-I-101, and DL-I-102 bound to the LMHT gp120_CRF01_AE_ core_e_ [10,18]. The structures were solved from the high resolution of 2.2 Å (DL-I-101) to the lower resolution of 3.5 Å (TFH-I-116-D1). The data collection and refinement statistics are shown in Table 1. In Figure 4, the piperidine analogs are shown within the CD4 (Phe43) binding cavity with (+)-BNM-III-170 and (*S*)-MCG-IV-210 superimposed to highlight the differences in their binding modes. All analogs utilize the 4-chloro-3-fluoro-arene and amide linker to anchor deeply into the Phe43 cavity with largely identical positions and orientations within the cavity (Figure 4B,C). Major differences, however, are observed for the conformations of the piperidine scaffold with the new compounds binding in close proximity to the gp120 β20-21 loop region while preserving contacts to the highly conserved Asp^368^ side chain.

**Figure 5:**
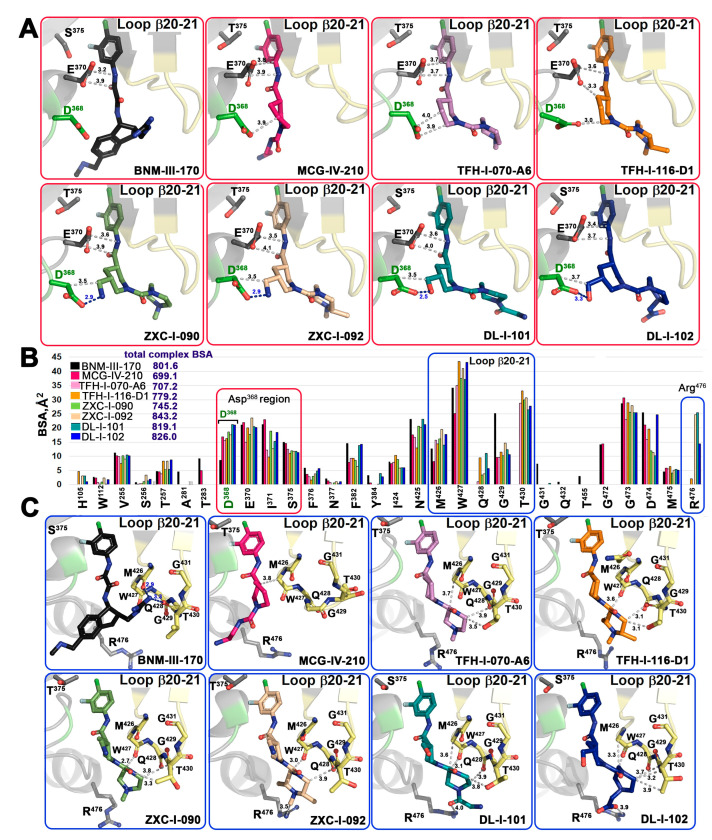
**Molecular details of the interaction of piperidine analogs with the Phe43 cavity of HIV-1 Env**. (**A**,**C**) Details of each compound’s interaction with Asp^368^ and β20-21 loop region, respectively. Crystal structures of each analog in complex with gp120_CRF01_AE_ core_e_ were superimposed based upon gp120 and identical views shown with H-bonds as blue dashed lines. The distances to Asp^368^, Glu^370^, the main chain atoms of the β20-21 loop (residues from Met^426^ to Gly^431^), and the side chains of Thr^430^ and Arg^476^ are shown as grey dashed lines with distances in Å. (**B**) The relative contribution of individual gp120 residues to the binding interface for each compound shown as a buried surface area (BSA) value as calculated by PISA. The buried surface area represents the solvent-accessible surface area of the corresponding residue that is buried upon interface formation. The total BSA for each complex is shown next to the compound label.

Figure 5 shows the details of the interaction of the new compounds within the CD4 binding pocket and the contact network to Asp^368^, Glu^370^ (Figure 5A and Figure S1), and the β20-21 loop region (Figure 5C). A direct comparison of the total buried surface area (BSA) of each compound-gp120 core_e_ complex (Figure 5B) indicates that there is a significant increase in the buried surface for the new compounds as compared to their prototype (*S*)-MCG-IV-210. The highest BSAs are observed for ZXC-I-092 and DL-I-102 with 843.2 Å^2^ and 826.0 Å^2^, respectively (as compared to 699.1 Å^2^ for (*S*)-MCG-IV-210). The analyses of the BSA of individual gp120 residues that contribute to the compound interface (Figure 5B) confirm that the new analogs greatly capitalize upon interactions with Asp^368^, Glu^370^, and the β20-21 gp120 loop. A few of the compounds (ZXC-I-092, DL-I-101, and DL-I-102) also reach Arg^476^. The latter represents a new contact region that has not previously been targeted by this class of CD4mc compounds.

One of the major goals for the structure-based development of this class of piperidine analogs was to make contact to the side chains of Asp^368^ and Glu^370^, among the most conserved Phe43 cavity lining residues among HIV-1 clades. Four of the new analogs achieve this goal (ZXC-I-090, ZXC-I-092, DL-I-101, and DL-I-102) by placing an amine nitrogen or hydroxy methyl oxygen of the piperazine ring at a hydrogen bond distance to the carboxyl group of Asp^368^. ZXC-I-090 and ZXC-I-092 both use their amines to establish 2.9 Å salt bridges with Asp^368^, while DL-I-101 and DL-I-102 use the hydroxyl of their hydroxy methyl to make 2.5 Å and 3.3 Å H-bonds with Asp^368^, respectively. All of the analogs also simultaneously bind close to Glu^370^ with distances to side chain atoms in the range of 3.2–4.1Å.

The ability to rely on binding to the main chain atoms of the β20-21 loop is new for this class of analogs (gp120 residues 426 to 430) (Figure 5C). These include an intensive network of interactions to the main chain oxygens of Met^426^ and Trp^427^, the backbone of Gln^428^, and the side chain of Thr^431^. The BSA for individual residues in this region is significantly higher than the equivalent residues buried at the interfaces of (+)-BNM-III-170 and the prototype (*S*)-MCG-IV-210. Two compounds (DL-I-101 and DL-I-102) add to this network with contacts to Arg^476^ (Figure 5C).

### 2.5. (S)-MCG-IV-210 Derivatives Inhibit Viral Infection

**Figure 6:**
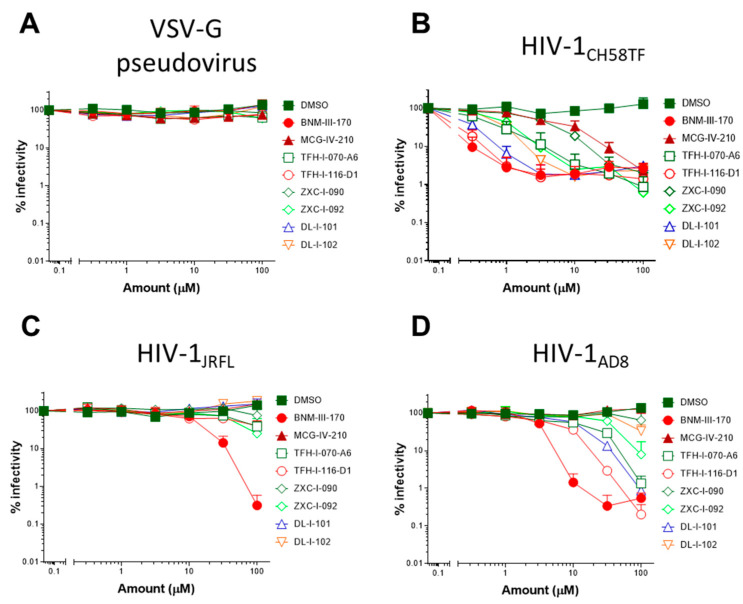

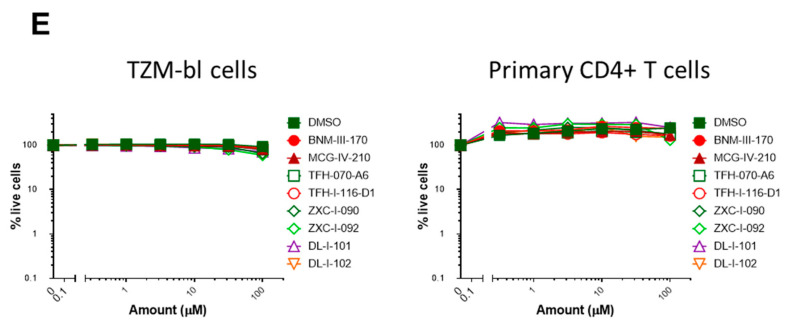
**Small CD4-mimetic neutralization of viral particles**. Neutralization of VSV-G pseudovirus (**A**), HIV-1_CH58TF_ (**B**), HIV-1_JRFL_ (**C**), or HIV-1_AD8_ (**D**) virus was performed with the indicated amounts of different compounds or the same volume of DMSO in TZM-bl cells. Luciferase activity (RLU—relative light units) was measured. Relative infectivity was calculated as the percentage of the value seen in the absence of compounds. Cell viability for TZM-bl ((**E**), left) or primary CD4+ T cells ((**E**), right) with different CD4mcs was measured using CellTiter-Glo One Solution assay for the quantitation of ATP presented in live cells. Data shown are the mean ± SD of at least three independent experiments.

We next evaluated the capacity of (*S*)-MCG-IV-210 derivatives to neutralize viral particles with comparable infectivity of HIV-1_CH58TF_, HIV-1_JRFL_, or HIV-1_AD8_ by using a standard TZM-bl neutralization assay. As a positive control, we used (+)-BNM-III-170 [29]. A VSV-G pseudovirus was used as a negative control. Of note, all tested derivatives were not toxic to the TZM-bl cells (Figure 6E, left) nor the primary CD4+ T cells (Figure 6E, right) at the tested concentrations (maximum 100 µM) and were specific to HIV-1 Env since no effect was observed with the VSV-G pseudoviruses (Figure 6A). As expected, all tested derivatives neutralized HIV-1_CH58TF_ at low micromolar concentrations (Figure 6A), especially TFH-I-116-D1 with an IC_50_ of 0.06548 µM, which were close to that of (+)-BNM-III-170 (0.03358 µM) (Figure 6A, Table 2). While HIV-1_JRFL_ can be inhibited by small CD4-mimetic compounds, only the most potent are able to do so [10] with (+)-BNM-III-170 having an IC_50_ = 13.49 µM. As we reported previously [18], (*S*)-MCG-IV-210 was unable to inhibit viral infection by HIV-1_JRFL_ (Figure 6), but here, we report that several of the derivatives were able to do so. TFH-I-070-A6 presented an IC_50_ of 67.24 µM, TFH-I-116-D1 43.82 µM, and ZXC-I-092 54.22 µM (Figure 6B, Table 2). This is the first time that analogs of (*S*)-MCG-IV-210 were able to neutralize HIV-1_JRFL_. We confirmed the potency of these derivatives using another tier-2 HIV-1 strain (HIV_AD8_, Figure 5C, Table 2). TFH-I-116-D1 neutralized HIV-1_AD8_ with an IC_50_ of 4.804 µM, which was similar to that of (+)-BNM-III-170 (IC_50_ = 3.749 µM). Whether the H-bond formation of TFH-I-116-D1 with Env Asp^368^ contributed to the improved potency of this analog remains to be demonstrated. We believe that this derivative poses a first step in the right direction. Additional structural modifications based on the new structural information provided in Figure 4 and Figure 5 are likely to help achieve this goal.

### 2.6. Sensitization of HIV-1-Infected Cells to ADCC

**Figure 7:**
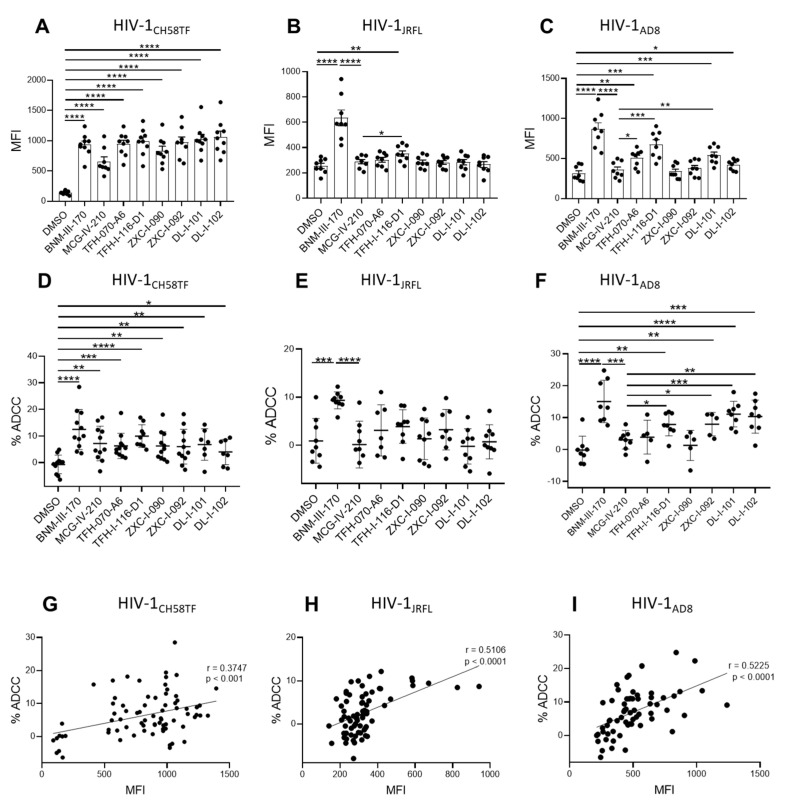
**Piperidine CD4mc analogs sensitize HIV-1-infected cells to ADCC**. Primary CD4 T cells were infected with HIV-1_CH58TF_ (**A**,**D**), HIV-1_JRFL_ (**B**,**E**), or HIV-1_AD8_ (**C**,**F**). HIV+ plasma (1:1000 diluted) was used for staining (**A**–**C**) or ADCC (**D**–**F**) in the presence of 50µM different compounds or with an equivalent volume of vehicle (DMSO). An Alexa Fluor 647-conjugated anti-human IgG secondary Ab was then used for fluorescent labeling (**A**–**C**). Median fluorescence intensity (MFI) in the presence of compounds or that of DMSO was shown; for ADCC, infected cells were used as target cells in a FACS-based ADCC assay that measures the killing of infected p24+ cells_._ The assay determines susceptibility to ADCC mediated by a 1/1000 dilution of plasma from HIV-1-infected individuals in the presence of 50µM different compounds or with an equivalent volume of vehicle (DMSO). (**G**–**I**) The correlation between cell-surface staining with HIV+ plasma and ADCC was calculated using the Pearson r rank correlation. Data shown are the mean ± SD of at least three independent experiments (plasma from more than 5 HIV-1-infected individuals). Statistical significance was evaluated using an unpaired *t* test (*, *p* < 0.05; **, *p* < 0.01; ***, *p* < 0.001; ****, *p* < 0.0001; ns, not significant).

Since CD4mcs are reported to sensitize HIV-1-infected cells to ADCC mediated by HIV+ plasma [17,18,30,31,32,33,34,35], we next evaluated the susceptibility of primary CD4+ T cells infected with HIV-1_CH58TF_, HIV-1_JRFL_, or HIV-1_AD8_ to ADCC mediated by HIV+ plasma in the absence or presence of (*S*)-MCG-IV-210 derivatives, using a FACS-based assay as previously reported [35,36]. As expected, the positive control (+)-BNM-III-170 and *(S)*-MCG-IV-210 enhanced the recognition of HIV-1_CH58TF_ infected cells and their susceptibility to ADCC mediated by HIV+ plasma. This was also the case for the new piperidine CD4mc analogs (TFH-070-A6, TFH-I-116-D1, ZXC-I-090, ZXC-I-092, DL-I-101, DL-I-102) (Figure 7A,D). In agreement with a more CD4mc-resistant phenotype observed with HIV-1_JRFL_-infected cells, the antibodies from PLWH recognized the infected cells only in the presence of TFH-I-116-D1, but not the other derivatives (Figure 7B); this binding was not translated into enhanced ADCC compared to what we observed with *(S)*-MCG-IV-210 (Figure 7E). We observed a more heterogeneous phenotype with HIV-1_AD8_-infected cells, where *(S)*-MCG-IV-210 did not promote HIV+ plasma binding to infected cells, but TFH-070-A6, TFH-I-116-D1, or DL-I-101 did, and translated into ADCC for TFH-I-116-D1 or DL-I-101 (Figure 7C,F).

### 2.7. BLI Competition Assay

**Figure 8:**
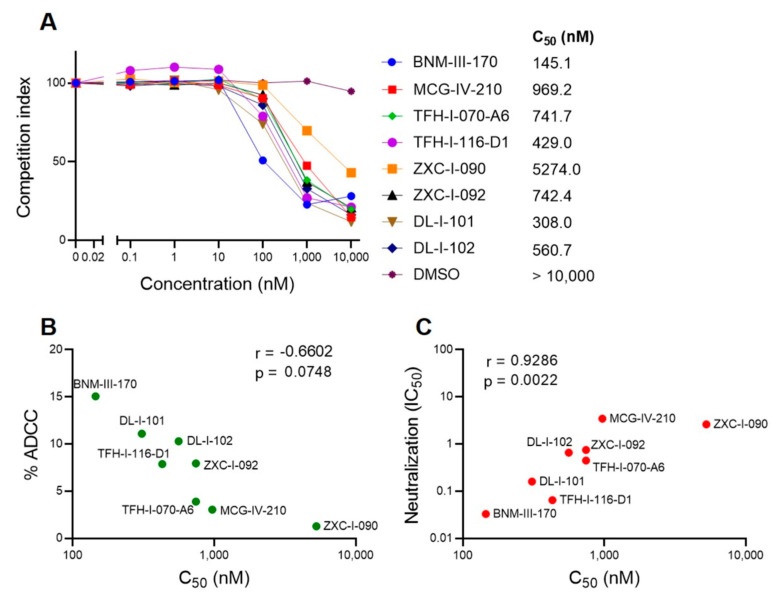
**BLI competition assay**. (**A**) BLI measurements of tested CD4mcs competition with sCD4 binding to LMHT gp120_CRF01_AE_ core_e_ enables determination of the concentration required to outcompete 50% of CD4-Core_e_ binding (C_50_). (**B**) Correlation of ADCC activity against HIV-1_AD8_ and C_50_ from the sCD4 competition assays. (**C**) Correlation of neutralization of HIV_CH58TF_ (IC_50_) and C_50_ from the sCD4 competition assays.

To further evaluate the potency of the different CD4mc, we designed a biolayer interferometry (BLI) competition assay. In this assay, we measured the binding of soluble CD4 (sCD4) to the same gp120 core (LMHT gp120_CRF01_AE_ core_e_) used for co-crystals (Figure 5). The CD4mc concentration required to outcompete 50% of CD4-Core_e_ binding (C_50_) was determined. Figure 8A shows a range of C_50_ among the different CD4mcs. This measure correlates their capacity to sensitize HIV-1-infected cells to ADCC (Figure 8B) and infection inhibition (Figure 8C). These results are significant because BLI used the core_e_ gp120 from a Clade A/E recombinant HIV-1, whereas the ADCC assays used cells infected with Clade B HIV-1_AD8_, and the neutralization assays used another Clade B HIV-1, CH58TF, thus suggesting that strongly conserved features of gp120 determine the vulnerabilities of HIV-1 Envs to CD4mcs.

## 3. Discussion

Small-molecule CD4 mimetics currently attract attention due to their ability to both sensitize HIV-1-infected cells to ADCC [17] and sensitize viral particles to neutralization by otherwise ineffective antibodies [37]. In vivo studies demonstrated the protection of rhesus macaques from high-dose heterologous transmitted/founder simian–human immunodeficiency virus (SHIV) through the combination of CD4mc (+)-BNM-III-170 and non-neutralizing antibodies elicited by a monomeric gp120 antigen [13]. Moreover, studies in humanized mice supporting NK cell function recently showed that CD4mc, in combination with non-neutralizing antibodies, decrease viral replication [33,34] and decrease the level of integrated total DNA in an Fc-dependent manner [34]. To identify additional molecules able to “open-up” Env and expose vulnerable epitopes, we optimized an HTP screening assay using the native trimeric Env to discover a new family of CD4mc able to expose the CoRBS [18]. Further optimization led to (*S*)-MCG-IV-210, a piperidine CD4mc in close proximity with the highly conserved gp120 Asp^368^ residue that sensitized infected cells to ADCC. Stemming from (*S*)-MCG-IV-210, we explored two structural sites, the C_5_ piperidine ring on the linker and the linear urea, which are important for the binding in the Phe43 cavity and in close proximity to Asp^368^ of the envelope glycoprotein. Screening hundreds of analogs for their ability to expose (a) the CoRBS, to enable the recognition of infected cells by HIV+ plasma and (b) inhibit viral infection, followed by structure-based design and evolution, led to six piperidine CD4mc analogs, reported here, with similar or improved potency compared to (*S*)-MCG-IV-210. The structural analysis of this set of analogs, along with that of (+)-BNM-III-170, seems to show that clusters of H-bonds and van der Waals interactions with the β20-21 are correlated with high potencies, as evidenced by TFH-I-116-D1. This is possibly due to a requirement to stabilize the β20-21 in the CD4-bound-state conformation to promote the binding of CoRBS antibodies. Two analogs, DL-I-101 and DL-I-102, donated an H-bond to the α-carboxylic acid group of Asp^368^ and presented improved neutralization and ADCC activities compared to (*S*)-MCG-IV-210, but these compounds displayed less interaction with β20-21 relative to TFH-I-116-D1 and were not superior to it in terms of potency or breadth. This provides a clear direction for further evolution of the piperidine scaffold toward future analogs that simultaneously interact with the highly conserved Asp^368^ and the β20-21 loop, with the goal to improve breadth and potency.

In summary, here, we report on the continuing development of piperidine CD4mc. Several new analogs improved the potency to neutralize difficult-to-neutralized HIV-1 strains (JRFL and AD8) and sensitize HIV-1-infected cells to ADCC. Our results grant further development of piperidine CD4mc and explore tighter interactions with gp120 Asp^368^ and the β20-21 loop with the goal to improve potency and breadth.

## 4. Materials and Methods

### 4.1. Ethics Statement

Written informed consent was obtained from all study participants, and research adhered to the ethical guidelines of CRCHUM and was reviewed and approved on 21 October 2021 by the CRCHUM Institutional Review Board (ethics committee, approval number CE 16.164—CA). Research adhered to the standards indicated by the Declaration of Helsinki. All participants were adults and provided informed written consent prior to enrolment in accordance with Institutional Review Board approval.

### 4.2. Cell Lines and Isolation of Primary Cells

HEK293T human embryonic kidney cells and TZM-bl cells obtained from ATCC were grown as previously described [7,17]. Primary human PBMCs and CD4+ T cells were isolated, activated, and cultured as previously described [7,17]. Briefly, PBMC were obtained by leukapheresis. CD4+ T lymphocytes were then purified from resting PBMCs via negative selection using immunomagnetic beads per the manufacturer’s instructions (StemCell Technologies, Vancouver, BC, Canada). CD4+ T lymphocytes were activated with phytohemagglutinin-L (10 µg/mL) for 48 h and then maintained in RPMI 1640 complete medium supplemented with rIL-2 (100 U/mL).

### 4.3. Chemical Synthesis: General Considerations

Reactions performed under anhydrous conditions were conducted in oven-dried glassware under an inert atmosphere of argon, unless otherwise stated. Commercial sources of chloroform (ethanol-stabilized), methylene chloride (ethanol-stabilized), toluene, tetrahydrofuran, and diethyl ether were dried over CaH_2_, distilled under reduced pressure, and stored over 4Å molecular sieves under an argon atmosphere. All reagents were purchased from commercial sources and used as received. Reaction mixtures were magnetically stirred under an argon atmosphere, unless otherwise noted; reactions were monitored by either thin-layer chromatography (TLC) with 250 μm SiliaPlate^®^ precoated TLC plates or Waters^®^ ACQUITY analytical ultraperformance liquid chromatography (UPLC) system. Yields refer to chromatographically isolated and spectroscopically pure compounds. Optical rotations were measured on a Jasco P-2000 polarimeter. Proton (^1^H) and carbon (^13^C) nuclear magnetic resonance (NMR) spectra were collected on Bruker Avance III 500 (500 MHz) spectrometer. Chemical shifts (δ) are reported in parts per million (ppm) relative to chloroform-d_3_ (δ 7.26), dimethyl sulfoxide-d_6_ (δ 2.50), acetone-d_6_ (δ 2.05), or methanol-d_4_ (δ 3.31) for ^1^H NMR. Chemical shifts (δ) are reported in parts per million (ppm) relative to chloroform-d_3_ (δ 77.16), dimethyl sulfoxide-d_6_ (δ 39.5), acetone-d_6_ (δ 29.8), or methanol-d_4_ (δ 49.0) for ^13^C NMR. High-resolution mass spectrometry (HRMS) was carried out at the University of Pennsylvania Mass Spectroscopy Service Center on either a (i) Waters LCT Premier XE liquid chromatography-mass spectrometry (LC-MS) system or a (ii) Waters GC-TOF Premier system. Preparative-scale UPLC was performed with a Gilson^®^ SPE Purification system equipped with a Sunfire C_18_ OBD column (10 μm packing material, 30 by 150 mm column dimensions), a 215 liquid handler, a 333 binary gradient module, a 156 UV-visible (UV-Vis) dual-wavelength (254 and 365 nm) detector, and Trilution^®^ 3.0 software. Purification solvent systems comprised H_2_O (HPLC-grade) and acetonitrile (HPLC-grade) containing 0.1% trifluoroacetic acid. Supercritical fluid chromatography (SFC) analyses were performed with a Jasco system equipped with a PU-280-CO_2_ plus CO_2_ delivery system, a CO-2060 plus intelligent column thermostat/selector, an HC-2068-01 heater controller, a BP-2080 plus automatic back pressure regulator, an MD-2018 plus photodiode array detector (200 to 648 nm), and PU 2080 plus intelligent HPLC pumps. The purity of new compounds was evaluated via NMR and UPLC-MS (>95%). Chemical synthesis is detailed in the supplemental material.

### 4.4. Viral Production and Infection of Primary CD4+ T Cells

HIV-1 viruses were produced and titrated as previously described [6,18]. Briefly, plasmids expressing the following full-length infectious molecular clones (IMCs) of HIV-1_CH58TF_, HIV-1_JRFL_, or HIV-1_AD8_ were transfected in 293T cells via standard calcium phosphate transfection. Two days after transfection, cell supernatants were harvested, clarified by low-speed centrifugation (5 min at 1500 rpm), and concentrated by ultracentrifugation for 1 h at 4 °C at 143,260× *g* over a 20% sucrose cushion. Pellets were harvested in fresh RPMI, and aliquots were stored at −80 °C until use. Viruses were then used to infect activated primary CD4+ T cells from healthy HIV-1 negative donors via spin infection at 800× *g* for 1 h in 96-well plates at 25 °C; 48h later, ~15% of cells were infected as measured by intracellular p24 staining.

### 4.5. Viral Neutralization

The viral infection assay was performed as previously described [38]. Briefly, TZM-bl target cells were seeded at a density of 1 × 10^4^ cells/well in 96-well luminometer-compatible tissue culture plates (Perkin Elmer, Woodbridge, ON, Canada) 24 h before infection. HIV-1_CH58TF_, HIV-1_JRFL_, or HIV-1_AD8_ viruses with comparable infectivity (~1 million of RLU when infecting TZM-bl cells with 100 μL virus) in a final volume of 100 μL were incubated with indicated amount of different compounds or the same volume of DMSO for one hour at 37 °C, then the mixture was added to the target cells followed by incubation for 48 h at 37 °C; the medium was then removed from each well, and the cells were lysed by the addition of 30 μL of passive lysis buffer (Promega, Madison, WI, USA) and three freeze–thaw cycles. An LB 941 TriStar luminometer (Berthold Technologies, Bad Wildbad, Germany) was used to measure the luciferase activity of each well after the addition of 100 μL of luciferin buffer (15 mM MgSO_4_, 15 mM KPO_4_ (pH 7.8), 1 mM ATP, and 1 mM dithiothreitol) and 50 μL of 1 mM D-luciferin potassium salt (Prolume, Randolph, VT, USA).

### 4.6. Antibodies and Plasma

The anti-CoRBS 17b mAb was used alone or in combination with different compounds for cell-surface staining. Plasma from different HIV-infected donors were collected, heat-inactivated, and conserved as previously described [7,17]. Alexa Fluor 647 conjugated Goat anti-human antibodies (Invitrogen, Waltham, MA, USA) were used as secondary Abs.

### 4.7. Flow Cytometry Analysis of Cell-Surface Staining

Cell-surface staining was performed as previously described [15,16,17]. Primary CD4+ T cells were isolated from healthy donors and infected with HIV-1_CH58TF_, HIV-1_JRFL_, or HIV-1_AD8_. Binding of HIV-1-infected cells via plasma (1:1000 dilution) or 17b mAb (5 μg/mL) in the presence or absence of 50 μM compounds was performed 48 h after infection. Cells were then incubated at 37 °C for 1 h followed by adding anti-human Alexa Fluor 647 (Invitrogen, Waltham, MA, USA) secondary Abs for 20 min. Cells were then stained intracellularly for HIV-1 p24, using the Cytofix/Cytoperm Fixation/ Permeabilization Kit (BD Biosciences, Mississauga, ON, Canada) and the fluorescent anti-p24 mAb (PE-conjugated anti-p24, clone KC57; Beckman Coulter/Immunotech). The percentage of infected cells (p24+ cells) was determined by gating the live cell population on the basis of the AquaVivid viability dye (Thermo Fisher Scientific, Waltham, MA, USA) staining. Samples were analyzed on an LSRII cytometer (BD Biosciences, Bad Wildbad, Germany), and data analysis was performed using FlowJo vX.0.7 (Tree Star, Ashland, OR, USA).

### 4.8. Cell Viability Test

To measure the potential cytotoxicity of the different CD4mcs on TZM-bl or primary CD4+ T cells, a cell viability assay using CellTiter-Glo^®^ One Solution Assay (Promega, Madison, WI, USA) was performed. Briefly, TZM-bl or primary CD4+ T cells were seeded at a density of 1 × 10^4^ cells/well in 96-well luminometer-compatible tissue culture plates (Perkin Elmer, Woodbridge, ON, Canada). After 24 h, indicated concentrations of CD4mcs up to concentrations of 100 μM were added to the cells followed by incubation for 48 h at 37 °C; the same volume of its vehicle, DMSO, was added as control. Then, a volume of CellTiter-Glo^®^ One Solution equal to the volume of cell culture medium present in each well was added, followed by 2 min mixing on shaker and 10 min incubation at room temperature. An LB941 TriStar luminometer (Berthold Technologies, Bad Wildbad, Germany) was used to measure the luciferase activity of each well.

### 4.9. ADCC FACS-Based Assay

Measurement of ADCC using the FACS-based assay was performed at 48 h post-infection as previously described [7,17,35,39]. Briefly, HIV-1_CH58TF_, HIV-1_JRFL_, or HIV-1_AD8_ infected primary CD4+ T cells were stained with viability AquaVivid (Thermo Fisher Scientific, Waltham, MA, USA) and cellular marker cell proliferation dye eFluor670 (eBioscience, San Diego, CA, USA) and used as target cells. Autologous PBMC effectors cells, stained with another cellular marker (cell proliferation dye eFluor450; eBioscience, San Diego, CA, USA), were added at an effector/target ratio of 10:1 in 96-well V-bottom plates (Corning, Corning, NY, USA). Then, the mixed cells were incubated with HIV+ plasma (1:1000), in the presence of 50 µM of compounds or with equivalent volume of vehicle (DMSO). The plates were subsequently centrifuged for 1 min at 300 g, and incubated at 37 °C, 5% CO_2_ for 4 to 6 h before being fixed in a 2% PBS-formaldehyde solution. Samples were analyzed on an LSRII cytometer (BD Biosciences, Bad Wildbad, Germany). Data analysis was performed using FlowJo vX.0.7 (Tree Star, Ashland, OR, USA). The percentage of ADCC was calculated with the following formula: (% of p24+ cells in Targets plus Effectors) − (% of p24+ cells in Targets plus Effectors plus plasma)/(% of p24+ cells in Targets) by gating on infected lived target cells.

### 4.10. Statistical Analysis

Statistics were analyzed using GraphPad Prism version 7.0a (GraphPad, San Diego, CA, USA). Every data set was tested for statistical normality and this information was used to apply the appropriate (parametric or nonparametric) statistical test. *p* values < 0.05 were considered significant; significance values are indicated as * *p* < 0.05, ** *p* < 0.01, *** *p* < 0.001, **** *p* < 0.0001.

### 4.11. CRF01_AE Core e Expression and Purification

Clade AE LM/HT and LM/HS 93TH057gp120_core e_ protein was produced by transfection into GnTI-293F cells. Cells were grown in suspension for 7 days at 37 °C and 90% humidity. The cells were pelleted by centrifugation and the medium was filtered through a 0.2-micron filter. Protein was purified on a 17b affinity column (17b IgG covalently linked to protein A agarose) equilibrated with phosphate-buffered saline (PBS) pH 7.2. The column was washed with PBS and gp120 eluted with 0.1 M glycine pH 3.0. Eluted fractions were immediately diluted 10:1 with 1 M tris(hydroxymethyl)aminomethane-HCl (Tris-HCl) pH 8.5. Eluted protein was concentrated to approximately 10 mg/mL, and the buffer was then exchanged to 50 mM sodium acetate pH 6.0 and 350 mM sodium chloride. EndoH_f_ (New England Biolabs, Ipswich, MA, USA) was added, and the sample was equilibrated overnight at 37 °C to deglycosylate the protein. Deglycosylated protein was then passed over an amylose column equilibrated in 25 mM Tris-HCl pH 7.2 and 200 mM sodium chloride to remove EndoH_f_ (maltose-binding protein tagged EndoH). The protein was concentrated, and the sample was loaded onto a Superdex 200 gel filtration column (Cytiva, Marlborough, MA, USA) equilibrated in 10 mM Tris-HCl pH 7.2 and 100 mM ammonium acetate. Fractions corresponding to the deglycosylated gp120 size were concentrated to approximately 10 mg/mL for use in crystallization trials.

### 4.12. Biolayer Interferometry (BLI) Competition Assay

Biolayer interferometry (BLI) competition assay was performed using an Octet RED96e system (ForteBio, Fremont, CA, USA) at 25 °C with shaking at 1000 RPM. Amine Reactive Second Generation (AR2G) biosensors were hydrated in water, then activated for 300 s with a solution of 5 mM sulfo-NHS and 10 mM EDC (ForteBio, Fremont, CA, USA) prior to amine coupling. Soluble CD4 was loaded into AR2G biosensor at 12.5 µg/mL at 25 °C in 10 mM acetate solution pH 5 (ForteBio, Fremont, CA, USA) for 600 s then quenched into 1 M ethanolamine solution pH 8.5 (ForteBio, Fremont, CA, USA) for 300 s. Loaded biosensors were placed in 10X kinetics buffer (ForteBio, Fremont, CA, USA) for 120 s for baseline equilibration. Association of gp120_core_ LMHT 100nM (in 10X kinetics buffer) to the soluble CD4 was carried out for 180 s in the presence of various concentrations of CD4mc (10 µM to 0.1nM) or equivalent volumes of DMSO prior to dissociation for 300 s. The data were baseline subtracted prior to fitting, performed using a 1:1 binding model and the ForteBio data analysis software (Octet Analysis Studio 13.0). Calculation of response was computed on all data and percentage was obtained by the following calculation: gp120_core_ LMHT + CD4mc/gp120_core_ LMHT × 100.

### 4.13. Crystallization of gp120 Cores Complex with CD4mc

Crystals were grown with the hanging drop method from 10% polyethylene glycol (PEG) 3350, 5% PEG 400, and 0.1 M 4-(2-hydroxyethyl)-2-piperazineethanesulfonic acid (HEPES) pH 7.5. Crystals usually appeared within 1 to 2 weeks when incubated at 21 °C. CD4 mimetic compounds were added by soaking crystals in 1 mM of the compound. Crystals were then frozen for data collection. Prior to freezing, crystals were briefly soaked in the crystallization condition and compound supplemented with 20% of 2-Methyl-2,4-pentanediol (MPD) as a cryoprotectant.

### 4.14. Data Collection, Structure Solution and Refinement

Diffraction data were collected at the Stanford Synchrotron Radiation Light Source (SSRL) beamlines 9-2 and 12-2 on a Dectris Pilatus 6M area detector. All data were processed and reduced with HKL3000 [40] or MOSFLM and SCALA from the CCP4 suite [41]. Structures were solved via molecular replacement with PHASER from the CCP4 suite [41] based upon the coordinates from PDB ID 6ONF. Refinement was carried out with Refmac5 [41] and/or Phenix [42] and model building was performed with COOT [41]. Data collection and refinement statistics are shown in Table 1. Ramachandran statistics were calculated with MolProbity and illustrations were prepared with Pymol Molecular graphics (http://pymol.org).

## Figures and Tables

**Table 1:** Data collection and refinement statistics.

	LM/HT gp120_CRF01_AE_ Core_e_—TFH-I-070-A6	LM/HT gp120_CRF01_AE_ Core_e_—TFH-I-116-D1	LM/HT gp120_CRF01_AE_ Core_e_—ZXC-I-090	LM/HT gp120_CRF01_AE_ Core_e_—ZXC-I-092	LM/HS gp120_CRF01_AE_ Core_e_—DL-I-101	LM/HS gp120_CRF01_AE_ Core_e_—DL-I-102
**Data collection**						
Wavelength, Ǻ	0.979	0.979	0.979	0.979	0.979	0.979
Space group	P2_1_2_1_2_1_	P2_1_2_1_2_1_	P2_1_2_1_2_1_	P2_1_2_1_2_1_	P2_1_2_1_2_1_	P2_1_2_1_2_1_
Cell parameters						
a, b, c, Å	63.6, 66.3, 91.4	64.7, 66.0, 87.8	61.8, 65.9, 91.2	60.0, 65.2, 87.3	67.7, 66.9, 92.2	194.5, 86.6, 57.7
α, β, γ, °	90, 90, 90	90, 90, 90	90, 90, 90	90, 909, 90	90, 90, 90	90, 90, 90
Molecules/a.u.	1	1	1	1	1	1
Resolution, (Å)	50–2.4 (2.44–2.4)	50–3.5 (3.69–3.5)	50–2.4 (2.44–2.4)	50–2.85 (2.9–2.85)	50–2.19 (2.23–2.19)	50–2.6 (2.64–2.6)
# of reflections						
Total	49,666	11,650	61,106	34,488	86,920	39,768
Unique	13,796	4021	14,904	7185	20,214	10,748
R_merge_^a^, %	9.1 (57.9)	22.3 (113)	10.6 (80.1)	12.1 (104)	17.2 (66.5)	15.4 (98.3)
R_pim_^b^, %	5.1 (32.3)	14.7 (72.9)	5.8 (71.1)	8.2 (77.4)	9.7 (36.4)	9.0 (65.0)
*CC*_1/2_ ^c^	0.98 (0.71)	0.97 (0.33)	1.0 (0.50)	1.0 (0.42)	1.0 (0.65)	0.94 (0.60)
I/σ	12.3 (1.3)	2.8 (0.6)	14.2 (0.8)	12.5 (0.8)	25.5 (2.1)	17.3 (1.1)
Completeness, %	88.3 (78.2)	80.6 (83.9)	97.8 (86.4)	83.3 (78.0)	97.4 (85.0)	86.1 (90.2)
Redundancy	3.6 (3.7)	2.9 (3.0)	4.1 (2.7)	4.8 (4.2)	4.3 (3.8)	3.7 (3.1)
**Refinement Statistics**						
Resolution, Å	50.0–2.4	50.0–3.5	50.0–2.4	50.0–2.85	50.0–2.19	50.0–2.6
R ^d^ %	22.1	25.6	24.0	24.2	20.8	22.1
R_free_^e^, %	27.4	30.7	26.7	29.6	25.5	27.4
# of atoms						
Protein	2,668	2,640	2,665	2,675	2,683	2,668
Water	32	–	23	–	64	2
Ligand/Ion	212	184	211	186	193	186
Overall B value (Å)^2^						
Protein	52	82	62	73	53	85
Water	48	–	56	–	49	74
Ligand/Ion	70	96	74	86	63	102
RMSD ^f^						
Bond lengths, Å	0.005	0.004	0.006	0.009	0.007	0.012
Bond angles, °	1.0	0.79	0.98	1.3	1.0	1.4
Ramachandran ^g^						
Favored, %	94.0	90.2	94.0	89.5	95.0	92.5
Allowed, %	4.2	7.4	5.4	9.0	4.7	7.2
Outliers, %	1.8	2.4	0.6	1.5	0.3	0.3
PDB ID	8GD0	8GJT	8GCZ	8GD1	8GD3	8GD5

Values in parentheses are for highest-resolution shell; ^a^ *R*_merge_ = ∑│*I* − <*I*>│/∑*I*, where *I* is the observed intensity and <*I*> is the average intensity obtained from multiple observations of symmetry-related reflections after rejections; ^b^ R_pim_ = as defined in [26]; ^c^ *CC*_1/2_ = as defined by Karplus and Diederichs [27]; ^d^ *R* = ∑║F_o_│ − │ F_c_║/∑│F_o_│, where F_o_ and F_c_ are the observed and calculated structure factors, respectively; ^e^ R_free_ = as defined by Brünger [28]; ^f^ RMSD = root mean square deviation; ^g^ calculated with MolProbity.

**Table 2:** **Viral neutralization**. Neutralization of VSV-G, HIV-1_CH58TF_, HIV-1_JRFL_, or HIV-1_AD8_ pseudoviruses are shown as IC_50_ (average ± SD) of at least three independent experiments; IC_50_ values are in µM. CD4mc IC_50_.

	VSV-G Pseudovirus	HIV-1_CH58TF_	HIV-1_JRFL_	HIV-1_AD8_
**DMSO**	>100	>100	>100	>100
**BNM-III-170**	>100	0.033 ± 0.01	13.49 ± 6.65	3.75 ± 1.3
**MCG-IV-210**	>100	3.46 ± 1.06	>100	>100
**TFH-I-070-A6**	>100	0.45 ± 0.09	67.24 ± 30	9.59 ± 3.4
**TFH-I-116-D1**	>100	0.065 ± 0.01	43.82 ± 16.6	4.8 ± 1.7
**ZXC-I-090**	>100	2.63 ± 0.5	>100	>100
**ZXC-I-092**	>100	0.75 ± 0.12	54.22 ± 22.8	34.05 ± 13.8
**DL-I-101**	>100	0.16 ± 0.03	>100	10.21 ± 3.1
**DL-I-102**	>100	0.66 ± 0.25	>100	87.02 ± 39

## Data Availability

Data are contained within the article and Supplementary Materials.

## Supplementary Materials

The following supporting information can be downloaded at: https://www.mdpi.com/article/10.3390/v15051185/s1. Reference [43] is cited in the supplementary materials.

## References

[1] Allan J.S., Coligan J.E., Barin F., McLane M.F., Sodroski J.G., Rosen C.A., Haseltine W.A., Lee T.H., Essex M. (1985). Major glycoprotein antigens that induce antibodies in AIDS patients are encoded by HTLV-III. Science.

[2] Robey W.G., Safai B., Oroszlan S., Arthur L.O., Gonda M.A., Gallo R.C., Fischinger P.J. (1985). Characterization of envelope and core structural gene products of HTLV-III with sera from AIDS patients. Science.

[3] Wyatt R., Sodroski J. (1998). The HIV-1 envelope glycoproteins: Fusogens, antigens, and immunogens. Science.

[4] Furuta R.A., Wild C.T., Weng Y., Weiss C.D. (1998). Capture of an early fusion-active conformation of HIV-1 gp41. Nat. Struct. Biol..

[5] Decker J.M., Bibollet-Ruche F., Wei X., Wang S., Levy D.N., Wang W., Delaporte E., Peeters M., Derdeyn C.A., Allen S. (2005). Antigenic conservation and immunogenicity of the HIV coreceptor binding site. J. Exp. Med..

[6] Veillette M., Coutu M., Richard J., Batraville L.A., Dagher O., Bernard N., Tremblay C., Kaufmann D.E., Roger M., Finzi A. (2015). The HIV-1 gp120 CD4-bound conformation is preferentially targeted by antibody-dependent cellular cytotoxicity-mediating antibodies in sera from HIV-1-infected individuals. J. Virol..

[7] Veillette M., Desormeaux A., Medjahed H., Gharsallah N.E., Coutu M., Baalwa J., Guan Y., Lewis G., Ferrari G., Hahn B.H. (2014). Interaction with cellular CD4 exposes HIV-1 envelope epitopes targeted by antibody-dependent cell-mediated cytotoxicity. J. Virol..

[8] Madani N., Schon A., Princiotto A.M., Lalonde J.M., Courter J.R., Soeta T., Ng D., Wang L., Brower E.T., Xiang S.H. (2008). Small-molecule CD4 mimics interact with a highly conserved pocket on HIV-1 gp120. Structure.

[9] Madani N., Princiotto A.M., Schon A., LaLonde J., Feng Y., Freire E., Park J., Courter J.R., Jones D.M., Robinson J. (2014). CD4-mimetic small molecules sensitize human immunodeficiency virus to vaccine-elicited antibodies. J. Virol..

[10] Prevost J., Tolbert W.D., Medjahed H., Sherburn R.T., Madani N., Zoubchenok D., Gendron-Lepage G., Gaffney A.E., Grenier M.C., Kirk S. (2020). The HIV-1 Env gp120 Inner Domain Shapes the Phe43 Cavity and the CD4 Binding Site. mBio.

[11] Kwong P.D., Wyatt R., Robinson J., Sweet R.W., Sodroski J., Hendrickson W.A. (1998). Structure of an HIV gp120 envelope glycoprotein in complex with the CD4 receptor and a neutralizing human antibody. Nature.

[12] Zhao Q., Ma L., Jiang S., Lu H., Liu S., He Y., Strick N., Neamati N., Debnath A.K. (2005). Identification of N-phenyl-N′-(2,2,6,6-tetramethyl-piperidin-4-yl)-oxalamides as a new class of HIV-1 entry inhibitors that prevent gp120 binding to CD4. Virology.

[13] Madani N., Princiotto A.M., Mach L., Ding S., Prevost J., Richard J., Hora B., Sutherland L., Zhao C.A., Conn B.P. (2018). A CD4-mimetic compound enhances vaccine efficacy against stringent immunodeficiency virus challenge. Nat. Commun..

[14] Madani N., Princiotto A.M., Zhao C., Jahanbakhshsefidi F., Mertens M., Herschhorn A., Melillo B., Smith A.B., Sodroski J. (2017). Activation and Inactivation of Primary Human Immunodeficiency Virus Envelope Glycoprotein Trimers by CD4-Mimetic Compounds. J. Virol..

[15] Ding S., Verly M.M., Princiotto A., Melillo B., Moody T., Bradley T., Easterhoff D., Roger M., Hahn B.H., Madani N. (2016). Small Molecule CD4-Mimetics Sensitize HIV-1-infected Cells to ADCC by Antibodies Elicited by Multiple Envelope Glycoprotein Immunogens in Non-Human Primates. AIDS Res. Hum. Retrovir..

[16] Richard J., Prevost J., von Bredow B., Ding S., Brassard N., Medjahed H., Coutu M., Melillo B., Bibollet-Ruche F., Hahn B.H. (2017). BST-2 Expression Modulates Small CD4-Mimetic Sensitization of HIV-1-Infected Cells to Antibody-Dependent Cellular Cytotoxicity. J. Virol..

[17] Richard J., Veillette M., Brassard N., Iyer S.S., Roger M., Martin L., Pazgier M., Schon A., Freire E., Routy J.P. (2015). CD4 mimetics sensitize HIV-1-infected cells to ADCC. Proc. Natl. Acad. Sci. USA.

[18] Ding S., Grenier M.C., Tolbert W.D., Vezina D., Sherburn R., Richard J., Prevost J., Chapleau J.P., Gendron-Lepage G., Medjahed H. (2019). A New Family of Small-Molecule CD4-Mimetic Compounds Contacts Highly Conserved Aspartic Acid 368 of HIV-1 gp120 and Mediates Antibody-Dependent Cellular Cytotoxicity. J. Virol..

[19] Kwon Y.D., Finzi A., Wu X., Dogo-Isonagie C., Lee L.K., Moore L.R., Schmidt S.D., Stuckey J., Yang Y., Zhou T. (2012). Unliganded HIV-1 gp120 core structures assume the CD4-bound conformation with regulation by quaternary interactions and variable loops. Proc. Natl. Acad. Sci. USA.

[20] Zoubchenok D., Veillette M., Prevost J., Sanders-Buell E., Wagh K., Korber B., Chenine A.L., Finzi A. (2017). Histidine 375 Modulates CD4 Binding in HIV-1 CRF01_AE Envelope Glycoproteins. J. Virol..

[21] Grenier M.C., Ding S., Vezina D., Chapleau J.P., Tolbert W.D., Sherburn R., Schon A., Somisetti S., Abrams C.F., Pazgier M. (2020). Optimization of Small Molecules That Sensitize HIV-1 Infected Cells to Antibody-Dependent Cellular Cytotoxicity. ACS Med. Chem. Lett..

[22] Mascola J.R., D’Souza P., Gilbert P., Hahn B.H., Haigwood N.L., Morris L., Petropoulos C.J., Polonis V.R., Sarzotti M., Montefiori D.C. (2005). Recommendations for the design and use of standard virus panels to assess neutralizing antibody responses elicited by candidate human immunodeficiency virus type 1 vaccines. J. Virol..

[23] Seaman M.S., Janes H., Hawkins N., Grandpre L.E., Devoy C., Giri A., Coffey R.T., Harris L., Wood B., Daniels M.G. (2010). Tiered categorization of a diverse panel of HIV-1 Env pseudoviruses for assessment of neutralizing antibodies. J. Virol..

[24] Hraber P., Korber B., Wagh K., Montefiori D., Roederer M. (2018). A single, continuous metric to define tiered serum neutralization potency against HIV. eLife.

[25] Montefiori D.C., Roederer M., Morris L., Seaman M.S. (2018). Neutralization tiers of HIV-1. Curr. Opin. HIV AIDS.

[26] Weiss M.S. (2001). Global indicators of X-ray data quality. J. Appl. Cryst..

[27] Karplus P.A., Diederichs K. (2012). Linking crystallographic model and data quality. Science.

[28] Brunger A.T. (1997). Free *R* value: Cross-validation in crystallography. Methods in Enzymology.

[29] Melillo B., Liang S., Park J., Schon A., Courter J.R., LaLonde J.M., Wendler D.J., Princiotto A.M., Seaman M.S., Freire E. (2016). Small-Molecule CD4-Mimics: Structure-Based Optimization of HIV-1 Entry Inhibition. ACS Med. Chem. Lett..

[30] Richard J., Pacheco B., Gohain N., Veillette M., Ding S., Alsahafi N., Tolbert W.D., Prevost J., Chapleau J.P., Coutu M. (2016). Co-receptor Binding Site Antibodies Enable CD4-Mimetics to Expose Conserved Anti-cluster A ADCC Epitopes on HIV-1 Envelope Glycoproteins. EBioMedicine.

[31] Alsahafi N., Bakouche N., Kazemi M., Richard J., Ding S., Bhattacharyya S., Das D., Anand S.P., Prevost J., Tolbert W.D. (2019). An Asymmetric Opening of HIV-1 Envelope Mediates Antibody-Dependent Cellular Cytotoxicity. Cell Host Microbe.

[32] Laumaea A., Marchitto L., Ding S., Beaudoin-Bussieres G., Prevost J., Gasser R., Chatterjee D., Gendron-Lepage G., Medjahed H., Chen H.C. (2023). Small CD4 mimetics sensitize HIV-1-infected macrophages to antibody-dependent cellular cytotoxicity. Cell Rep..

[33] Prevost J., Anand S.P., Rajashekar J.K., Zhu L., Richard J., Goyette G., Medjahed H., Gendron-Lepage G., Chen H.C., Chen Y. (2022). HIV-1 Vpu restricts Fc-mediated effector functions in vivo. Cell Rep..

[34] Rajashekar J.K., Richard J., Beloor J., Prevost J., Anand S.P., Beaudoin-Bussieres G., Shan L., Herndler-Brandstetter D., Gendron-Lepage G., Medjahed H. (2021). Modulating HIV-1 envelope glycoprotein conformation to decrease the HIV-1 reservoir. Cell Host Microbe.

[35] Richard J., Prevost J., Baxter A.E., von Bredow B., Ding S., Medjahed H., Delgado G.G., Brassard N., Sturzel C.M., Kirchhoff F. (2018). Uninfected Bystander Cells Impact the Measurement of HIV-Specific Antibody-Dependent Cellular Cytotoxicity Responses. mBio.

[36] Ding S., Veillette M., Coutu M., Prevost J., Scharf L., Bjorkman P.J., Ferrari G., Robinson J.E., Sturzel C., Hahn B.H. (2016). A Highly Conserved Residue of the HIV-1 gp120 Inner Domain Is Important for Antibody-Dependent Cellular Cytotoxicity Responses Mediated by Anti-cluster A Antibodies. J. Virol..

[37] Madani N., Princiotto A.M., Easterhoff D., Bradley T., Luo K., Williams W.B., Liao H.X., Moody M.A., Phad G.E., Vazquez Bernat N. (2016). Antibodies Elicited by Multiple Envelope Glycoprotein Immunogens in Primates Neutralize Primary Human Immunodeficiency Viruses (HIV-1) Sensitized by CD4-Mimetic Compounds. J. Virol..

[38] Finzi A., Xiang S.H., Pacheco B., Wang L., Haight J., Kassa A., Danek B., Pancera M., Kwong P.D., Sodroski J. (2010). Topological layers in the HIV-1 gp120 inner domain regulate gp41 interaction and CD4-triggered conformational transitions. Mol. Cell.

[39] Richard J., Veillette M., Batraville L.A., Coutu M., Chapleau J.P., Bonsignori M., Bernard N., Tremblay C., Roger M., Kaufmann D.E. (2014). Flow cytometry-based assay to study HIV-1 gp120 specific antibody-dependent cellular cytotoxicity responses. J. Virol. Methods.

[40] Otwinowski Z., Minor W., Carter C.W.J., Sweet R.M. (1997). Processing of X-ray diffraction data collected in oscillation mode. Meth Enzymol.

[41] Collaborative Computational Project (1994). The CCP4 suite: Programs for protein crystallography. Acta Crystallogr. Sect. D Biol. Crystallogr..

[42] Adams P.D., Afonine P.V., Bunkoczi G., Chen V.B., Davis I.W., Echols N., Headd J.J., Hung L.W., Kapral G.J., Grosse-Kunstleve R.W. (2010). PHENIX: A comprehensive Python-based system for macromolecular structure solution. Acta Cryst. D Biol Cryst..

[43] Laskowski R.A., Swindells M.B. (2011). LigPlot+: Multiple ligand-protein interaction diagrams for drug discovery. J. Chem. Inf. Model..

