# Supplementary material for: Piperidine CD4-Mimetic Compounds Expose Vulnerable Env Epitopes Sensitizing HIV-1-Infected Cells to ADCC
Source: Viruses. 2023 May 17;15(5):1185. doi: 10.3390/v15051185 (PMC10220648; doi:10.3390/v15051185)
Supplement: Supplementary file 1 [file viruses-15-01185-s001.zip › viruses-2367797-supplementary.pdf]

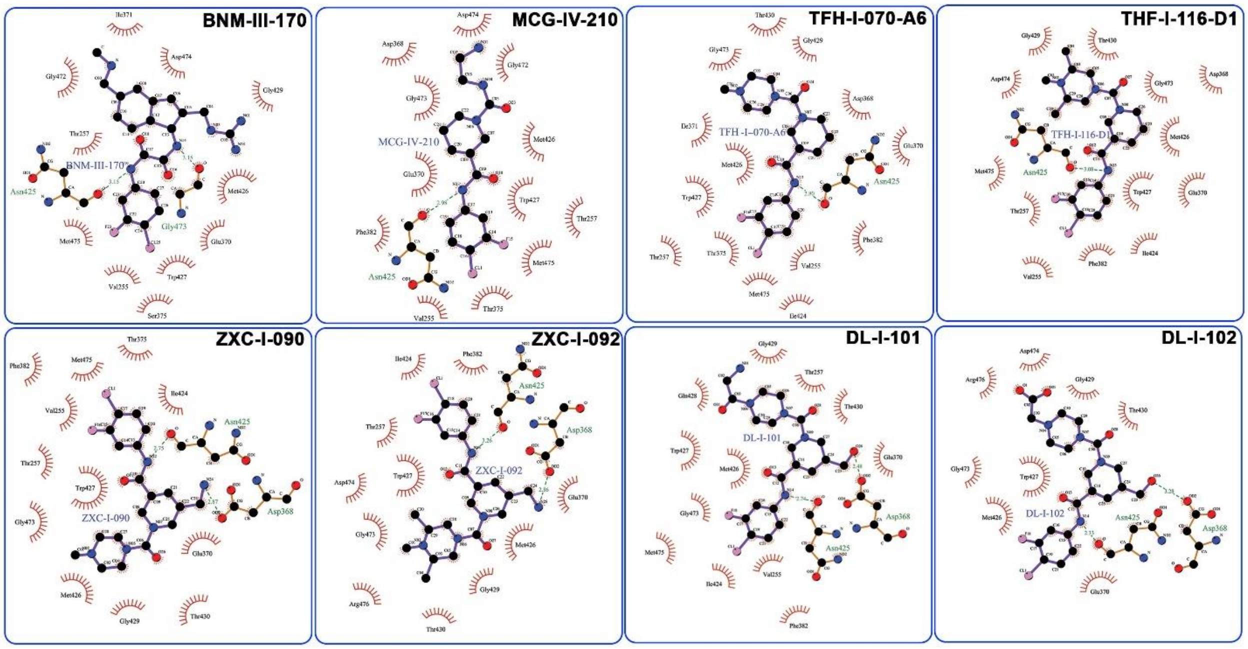

Figure S1. LigPlot^{+} [1] generated figures for each compound. Hydrogen bonds are as indicated and contact residues are shown within a 4 Å cutoff.

1. Laskowski, R. A.; Swindells, M. B., LigPlot+: multiple ligand-protein interaction diagrams for drug discovery. Journal of chemical information and modeling 2011, 51, (10), 2778-86.

Chemical Synthesis Flowchart

Synthetic Route to DL Series (DL-I-101 and DL-I-102)

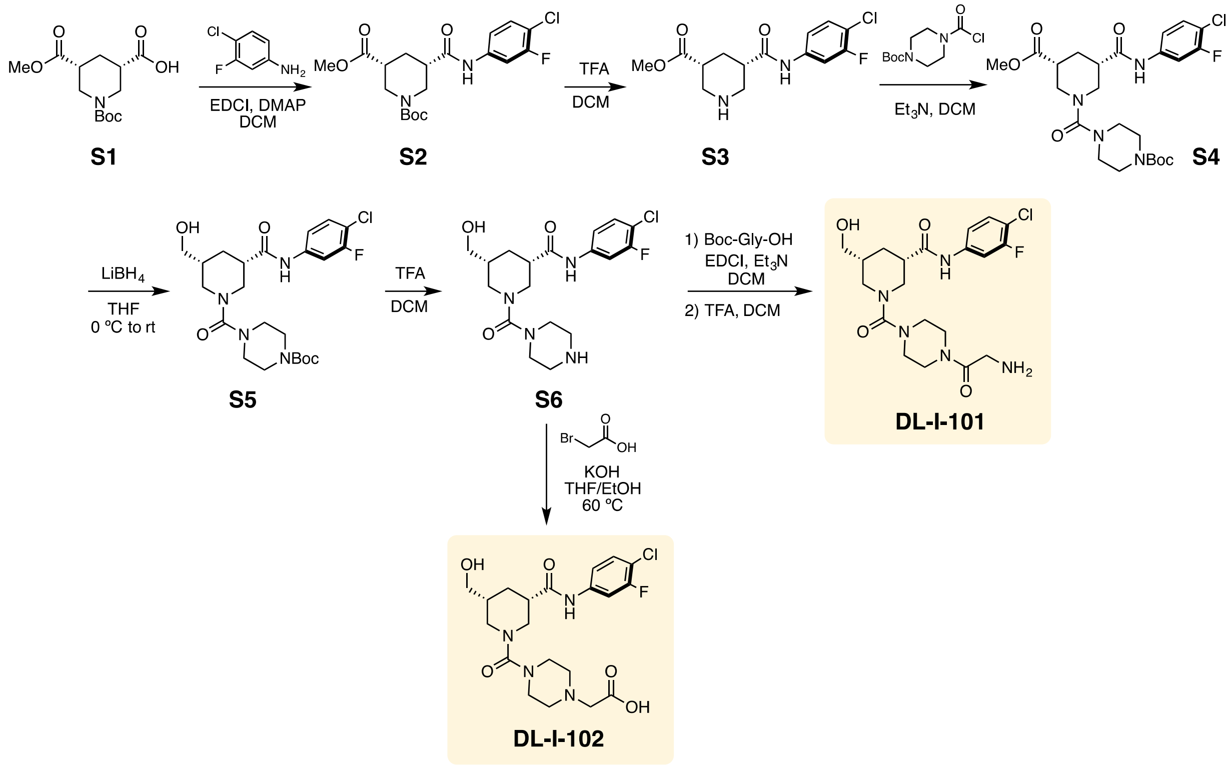

DL-I-101 and **DL-I-102** can be readily prepared from a common intermediate **S6**. Intermediate **S6** can be synthesized in 5 steps from a known piperidine derivative **S1**.

Compounds shaded in colored boxes [i.e., **DL-I-101** and **DL-I-102**] are the chemical structures of the synthesized products reported in this manuscript.

Synthetic Route to ZXC and TFH Series (ZXC-I-090, ZXC-I-092, TFH-II-128, and TFH-II-151)

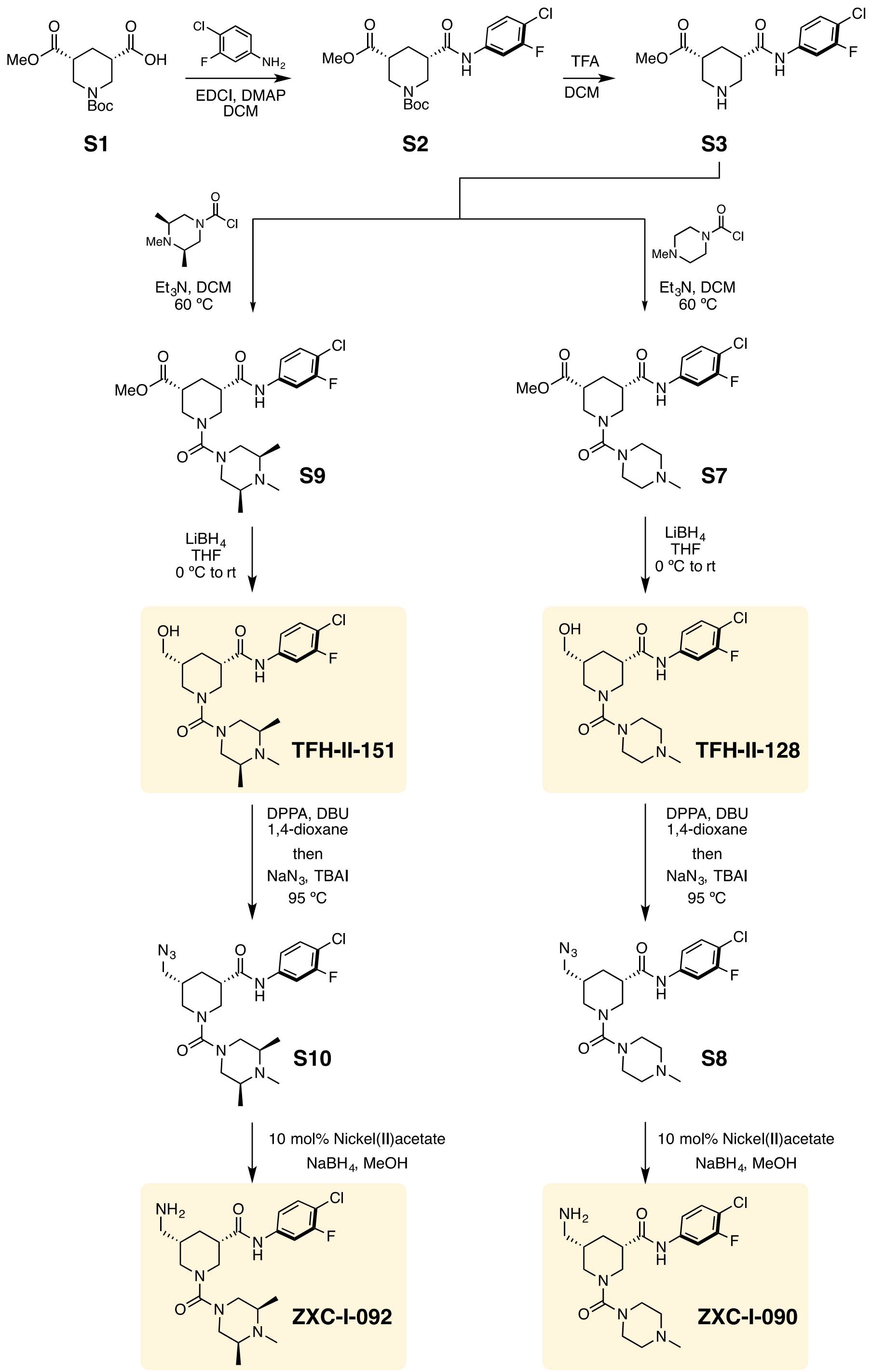

TFH-II-128, TFH-II-151, ZXC-I-090, and **ZXC-I-092** be prepared from a common intermediate **S3**. Intermediate **S3** can be synthesized in 2 steps from a known piperidine derivative **S1**.

Compounds shaded in colored boxes [i.e., **TFH-II-128, TFH-II-151, ZXC-I-090,** and **ZXC-I-092**] are the chemical structures of the synthesized products reported in this manuscript.

Synthetic Route to TFH-I-116-D1 and TFH-I-070-A6

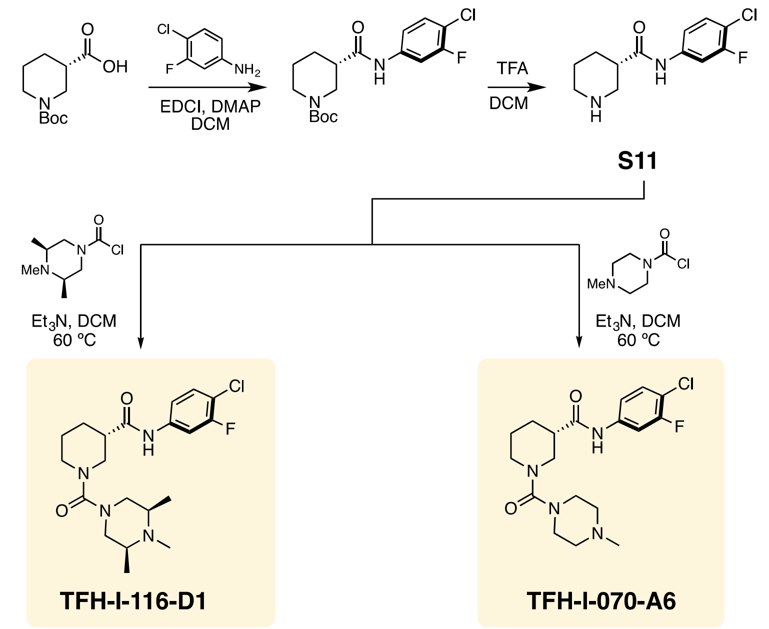

TFH-I-070-A6, and **TFH-I-116-D1** is prepared from a common intermediate **S11**. Intermediate **S3** can be synthesized in 2 steps from a known piperidine derivative (*S*)-1-(*tert*-butoxycarbonyl)piperidine-3-carboxylic acid.

Compounds shaded in colored boxes [i.e., **TFH-I-070-A6** and **TFH-I-116-D1**] are the chemical structures of the synthesized products reported in this manuscript.

Supplemental Experimental Methods

General Experimental Protocols:

Reactions performed under anhydrous conditions were conducted in oven-dried glassware under an inert atmosphere of argon, unless otherwise stated. Commercial sources of chloroform (ethanol-stabilized), methylene chloride (ethanol-stabilized), toluene, tetrahydrofuran, and diethyl ether were dried over CaH_2, distilled under reduced pressure, and stored over 4Å molecular sieves under an argon atmosphere. All reagents were purchased from commercial sources and used as received. Reaction mixtures were magnetically stirred under an argon atmosphere, unless otherwise noted, reactions were monitored by either thin-layer chromatography (TLC) with 250-μm SiliaPlate® precoated TLC plates or Waters® ACQUITY analytical ultraperformance liquid chromatography (UPLC) system. Flash chromatography was done on SiliaFlash® silica gel (230-400 mesh). Yields refer to chromatographically isolated and spectroscopically pure compounds. Optical rotations were measured on a Jasco P-2000 polarimeter.

Nuclear magnetic resonance (NMR) spectra (^{1}H and ^{13}C) were recorded on a Bruker AX-500 or NEO-600 NMR spectrometer. ^{1}H chemical shifts of samples in $CDCl_3$ or CD_3OD are referenced to proton atom in $CDCl_3$ (δ 7.26) or proton atom in CD_3OD (δ 3.31) respectively. Spectral data are reported using the following format: chemical shift (ppm) [multiplicity, coupling constant(s) (in Hz), and integral (to the nearest whole integer)]. ^{13}C NMR chemical shifts are referenced to the carbon atom in $CDCl_3$ (δ = 77.16) or CD_3OD (δ = 49.00).

Infrared (IR) spectra were recorded with a Jasco spectrometer (model 480 plus). The samples were deposited as thin films (solids by evaporation of a DCM solution; liquids by direct deposit) on a cell plate window. Absorption peak maxima are reported in cm^{1}.

High-resolution mass spectrometry (HRMS) was carried out at the University of Pennsylvania Mass Spectroscopy Service Center on either a (i) Waters LCT Premier XE liquid chromatography-mass spectrometry (LC-MS) system or a (ii) Waters GC-TOF Premier system.

Preparative-scale HPLC was performed with a Gilson® SPE Purification system equipped with a Sunfire C_{18} OBD column (10-μm packing material, 30- by 150-mm column dimensions), a 215 liquid handler, a 333 binary gradient module, a 156 UV-visible (UV-Vis) dual-wavelength (254- and 365-nm) detector, and Trilution® 3.0 software. Purification solvent systems were comprised of H_2O (HPLC-grade) and acetonitrile (HPLC-grade) containing 0.1% trifluoroacetic acid.

Experimental Details and Characterization Data

1-(*Tert*-butyl) 3-Methyl (3*R*,5*S*)-5-((4-chloro-3-fluorophenyl)carbamoyl)piperidine-1,3-dicarboxylate (S2)

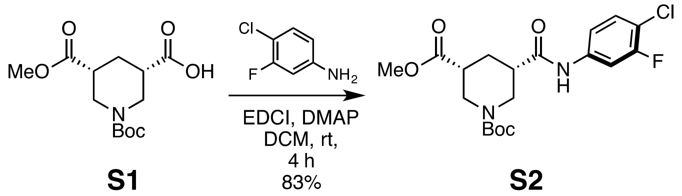

To a solution of **S1** (220 mg, 0.77 mmol) in DCM (0.5 M) was added 4-chloro-3-fluoroaniline (110 mg, 0.77 mmol), 1-ethyl-3-(3-dimethylaminopropyl)carbodiimide (131 mg, 0.84 mmol), and 4-dimethylaminopyridine (103 mg, 0.84 mmol). The reaction mixture was stirred for 4 hours at room temperature. The crude mixture was analyzed by LCMS to confirm the formation of the desired product. The reaction mixture was washed with 2M HCl (2 mL) and extracted with additional DCM (2 x 10 mL). The organic layers were combined, washed with brine (10 mL), dried ($MgSO_4$), and concentrated. The resulting residue was purified by flash column chromatography (DCM:MeOH, 9:1) to afford **S2** (255 mg, 0.64 mmol, 83%) as a white amorphous solid.

^{1}H NMR (600 MHz, MeOD) δ 7.68 (dd, *J* = 11.5, 2.4 Hz, 1H), 7.34 (t, *J* = 8.5 Hz, 1H), 7.23 (ddd, 8.7, 2.4, 1.1 Hz, 1H), 4.38 – 4.15 (br m, 2H), 3.69 (s, 3H), 3.02 – 2.64 (br m, 2H), 3.24 – 3.13 (m, 5H), 2.57 – 2.48 (m, 2H), 2.31 (d, *J* = 12.6 Hz, 3H), 1.88 – 1.77 (dddd, *J* = 10.5, 10.5, 3.8, 3.8 Hz, 1H), 2.09 – 2.05 (m, 1H), 1.86 – 1.79 (m, 1H), and 1.47 (s, 9H).

^{13}C NMR (151 MHz, MeOD) δ 172.8, 172.0, 158.5 (d, J_{CF} = 245.8 Hz), 154.8, 138.9 (d, J_{CF} = 9.3 Hz), 130.1, 115.9 (d, J_{CF} = 3.5 Hz), 114.7 (d, J_{CF} = 26.5 Hz), 107.9, 107.8, 80.4, 51.1, 45.5, 45.2 44.8, 42.9, 40.4, 30.3, and 27.2.

IR (neat): 2977, 2914, 2852, 2419, 1736, 1661, 1495, 1412, 1254, 1145, 1064, 1020, 970, 930, 894, and 863 cm $^{-1}$.

HRMS (ESI): Calcd for $C_{19}H_{25}ClFN_2O_5^+$ [M+H]$^+$: 415.1431; Found 415.1436.

$[\alpha]_D^{23}$ -22.64 (c 1.00, MeOH)

Methyl (3*R*,5*S*)-5-((4-chloro-3-fluorophenyl)carbamoyl)piperidine-3-carboxylate (S3)

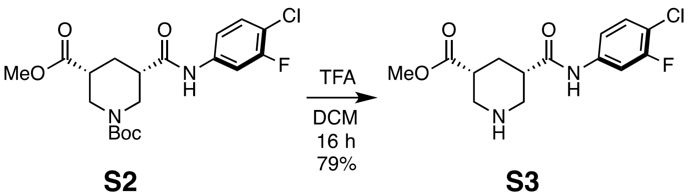

S2 **S3**

To a solution of **S2** (200 mg, 0.48 mmol) in DCM (0.5 M) under argon atmosphere was added trifluoroacetic acid (0.4 mL, 4.82 mmol). This reaction mixture was stirred for 16 hours at room temperature. The crude mixture was analyzed by LCMS to confirm the formation of the desired product. The reaction mixture was neutralized with aqueous saturated K_2CO_3 (10 mL), and the resulting mixture was extracted with DCM (3 x 10 mL). The organic layers were combined, washed with brine (10 mL), dried ($MgSO_4$), and concentrated. The resulting residue was purified by flash column chromatography (DCM:MeOH, 9:1) to afford **S3** (120 mg, 0.38 mmol, 79%) as a white amorphous solid.

^{1}H NMR (600 MHz, MeOD) δ 7.68 (dd, *J* = 11.6, 2.4 Hz, 1H), 7.35 (t, *J* = 8.5 Hz, 1H), 7.24 (ddd, 8.8, 2.4, 1.1 Hz, 1H), 3.66 (s, 3H), 3.24 – 3.19 (m, 1H), 3.15 – 3.10 (m, 1H), 2.67 (dd, *J* = 12.6, 11.1 Hz, 1H), 2.63 – 2.57 (m, 2H), 2.57 – 2.50 (m, 2H), 2.24 (m, 1H), and 1.81 (m, 1H).

^{13}C NMR (151 MHz, MeOD) δ 175.1, 174.4, 159.9 (d, J_{CF} = 245.7 Hz), 140.4 (d, J_{CF} = 10.5 Hz), 131.5, 117.3 (d, J_{CF} = 3.5 Hz), 115.9 (d, J_{CF} = 25.8 Hz), 109.1 (d, J_{CF} = 26.5 Hz), 52.3, 48.8, 47.4, 45.1, 42.6, and 31.3.

IR (neat): 3316, 2959, 2910, 2852, 2365, 1727, 1672, 1605, 1537, 1493, 1421, 1315, 1263, 1193, 1064, 868, and 814 cm^{-1}.

HRMS (ESI): Calcd for $C_{14}H_{17}ClFN_2O_3^+$ $[M+H]^+$: 315.0912; Found 315.0931.

$[\alpha]_D^{23}$ -3.63 (c 1.00, MeOH)

Tert-butyl 4-((3*S*,5*R*)-3-((4-chloro-3-fluorophenyl)carbamoyl)-5-(methoxycarbonyl)piperidine-1-carbonyl)piperazine-1-carboxylate (S4)

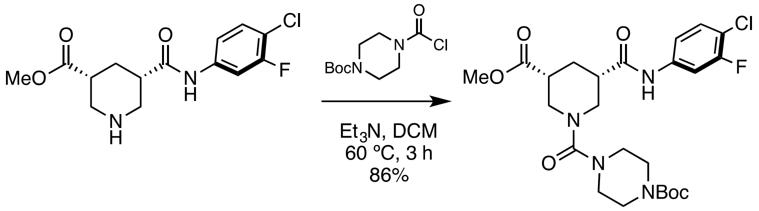

S3 **S4**

To a solution of **S3** (567.2 mg, 1.8 mmol) in DCM (0.5 M) under argon atmosphere was added *t*ert-butyl 4-(chlorocarbonyl)piperazine-1-carboxylate (582.3 mg, 2.3 mmol) and triethylamine (0.5 mL, 3.6 mmol). The resulting mixture was heated to 60 ºC and stirred for 3 h, then allowed to cool to room temperature and concentrated in vacuo. The crude residue was diluted in DCM (10 mL), saturated aqueous NH_4Cl (10 mL) and the mixture extracted with DCM (3 x 10 mL). The organic layers were combined, washed with brine (15 mL), dried ($MgSO_4$), and concentrated. The resulting residue was purified by flash column chromatography (DCM:MeOH, 9:1) to afford **S4** (814.6 mg, 1.5 mmol, 86%) as a white amorphous solid.

^{1}H NMR (600 MHz, MeOD) δ 7.75 (dd, *J* = 11.6, 2.4 Hz, 1H), 7.41 (t, *J* = 8.5 Hz, 1H), 7.32 – 7.25 (m, 1H), 4.03 – 3.92 (m, 1H), 3.90 – 3.82 (m, 1H), 3.47 (br s, 6H), 2.99 (ddd, *J* = 26.8, 13.3, 11.4 Hz, 2H), 2.68 (m, 2H), 2.49 – 2.28 (m, 1H), 1.90 (q, *J* = 12.6 Hz, 1H), and 1.50 (s, 12H)

^{13}C NMR (151 MHz, MeOD) δ 174.3, 173.4, 165.2, 159.9 (d, J_{CF} = 245.0 Hz), 156.3, 140.3, (d, J_{CF} = 10.7 Hz), 131.5, 117.3, (d, J_{CF} = 3.1 Hz), 116.1 (d, J_{CF} = 17.7 Hz), 109.3, 109.1, 81.5, 52.5, 49.7, 49.5, 47.8, 47.7, 45.1, 44.5, 44.0, 43.7, 41.5, 31.6, and 28.6.

IR (neat): 3314, 2925, 2856, 1735, 1696, 1607, 1539, 1480, 1420, 1366, 1243, 1167, 1126, 1087, 1001, 912, and 862 cm^{-1}.

HRMS (ESI): Calcd for $C_{24}H_{33}ClFN_4O_6^+$ [M+H]$^+$: 527.2073; Found 527.2059.

$[\alpha]_D^{23}$ -1.55 (c 1.00, MeOH)

Tert-butyl 4-((3*S*,5*R*)-3-((4-chloro-3-fluorophenyl)carbamoyl)-5-(hydroxymethyl)piperidine-1-carbonyl)piperazine-1-carboxylate (S5)

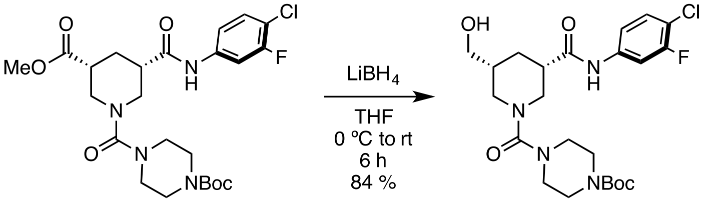

S4 **S5**

To a cooled (0 ºC) solution of **S4** (960 mg, 1.8 mmol) in anhydrous THF (0.5 M) under argon atmosphere was added lithium borohydride (2 M in THF, 9.1 mL, 18.2 mmol). The resulting mixture was stirred at 0 ºC for 1 hour, then warm to room temperature and stirred for additional 5 hours. The crude mixture was analyzed by LCMS to confirm the formation of the desired product. Acetic acid (0.1 mL) in hexanes (2 mL) was added to the reaction mixture over 10 min and concentrated. The resulting residue was diluted with Et_2O (10 mL) and saturated aqueous NH_4Cl (10 mL) then extracted with Et_2O (3 x 10 mL). The organic layers were combined, washed with brine (10 mL), dried ($MgSO_4$), and concentrated. The resulting residue was purified by flash column chromatography (DCM:MeOH, 8:2) to afford **S5** (763 mg, 1.5 mmol, 84%) as a white amorphous solid.

^{1}H NMR (600 MHz, MeOD) δ 7.70 (dd, *J* = 11.6, 2.4 Hz, 1H), 7.37 (t, *J* = 8.5 Hz, 1H), 7.25 (ddd, *J* = 8.8, 2.4, 1.1 Hz, 1H), 3.87 (m, 3H), 3.50 (dd, *J* = 11.0, 5.2 Hz, 1H), 3.47 – 3.42 (br m, 4H), 3.41 (dd, *J* = 11.0, 7.4 Hz, 1H), 3.33 – 3.20 (m, 4H), 2.90 (dd, *J* = 13.0, 11.4 Hz, 1H), 2.68 – 2.56 (m, 2H), 2.03 (dt, *J* = 12.8, 1.8 Hz, 1H), 1.82 – 1.69 (m, 1H), and 1.46 (s, 9H).

^{13}C NMR (151 MHz, MeOD) δ 174.3, 165.4, 159.9 (d, J_{CF} = 246.9 Hz),156.4, 140.4 (d, J_{CF} = 12.4 Hz), 131.5, 117.3 (d, J_{CF} = 3.5 Hz), 116.0 (d, J_{CF} = 18.1 Hz), 109.3 (d, J_{CF} = 26.1 Hz), 81.5, 65.4, 51.6, 49.8, 49.5, 47.9, 45.1, 44.7, 43.9, 39.4, 32.1, 30.1 and 28.6.

IR (neat): 3424, 2980, 2917, 2855, 2399, 1665, 1615, 1498, 1241, 1163, 1000, and 866 cm $^{-1}$.

HRMS (ESI): Calcd for $C_{23}H_{33}ClFN_4O_5^+$ $[M+H]^+$: 499.2124; Found 499.2099.

$[\alpha]_D^{23}$ -1.55 (c 1.00, MeOH)

(3S,5R)-N-(4-Chloro-3-fluorophenyl)-5-(hydroxymethyl)-1-(piperazine-1-carbonyl)piperidine-3-carboxamide (S6)

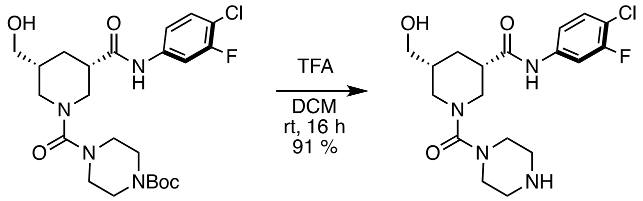

S5 **S6**

To a solution of **S5** (1.21 g, 2.4 mmol) in DCM (0.5 M) under argon atmosphere was added trifluoroacetic acid (1.84 mL, 24.0 mmol). This reaction mixture was stirred for 16 hours at room temperature. The crude mixture was analyzed by LCMS to confirm the formation of the desired product. The reaction mixture was neutralized with aqueous saturated K_2CO_3 (10 mL), and the resulting mixture was extracted with DCM (3 x 20 mL). The organic layers were combined, washed with brine (20 mL), dried ($MgSO_4$), and concentrated. The resulting residue was purified by flash column chromatography (DCM:MeOH, 8:2) to afford **S6** (873 mg, 2.2 mmol, 91%) as a white amorphous solid.

^{1}H NMR (600 MHz, MeOD) δ 7.71 (dd, J = 11.6, 2.4 Hz, 1H), 7.38 (t, J = 8.5 Hz, 1H), 7.25 (ddd, J = 8.7, 2.4, 1.1 Hz, 1H), 3.84 (dddd, J = 24.5, 13.1, 4.1, 2.0 Hz, 2H), 3.50 (dd, J = 11.0, 5.2 Hz, 1H), 3.40 (dd, J = 11.0, 7.4 Hz, 1H), 3.2 (m, 2H), 2.89 (dd, J = 13.0, 11.4 Hz, 1H), 2.59 (dd, J = 13.2, 11.6 Hz, 1H), 2.43 (t, J = 5.0 Hz, 5H), 2.30 (s, 3H), 2.01 (m, 1H), 1.82 – 1.72 (m, 1H), and 1.44 (q, J = 12.5 Hz, 1H)

^{13}C NMR (151 MHz, MeOD) δ 174.4, 165.6, 165.2, 159.9 (d, J_{CF} = 246.1 Hz), 140.4, (d, J_{CF} = 10.3 Hz), 131.5, 117.3 (d, J_{CF} = 3.1 Hz), 116.0 (d, J_{CF} = 18.0 Hz), 109.3 (d, J_{CF} = 26.1 Hz), 65.4, 55.6, 51.6, 48.6, 47.4, 46.1, 44.7, 39.4, and 32.1.

IR (neat): 2914, 2852, 1690, 1601, 1542, 1480, 1422, 1288, 1247, 1145, 1069, and 997 cm $^{-1}$.

HRMS (ESI): Calcd for $C_{18}H_{25}ClFN_4O_3^+$ [M+H]$^+$: 399.1594; Found 399.1620.

[α]$_D^{23}$ -14.11 (c 1.00, MeOH)

(*3*S,5*R*)-*N*-(4-Chloro-3-fluorophenyl)-1-(4-glycylpiperazine-1-carbonyl)-5-(hydroxymethyl)piperidine-3-carboxamide (DL-I-101)

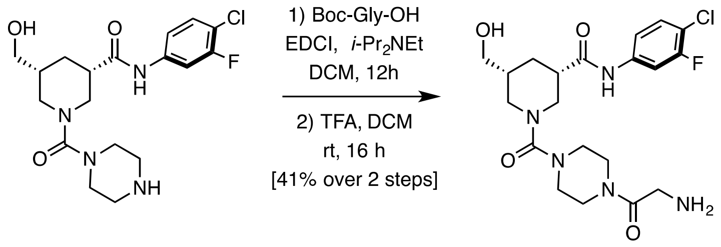

S6 **DL-I-101**

To a solution of **S6** (310 mg, 0.8 mmol) in DCM (0.8 M) under argon atmosphere was added N-(tert-butoxycarbonyl)glycine (341 mg, 1.9 mmol), 1-ethyl-3-(3-dimethylaminopropyl)carbodiimide (373 mg, 1.9 mmol), and N,N-diisopropylethylamine (0.8 mL, 4.7 mmol). The reaction mixture was stirred for 12 hours at room temperature. The crude mixture was analyzed by LCMS to confirm the formation of the desired product then concentrated in vacuo and carried forward without additional purification. To the crude residue, was added DCM (20 mL) and trifluoroacetic acid (0.6 mL, 8.0 mmol). This reaction mixture was stirred for 16 hours at room temperature, at which time LCMS confirmed the formation of the desired product. The reaction mixture was neutralized with aqueous saturated K_2CO_3 (10 mL), and the resulting mixture was extracted with DCM (3 x 15 mL). The organic layers were combined, washed with brine (15 mL), dried ($MgSO_4$), and concentrated. The resulting residue was purified by flash column chromatography (DCM:MeOH, 7:3) to afford **DL-I-101** (150 mg, 0.3 mmol, 41% over 2 steps) as a white amorphous solid.

^{1}H NMR (500 MHz, MeOD) δ 7.70 (dd, *J* = 11.6, 2.4 Hz, 1H), 7.37 (t, *J* = 8.5 Hz, 1H), 7.25 (ddd, *J* = 8.8, 2.3, 1.1 Hz, 1H), 3.96 (s, 2H), 3.95 – 3.80 (m, 2H), 3.68 (t, *J* = 5.4 Hz, 2H), 3.57 – 3.39 (m, 4H), 3.39 – 3.33 (m, 6H), 2.93 (dd, *J* = 13.1, 11.5 Hz, 1H), 2.66 (m, 2H), 2.12 – 2.02 (m, 1H), 1.86 – 1.70 (m, 1H), and 1.58 – 1.37 (m, 1H)

^{13}C NMR (126 MHz, MeOD) δ 174.3, 165.8, 165.3, 160.1 (d, J_{CF} = 246.9 Hz), 140.4 (d, J_{CF} = 9.9 Hz), 131.6, 117.3 (d, J_{CF} = 3.7 Hz), 116.1 (d, J_{CF} = 17.0 Hz), 109.3 (d, J_{CF} = 27.8 Hz), 65.4, 51.5, 49.7, 49.5, 47.8, 45.3, 44.7, 42.9, 41.0, 39.5, and 32.1.

IR (neat): 3308, 2925, 2863, 1738, 1694, 1603, 1482, 1421, 1367, 1286, 1573, and 994 cm^{-1}.

HRMS (ESI): Calcd for $C_{20}H_{28}ClFN_5O_4^+$ [M+H$^+$]: 456.1814; found 456.1815.

$[\alpha]_D^{23}$ -21.68 (c 1.00, MeOH)

2-(4-((3*S*,5*R*)-3-((4-Chloro-3-fluorophenyl)carbamoyl)-5-(hydroxymethyl)piperidine-1-carbonyl)piperazin-1-yl)acetic acid (DL-I-102)

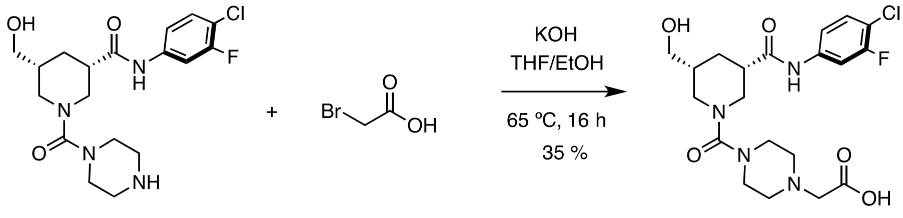

S6 **DL-I-102**

To a solution of **S6** (101 mg, 0.25 mmol) in THF/EtOH (4:1 v/v, 2.0 M) under argon atmosphere was added potassium hydroxide (42 mg, 0.75 mmol), and bromoacetic acid (21 uL, 0.29 mmol). The resulting mixture was heated to reflux and stirred for 16 h, then allowed to cool to room temperature and concentrated in vacuo. To the crude residue, was added DCM (5 mL) and DI water (5 mL), and the resulting mixture was extracted with DCM (3 x 5 mL). The organic layers were combined, washed with brine (5 mL), dried ($MgSO_4$), and concentrated. The resulting residue was purified by flash column chromatography (DCM:MeOH, 7:3) to afford **DL-I-102** (40.0 mg, 0.09 mmol, 35%) as a white amorphous solid.

^{1}H NMR (500 MHz, MeOD) δ 7.71 (dd, *J* = 11.6, 2.4 Hz, 1H), 7.37 (t, *J* = 8.5 Hz, 1H), 7.25 (ddd, *J* = 8.8, 2.4, 1.1 Hz, 1H), 4.14 (m, 2H), 3.96 – 3.81 (m, 2H), 3.5 (br s, 3H), 3.55 – 3.36 (m, 6H), 2.95 (dd, *J* = 13.2, 11.4 Hz, 1H), 2.69 – 2.58 (m, 2H), 2.07 (m, 1H), 1.80 (m, 1H), 1.56 – 1.34 (m, 2H), and 1.17 (q, *J* = 12.5 Hz, 1H).

^{13}C NMR (126 MHz, MeOD) δ 174.2, 167.8, 164.4, 161.6 (d, J_{CF} = 244.3 Hz), 158.1, 140.3 (d, J_{CF} = 11.1 Hz), 131.5, 117.3 (d, J_{CF} = 3.9 Hz), 116.1 (d, J_{CF} = 19.1 Hz), 109.3 (d, J_{CF} = 26.7 Hz), 65.3, 56.9, 53.3, 51.3, 49.7, 45.0, 44.6, 39.4, 32.1, and 25.2.

IR (neat): 3323, 2926, 2842, 1733, 1689, 1565, 1489, 1411, 1328, 1269, 1523, and 889 cm^{-1}.

HRMS (ESI): Calcd for $C_{20}H_{27}ClFN_4O_5^+$ [M+H$^+$]: 457.1659; found 457.1652.

[α]$_D^{23}$ -23.18 (c 1.00, MeOH)

Methyl (3*R*,5*S*)-5-((4-chloro-3-fluorophenyl)carbamoyl)-1-(4-methylpiperazine-1-carbonyl)piperidine-3-carboxylate (S7)

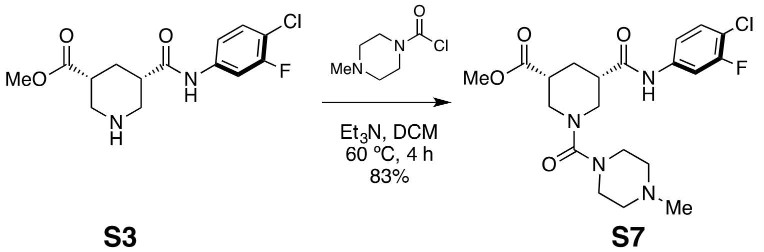

To a solution of **S3** (650 mg, 2.1 mmol) in DCM (0.5 M) under argon atmosphere was added 4-methylpiperazine-1-carbonyl chloride (670 mg, 4.1 mmol) and triethylamine (0.9 mL, 6.2 mmol). The resulting mixture was heated to 60 ºC and stirred for 4 h, then allowed to cool to room temperature and concentrated in vacuo. The crude residue was diluted in DCM (15 mL), saturated aqueous NH_4Cl (15 mL) and the mixture extracted with DCM (3 x 15 mL). The organic layers were combined, washed with brine (15 mL), dried ($MgSO_4$), and concentrated. The resulting residue was purified by flash column chromatography (DCM:MeOH, 9:1) to afford **S7** (756 mg, 1.7 mmol, 83%) as a white amorphous solid.

^{1}H NMR (600 MHz, MeOD) δ 7.67 (dd, *J* = 11.6, 2.4 Hz, 1H), 7.34 (t, *J* = 8.5 Hz, 1H), 7.22 (ddd, *J* = 8.8, 2.4, 1.1 Hz, 1H), 3.93 – 3.83 (m, 1H), 3.82 – 3.73 (m, 1H), 3.66 (s, 3H), 3.30 – 3.23 (m, 2H), 2.91 (ddd, *J* = 26.8, 13.2, 11.4 Hz, 2H), 2.69 – 2.52 (m, 2H), 2.43 (d, *J* = 4.9 Hz, 4H), 2.28 (s, 3H), and 1.82 (q, *J* = 12.6 Hz, 1H).

^{13}C NMR (151 MHz, MeOD) δ 174.4, 173.5, 165.4, 159.9 (d, J_{CF} = 246.2 Hz), 140.3 (d, J_{CF} = 9.6 Hz), 131.5, 117.3 (d, J_{CF} = 3.7 Hz), 116.1 (d, J_{CF} = 17.6 Hz), 109.3 (d, J_{CF} = 26.8 Hz), 55.5, 52.5, 49.7, 49.5, 49.4, 47.41, 46.1, 44.0, 41.5, and 31.6.

IR (neat): 2945, 2923, 2847, 2794, 2365, 1735, 1681, 1609, 1547, 1422, 1293, 1257, 1198, 1140, 1069, 1006 and 868 cm^{-1}.

HRMS (ESI): Calcd for $C_{20}H_{27}ClFN_4O_4^+$ [M+H]$^+$: 441.1705; Found 441.1720.

$[\alpha]_D^{23}$ -12.66 (c 1.00, MeOH)

(*3*S,5*R*)-*N*-(4-chloro-3-fluorophenyl)-5-(hydroxymethyl)-1-(4-methylpiperazine-1-carbonyl)piperidine-3-carboxamide (TFH-II-128)

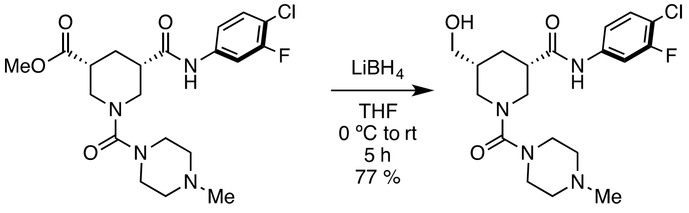

S7 **TFH-II-128**

To a cooled (0 ºC) solution of **S7** (251 mg, 0.57 mmol) in anhydrous THF (0.6 M) under argon atmosphere was added lithium borohydride (2 M in THF, 2.9 mL, 5.7 mmol). The resulting mixture was stirred at 0 ºC for 1 hour, then warm to room temperature and stirred for additional 4 hours. The crude mixture was analyzed by LCMS to confirm the formation of the desired product. Acetic acid (0.1 mL) in hexanes (3 mL) was added to the reaction mixture over 10 min and concentrated. The resulting residue was diluted with Et_2O (15 mL) and saturated aqueous NH_4Cl (10 mL) then extracted with Et_2O (3 x 10 mL). The organic layers were combined, washed with brine (10 mL), dried ($MgSO_4$), and concentrated. The resulting residue was purified by flash column chromatography (DCM:MeOH, 8:2) to afford **TFH-II-128** (181 mg, 0.44 mmol, 77%) as a white amorphous solid.

^{1}H NMR (600 MHz, MeOD) δ 7.72 (dd, *J* = 11.4, 2.2 Hz, 1H), 7.40 (t, *J* = 8.5 Hz, 1H), 7.30 – 7.24 (m, 1H), 3.96 – 3.93 (m, 1H), 3.87 (d, *J* = 12.0 Hz, 1H), 3.81 (m, 2H), 3.61 (m, 2H), 3.43 (dd, *J* = 11.0, 7.5 Hz, 2H), 3.52 – 3.38 (m, 4H), 2.92 (m, 3H), 2.76 (m, 2H), 2.63 (s, 3H), d, 2.62 (t, *J* = 12.4 Hz, 1H), 2.02 (d, *J* = 12.8 Hz, 1H), 1.76 (m, 1H), 1.71 – 1.51 (br s, 2H), and 1.45 (q, *J* = 12.5 Hz, 1H)

^{13}C NMR (151 MHz, MeOD) δ 172.9, 172.7, 163.6, 162.9, 158.5 (d, J_{CF} = 245.2 Hz), 130.1 (d, J_{CF} = 10.1 Hz), 115.9, 107.9 (d, J_{CF} = 29.3 Hz), 131.2, 117.3, 115.9, 109.1 (d, *J* = 26.0 Hz), 64.0, 52.7, 50.6, 44.0, 38.0, 34.4.1 and 30.7.

IR (neat): 2919, 2847, 2360, 2338, 1610, 1538, 1484, 1427, 1252, 1074, and 872 cm^{-1}.

HRMS (ESI): Calcd for $C_{19}H_{27}ClFN_4O_3^+$ [M+H$^+$]: 413.1750; found 413.1744.

$[\alpha]_D^{23}$ -27.80 (c 1.00, MeOH)

(*3*S,5*R*)-5-(Azidomethyl)-*N*-(4-chloro-3-fluorophenyl)-1-(4-methylpiperazine-1-carbonyl)piperidine-3-carboxamide (S8)

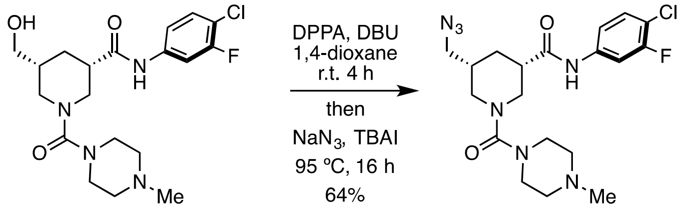

TFH-II-128 **S8**

To a solution of **TFH-II-128** (126 mg, 0.31 mmol) in 1,4-dioxane (1.5 M) under argon atmosphere was added diphenylphosphoryl azide (0.31 mL, 0.61 mmol) and 1,8-diazabicyclo[5.4.0]undec-7-ene (0.14 mL, 6.20 mmol). The reaction mixture was stirred for 4 hours at room temperature. The crude mixture was analyzed by LCMS to confirm the complete consumption of the starting material. To the crude reaction mixture, was added sodium azide (199 mg, 3.06 mmol) and tetrabutylammonium iodide (56 mg, 0.15 mmol). The resulting mixture was heated to reflux and stirred for 16 h, then allowed to cool to room temperature and concentrated in vacuo. The crude residue was diluted in DCM (15 mL), saturated aqueous NH_4Cl (15 mL) and the mixture extracted with DCM (3 x 20 mL). The organic layers were combined, washed with brine (15 mL), dried ($MgSO_4$), and concentrated. The resulting residue was purified by flash column chromatography (DCM:MeOH, 8:2) to afford **S8** (86 mg, 0.20 mmol, 64%) as a white amorphous solid.

^{1}H NMR (600 MHz, MeOD) δ 7.70 (dd, *J* = 11.6, 2.4 Hz, 1H), 7.37 (t, *J* = 8.5 Hz, 1H), 7.30 – 7.24 (m, 1H), 3.91 – 3.80 (m, 2H), 3.34 (m, 4H), 3.53 (m, 2H), 2.90 (m, 1H), 2.65 – 2.55 (m, 2H), 2.45 (br s, 4H), 2.29 (s, 3H), 2.05 (m, 1H), 1.89 – 1.85 (m, 2H), and 1.49 (q, *J* = 12.5 Hz, 1H)

^{13}C NMR (151 MHz, MeOD) δ 173.9, 165.2, 159.9 (d, J_{CF} = 245.4 Hz), 140.4 (d, J_{CF} = 10.4 Hz), 131.5, 131.4, 117.3 (d, J_{CF} = 13.4 Hz), 116.1 (d, J_{CF} = 18.1 Hz), 109.3 (d, J_{CF} = 26.2 Hz), 55.6, 55.3, 51.5, 49.9, 47.5, 47.4, 46.1, 44.6, 36.8, and 32.8.

IR (neat): 2919, 2852, 2798, 2099, 1610, 1493, 1418, 1293, 1249, 1140, 1064, and 863 cm^{-1}.

HRMS (ESI): Calcd for $C_{19}H_{26}ClFN_7O_2^+$ [M+H$^+$]: 438.1821; found 438.1833.

$[\alpha]_D^{23}$ -26.50 (c 1.00, MeOH)

(*3S*,5*S*)-5-(Aminomethyl)-*N*-(4-chloro-3-fluorophenyl)-1-(4-methylpiperazine-1-carbonyl)piperidine-3-carboxamide (ZXC-I-090)

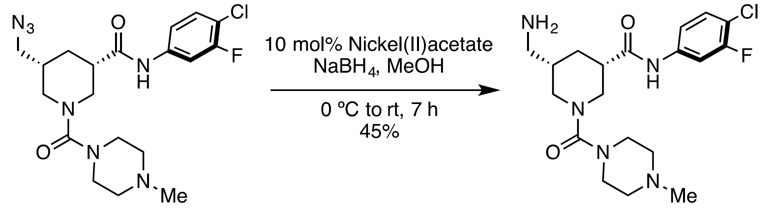

S8 **ZXC-I-090**

To a cooled (0 ºC) solution of **S8** (36 mg, 0.08 mmol) in methanol (2.5 M) under argon atmosphere was added nickel (II) acetate (1.4 mg, 0.008 mmol), sodium borohydride (12 mg, 0.33 mmol). The resulting mixture was stirred at 0 ºC for 1 hour, then warm to room temperature and stirred for additional 6 hours. The crude mixture was analyzed by LCMS to confirm the formation of the desired product. Acetic acid (50 uL) in hexanes (2 mL) was added to the reaction mixture over 5 min and concentrated. The resulting residue was diluted with Et_2O (10 mL) and saturated aqueous NH_4Cl (5 mL) then extracted with Et_2O (3 x 10 mL). The organic layers were combined, washed with brine (20 mL), dried ($MgSO_4$), and concentrated. The resulting residue was purified by flash column chromatography (DCM:MeOH, 8:2) to afford **ZXC-I-090** (15.2 mg, 0.04 mmol, 45%) as a white amorphous solid.

^{1}H NMR (600 MHz, MeOD) δ 7.65 (dd, *J* = 11.3, 2.4 Hz, 1H), 7.30 (t, *J* = 8.4 Hz, 1H), 7.21 (dd, *J* = 8.9, 2.3 Hz, 1H), 3.98 – 3.65 (m, 4H), 3.43 (d, *J* = 12.5 Hz, 2H), 3.14 (s, 1H), 3.02 – 2.90 (m, 1H), 2.86 – 2.79 (m, 2H), 2.71 – 2.55 (m, 2H), 2.09 (d, *J* = 13.1 Hz, 1H), 2.00 – 1.89 (m, 1H), 1.49 (q, *J* = 12.4 Hz, 1H), 1.24 (s, 2H), and 0.92 – 0.79 (m, 1H).

^{13}C NMR (151 MHz, MeOD) δ 174.0, 165.2, 159.9 (d, J_{CF} = 245.2 Hz), 140.4 (d, J_{CF} = 9.2 Hz), 131.5, 117.3 (d, J_{CF} = 3.6 Hz), 116.1 (d, J_{CF} = 18.3 Hz), 109.3 (d, J_{CF} = 26.6 Hz), 55.5, 52.4, 52.2, 51.8, 49.82, 47.5, 46.1, 44.7, 35.5, 35.4, and 33.3.

IR (neat): 2955, 2919, 2847, 2360, 1686, 1610, 1484, 1422, 1247, 1136, 1002, and 863 cm^{-1}.

HRMS (ESI): Calcd for $C_{19}H_{28}ClFN_5O_2^+$ [M+H$^+$]: 412.1916; found 412.1909.

$[\alpha]_D^{23}$ -40.55 (c 1.00, MeOH)

Methyl (3*R*,5*S*)-5-((4-chloro-3-fluorophenyl)carbamoyl)-1-((3*S*,5*R*)-3,4,5-trimethylpiperazine-1-carbonyl)piperidine-3-carboxylate (S9)

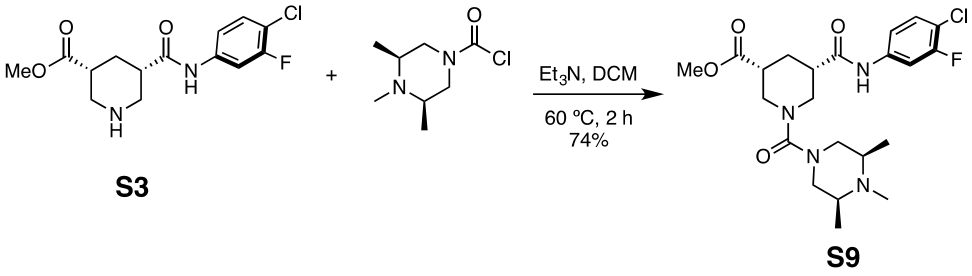

To a solution of **S3** (152 mg, 0.48 mmol) in DCM (0.5 M) under argon atmosphere was added (3R,5S)-3,4,5-trimethylpiperazine-1-carbonyl chloride (460 mg, 2.4 mmol) and triethylamine (1.0 mL, 7.3 mmol). The resulting mixture was heated to 60 ºC and stirred for 2 h, then allowed to cool to room temperature and concentrated in vacuo. The crude residue was diluted in DCM (15 mL), saturated aqueous NH_4Cl (5 mL) and the mixture extracted with DCM (3 x 10 mL). The organic layers were combined, washed with brine (15 mL), dried ($MgSO_4$), and concentrated. The resulting residue was purified by flash column chromatography (DCM:MeOH, 8:2) to afford **S9** (168 mg, 0.36 mmol, 74%) as a white amorphous solid.

^{1}H NMR (600 MHz, MeOD) δ 7.71 (dd, *J* = 11.5, 2.4 Hz, 1H), 7.38 (t, *J* = 8.5 Hz, 1H), 7.25 (ddd, *J* = 8.7, 2.4, 1.1 Hz, 1H), 4.09 – 3.85 (m, 1H), 3.83 – 3.75 (m, 1H), 3.70 (s, 3H), 3.54 (dt, *J* = 13.4, 2.5 Hz, 2H), 2.96 (ddd, *J* = 27.0, 13.2, 11.4 Hz, 2H), 2.73 (ddd, *J* = 13.6, 10.9, 3.0 Hz, 2H), 2.69 – 2.44 (m, 2H), 2.32 (s, 4H), 2.27 – 2.15 (m, 2H), 1.86 (q, *J* = 12.6 Hz, 1H), and 1.11 (d, *J* = 6.1 Hz, 6H)

^{13}C NMR (151 MHz, MeOD) δ 174.4, 173.6, 164.6, 160.0 (d, J_{CF} = 245.6 Hz), 140.3 (d, J_{CF} = 10.9 Hz), 131.6, 117.3 (d, J_{CF} = 2.91 Hz), 116.1 (d, J_{CF} = 16.7 Hz), 109.3 (d, J_{CF} = 24.5 Hz), 59.1, 54.1. 54.0. 52.5. 49.7. 49.6, 49.5. 44.0. 41.5. 37.9. 31.6, 17.5, and 17.4,

IR (neat): 3373, 2989, 2784, 2508, 2074, 1736, 1613, 1493, 1418, 1264, 1201 and 998 cm^{-1}.

HRMS (ESI): Calcd for $C_{22}H_{31}ClFN_4O_4^+$ [M+H]$^+$: 469.2018; Found 469.2009.

[α]$_D^{23}$ -9.23 (c 1.00, MeOH)

(3*S*,5*R*)-*N*-(4-chloro-3-fluorophenyl)-5-(hydroxymethyl)-1-((3*S*,5*R*)-3,4,5-trimethylpiperazine-1-carbonyl)piperidine-3-carboxamide (TFH-II-151)

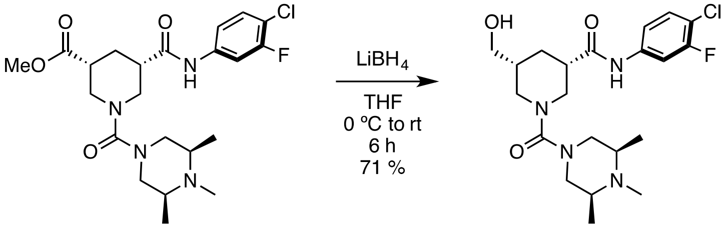

S9 **TFH-II-151**

To a cooled (0 ºC) solution of **S9** (143 mg, 0.31 mmol) in anhydrous THF (0.7 M) under argon atmosphere was added lithium borohydride (2 M in THF, 1.5 mL, 3.1 mmol). The resulting mixture was stirred at 0 ºC for 1 hour, then warm to room temperature and stirred for additional 5 hours. The crude mixture was analyzed by LCMS to confirm the formation of the desired product. Acetic acid (50 uL) in hexanes (1 mL) was added to the reaction mixture over 5 min and concentrated. The resulting residue was diluted with Et_2O (10 mL) and saturated aqueous NH_4Cl (5 mL) then extracted with Et_2O (3 x 10 mL). The organic layers were combined, washed with brine (10 mL), dried ($MgSO_4$), and concentrated. The resulting residue was purified by flash column chromatography (DCM:MeOH, 8:2) to afford **TFH-II-151** (95.5 mg, 0.22 mmol, 71%) as a white amorphous solid.

^{1}H NMR (600 MHz, MeOD) δ 7.73 (dd, *J* = 11.6, 2.4 Hz, 1H), 7.40 (t, *J* = 8.5 Hz, 1H), 7.30 – 7.24 (m, 1H), 3.90 (m, 2H), 3.7 (t, *J* = 13.9 Hz, 2H), 3.53 (dd, *J* = 11.0, 5.1 Hz, 1H), 3.43 (dd, *J* = 11.0, 7.5 Hz, 2H), 3.45 – 3.35 (m, 1H), 3.09 (m, 1H), 3.04 (m, 2H), 2.98 (s, 3H), 2.67 (t, *J* = 12.4 Hz, 1H), 2.65 (m, z1H), 2.03 (d, *J* = 12.9 Hz, 1H), 1.78 (m, 1H), 1.49 (q, *J* = 12.5 Hz, 1H), and 1.41 (s, 6H), and 1.34 (br s, 1H)

^{13}C NMR (151 MHz, MeOD) δ 174.2, 164.0, 159.9 (d, J_{CF} = 246.2 Hz), 140.3 (d, J_{CF} = 9.3 Hz), 131.6, 118.9, 117.3 (d, J_{CF} = 3.7 Hz), 116.0 (d, J_{CF} = 18.2 Hz), 109.3 (d, J_{CF} = 26.3 Hz), 65.3, 61.5, 61.4, 52.1, 51.9, 51.3, 49.6, 44.5, 39.5, 37.4, 32.1, and 14.9.

IR (neat): 3286, 2917, 2851, 2367, 1681, 1611, 1548, 1481, 1423, 1255, 1142, and 862 cm^{-1}.

HRMS (ESI): Calcd for $C_{21}H_{30}ClFN_4NaO_3^+$ [M+Na$^+$]: 463.1888; found 463.1892.

$[\alpha]_D^{23}$ -22.34 (c 1.00, MeOH)

(*3S*,5*R*)-5-(azidomethyl)-*N*-(4-chloro-3-fluorophenyl)-1-((3*S*,5*R*)-3,4,5-trimethylpiperazine-1-carbonyl)piperidine-3-carboxamide (S10)

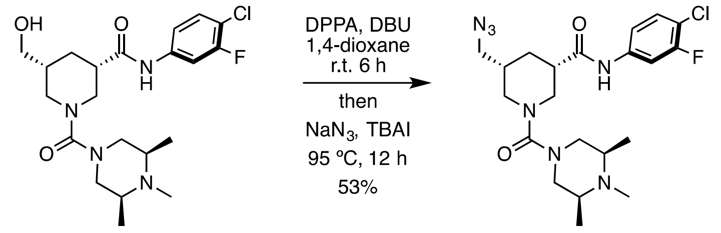

TFH-II-151 **S10**

To a solution of **TFH-II-151** (75 mg, 0.17 mmol) in 1,4-dioxane (2.0 M) under argon atmosphere was added diphenylphosphoryl azide (73 uL, 0.34 mmol) and 1,8-diazabicyclo[5.4.0]undec-7-ene (76 uL, 0.51 mmol). The reaction mixture was stirred for 6 hours at room temperature. The crude mixture was analyzed by LCMS to confirm the complete consumption of the starting material. To the crude reaction mixture, was added sodium azide (110 mg, 1.7 mmol) and tetrabutylammonium iodide (31 mg, 0.09 mmol). The resulting mixture was heated to reflux and stirred for 12 h, then allowed to cool to room temperature and concentrated in vacuo. The crude residue was diluted in DCM (10 mL), saturated aqueous NH_4Cl (5 mL) and the mixture extracted with DCM (3 x 10 mL). The organic layers were combined, washed with brine (15 mL), dried ($MgSO_4$), and concentrated. The resulting residue was purified by flash column chromatography (DCM:MeOH, 8:2) to afford **S10** (39 mg, 0.09 mmol, 53%) as a white amorphous solid.

^{1}H NMR (600 MHz, MeOD) δ 7.71 (dd, *J* = 11.6, 2.4 Hz, 1H), 7.38 (t, *J* = 8.5 Hz, 1H), 7.25 (ddd, *J* = 8.7, 2.4, 1.1 Hz, 1H), 3.88 – 3.71 (m, 2H), 3.63 – 3.51 (m, 2H), 3.35 (dd, *J* = 12.3, 5.7 Hz, 1H), 3.26 (dd, *J* = 12.4, 7.5 Hz, 1H), 2.93 (dd, *J* = 13.1, 11.5 Hz, 1H), 2.80 (ddd, *J* = 13.7, 11.0, 7.6 Hz, 2H), 2.67 – 2.58 (m, 2H), 2.57 – 2.50 (m, 2H), 2.44 (s, 3H), 2.06 (d, *J* = 12.8 Hz, 1H), 1.91 – 1.77 (m, 1H), 1.50 (q, *J* = 12.4 Hz, 1H), and 1.19 (dd, *J* = 6.3, 1.2 Hz, 6H).

^{13}C NMR (151 MHz, MeOD) δ 173.9, 164.5, 159.9 (d, J_{CF} = 244.9 Hz), 140.4 (d, J_{CF} = 10.1 Hz), 131.6, 130.4, 130.3, 121.8, 117.3 (d, J_{CF} = 3.5 Hz), 116.1 (d, J_{CF} = 18.3 Hz), 109.3 (d, J_{CF} = 26.2 Hz), 59.6, 55.4, 53.4, 51.4, 49.7, 44.6, 37.5, 36.8, 32.9, and 16.9.

IR (neat): 3051, 2917, 2847, 2099, 1690, 1606, 1541, 1423, 1206, 1197, 929, and 894 cm^{-1}.

HRMS (ESI): Calcd for $C_{21}H_{30}ClFN_7O_2^+$ [M+H$^+$]: 466.2134; found 466.2135.

$[\alpha]_D^{23}$ -16.63 (c 1.00, MeOH)

(*3*S,5*S*)-5-(aminomethyl)-*N*-(4-chloro-3-fluorophenyl)-1-((3*S*,5*R*)-3,4,5-trimethylpiperazine-1-carbonyl)piperidine-3-carboxamide (ZXC-I-092)

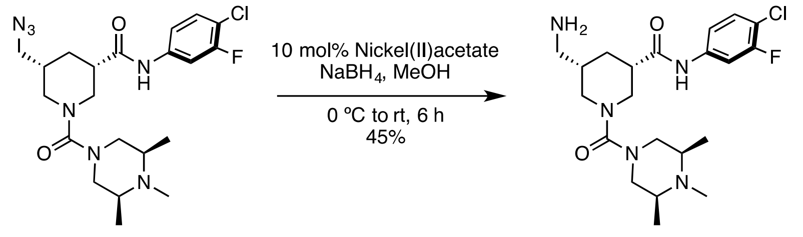

S10 **ZXC-I-092**

To a cooled (0 ºC) solution of **S10** (53 mg, 0.11 mmol) in methanol (2.5 M) under argon atmosphere was added nickel (II) acetate (2.8 mg, 0.011 mmol), sodium borohydride (22 mg, 0.66 mmol). The resulting mixture was stirred at 0 ºC for 1 hour, then warm to room temperature and stirred for additional 5 hours. The crude mixture was analyzed by LCMS to confirm the formation of the desired product. Acetic acid (10 uL) in hexanes (1 mL) was added to the reaction mixture over 5 min and concentrated. The resulting residue was diluted with Et_2O (10 mL) and saturated aqueous NH_4Cl (5 mL) then extracted with Et_2O (3 x 5 mL). The organic layers were combined, washed with brine (10 mL), dried ($MgSO_4$), and concentrated. The resulting residue was purified by flash column chromatography (DCM:MeOH, 7:3) to afford **ZXC-I-092** (22.5 mg, 0.05 mmol, 45%) as a white amorphous solid.

^{1}H NMR (500 MHz, MeOD) δ 7.71 (dd, *J* = 11.5, 2.3 Hz, 1H), 7.38 (t, *J* = 8.5 Hz, 1H), 7.25 (dd, *J* = 8.7, 2.5 Hz, 1H), 3.82 (dd, *J* = 31.5, 14.2 Hz, 4H), 3.38 (br m, 8H), 3.07 (m, 2H), 2.97 (s, 3H), 2.75 – 2.64 (m, 2H), 2.13 (d, *J* = 13.1 Hz, 1H), 2.04 – 1.93 (m, 1H), 1.56 (q, *J* = 12.3 Hz, 1H), and 1.41 (d, *J* = 6.4 Hz, 6H).

^{13}C NMR (126 MHz, MeOD) δ 174.3, 165.2, 164.6, 163.8, 160.0 (d, J_{CF} = 245.0 Hz), 140.42 (d, J_{CF} = 9.8 Hz), 131.54, 131.1, 129.7, 117.3 (d, J_{CF} = 3.5 Hz), 116.1 (d, J_{CF} = 18.2 Hz), 115.2, 109.3 (d, J_{CF} = 25.7 Hz), 65.0, 55.9, 52.7, 47.5, 45.0, 44.9, 38.1, and 33.8.

IR (neat): 2911, 2846, 2846, 2366, 1688, 1609, 1544, 1420, 1236, 1186, 1004, and 877 cm^{-1}.

HRMS (ESI): Calcd for $C_{21}H_{32}ClFN_5O_2^+$ [$M+H^+$]: 440.2223; found 440.2220.

$[\alpha]_D^{23}$ -27.42 (c 1.00, MeOH)

(*S*)-*N*-(4-chloro-3-fluorophenyl)-1-(4-methylpiperazine-1-carbonyl)piperidine-3-carboxamide (TFH-I-070-A6)

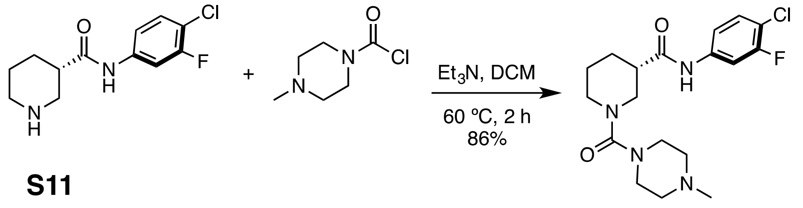

S11

TFH-I-070-A6

To a solution of **S11** (251 mg, 0.98 mmol) in DCM (0.5 M) under argon atmosphere was added 4-methylpiperazine-1-carbonyl chloride (794 mg, 4.9 mmol) and triethylamine (2.7 mL, 19.6 mmol). The resulting mixture was heated to 60 ºC and stirred for 2 h, then allowed to cool to room temperature and concentrated in vacuo. The crude residue was diluted in DCM (20 mL), saturated aqueous NH_4Cl (10 mL) and the mixture extracted with DCM (3 x 20 mL). The organic layers were combined, washed with brine (20 mL), dried ($MgSO_4$), and concentrated. The resulting residue was purified by flash column chromatography (DCM:MeOH, 9:1) to afford **TFH-I-070-A6** (322 mg, 0.84 mmol, 86%) as a white amorphous solid.

^{1}H NMR (600 MHz, MeOD) δ 7.70 (dd, *J* = 11.6, 2.4 Hz, 1H), 7.38 (t, *J* = 8.5 Hz, 1H), 7.26 – 7.24 (m, 1H), 3.82 – 3.78 (m, 3H), 3.71 – 3.61 (m, 1H), 3.56 – 3.44 (m, 2H), 3.22 – 3.11 (m, 6H), 3.07 – 3.04 (m, 1H), 2.92 (s, 3H), 2.61 (dddd, *J* = 10.5, 10.5, 3.8, 3.8 Hz, 1H), 2.10 – 2.06 (m, 1H), 1.84 – 1.79 (m, 2H), and 1.62 – 1.55 (m, 1H).

^{13}C NMR (151 MHz, MeOD) δ 174.5, 164.5, 160.1, 158.2 (d, J_{CF} = 245.2 Hz), 140.4 (d, J_{CF} = 10.0 Hz), 131.6, 117.3 (d, J_{CF} = 3.5 Hz), 116.7 (d, J_{CF} = 18.1 Hz), 116.1, 115.9, 109.3 (d, J_{CF} = 26.1 Hz), 54.1, 45.4, 44.5, 43.7, 40.4, 29.0, and 25.4.

IR (neat): 3277, 3191, 3116, 2938, 2847, 2798, 2362, 2335, 1682, 1605, 1539, 1483, 1290, 1251, 1219, 1998, 1063, and 1009 cm^{-1}.

HRMS (ESI): Calcd for $C_{18}H_{24}ClFN_4NaO_2^+$ [M+H]$^+$: 405.1470; Found 405.1491.

$[\alpha]_D^{23}$ -67.35 (c 1.00, MeOH)

(*S*)-*N*-(4-chloro-3-fluorophenyl)-1-((3*S*,5*R*)-3,4,5-trimethylpiperazine-1-carbonyl)piperidine-3-carboxamide (TFH-I-116-D1)

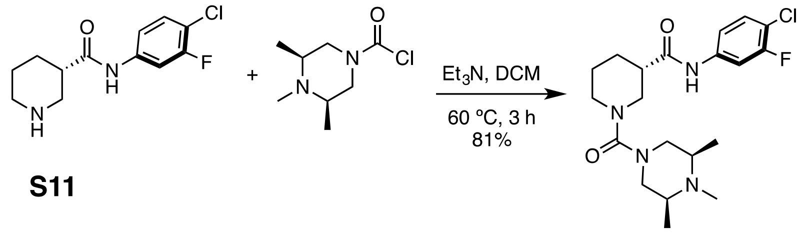

To a solution of **S11** (198 mg, 0.77 mmol) in DCM (0.5 M) under argon atmosphere was added (*3*R,5*S*)-3,4,5-trimethylpiperazine-1-carbonyl chloride (734 mg, 3.9 mmol) and triethylamine (2.2 mL, 15.5 mmol). The resulting mixture was heated to 60 ºC and stirred for 3 h, then allowed to cool to room temperature and concentrated in vacuo. The crude residue was diluted in DCM (20 mL), saturated aqueous NH_4Cl (10 mL) and the mixture extracted with DCM k(3 x 20 mL). The organic layers were combined, washed with brine (20 mL), dried ($MgSO_4$), and concentrated. The resulting residue was purified by flash column chromatography (DCM:MeOH, 9:1) to afford **TFH-I-116-D1** (257 mg, 0.63 mmol, 81%) as an ivory amorphous solid.

^{1}H NMR (600 MHz, MeOD) δ 7.71 (dd, *J* = 11.6, 2.3 Hz, 1H), 7.37 (t, *J* = 8.5 Hz, 1H), 7.32 – 7.23 (m, 1H), 3.85 – 3.74 (m, 3H), 3.65 (d, *J* = 13.1 Hz, 1H), 3.46 – 3.34 (m, 2H), 3.21 (dd, *J* = 13.2, 10.1 Hz, 1H), 3.11 – 3.06 (m, 2H), 3.02 (dd, *J* = 14.9, 11.7 Hz, 1H), 2.96 (s, 3H), 2.59 (dddd, *J* = 10.1, 10.1, 3.8, 3.8 Hz, 1H), 2.10 – 2.02 (m, 1H), 1.86 – 1.78 (m, 2H), 1.64 – 1.52 (m, 1H), and 1.41 (d, *J* = 6.4 Hz, 6H).

^{13}C NMR (151 MHz, MeOD) δ 174.5, 164.1, 159.9 (d, J_{CF} = 245.0 Hz), 140.4 (d, J_{CF} = 9.9 Hz), 131.6, 117.3 (d, J_{CF} = 3.2 Hz), 116.2, 116.1 (d, J_{CF} = 17.9 Hz), 109.3 (d, J_{CF} = 25.9 Hz), 61.5, 54.8, 52.1, 51.9, 49.6, 48.3, 44.4, 37.4, 29.0, 25.3, and 14.9.

IR (neat): 2922, 2855, 2784, 1690, 1640, 1606, 1543, 1477, 1423, 1322, 1260, and 875 cm^{-1}.

HRMS (ESI): Calcd for $C_{20}H_{29}ClFN_4O_2^+$ [M+H]$^+$: 411.1963; Found 411.1944.

$[\alpha]_D^{23}$ -26.16 (c 1.00, MeOH)

Copies of ^{1}H, ^{13}C NMR spectra

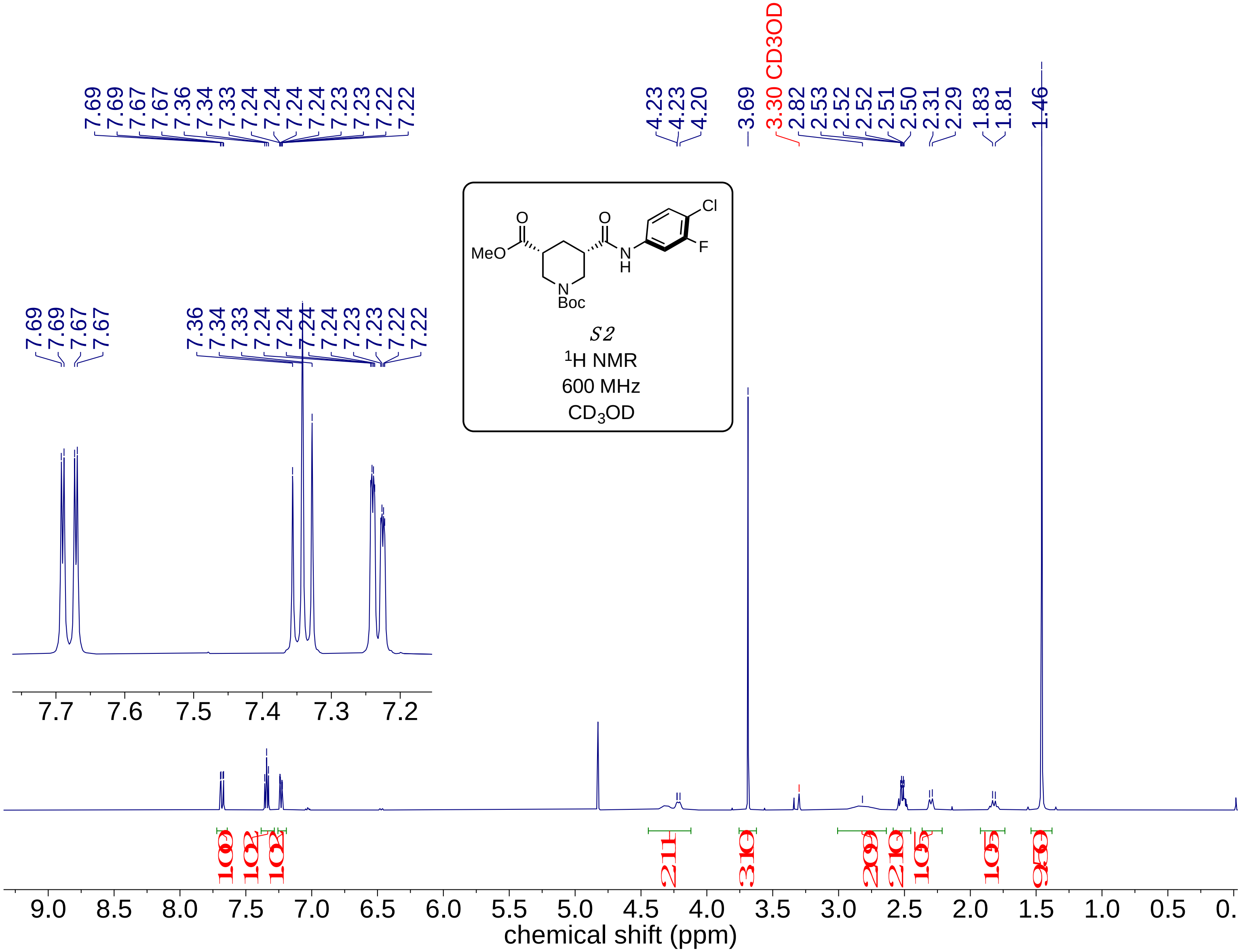

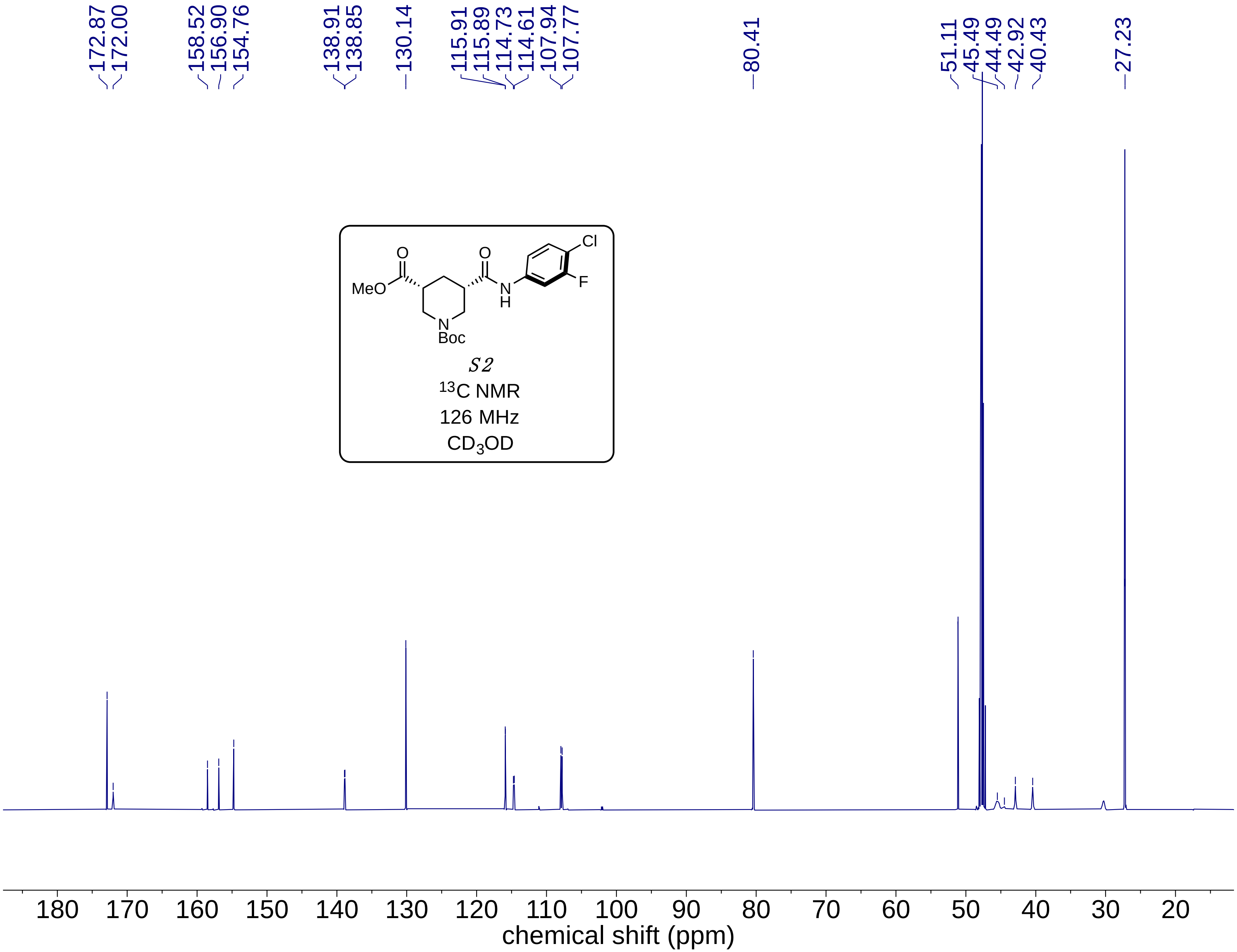
172.87
172.00
158.52
156.90
154.76
138.91
138.85
130.14
115.91
115.89
114.73
114.61
107.94
107.77
80.41
51.11
45.49
44.49
42.92
40.43
27.23
MeO
O
O
N
H
Cl
F
N
Boc
S2
^{13}C NMR
126 MHz
CD_3OD
180 170 160 150 140 130 120 110 100 90 80 70 60 50 40 30 20
chemical shift (ppm)

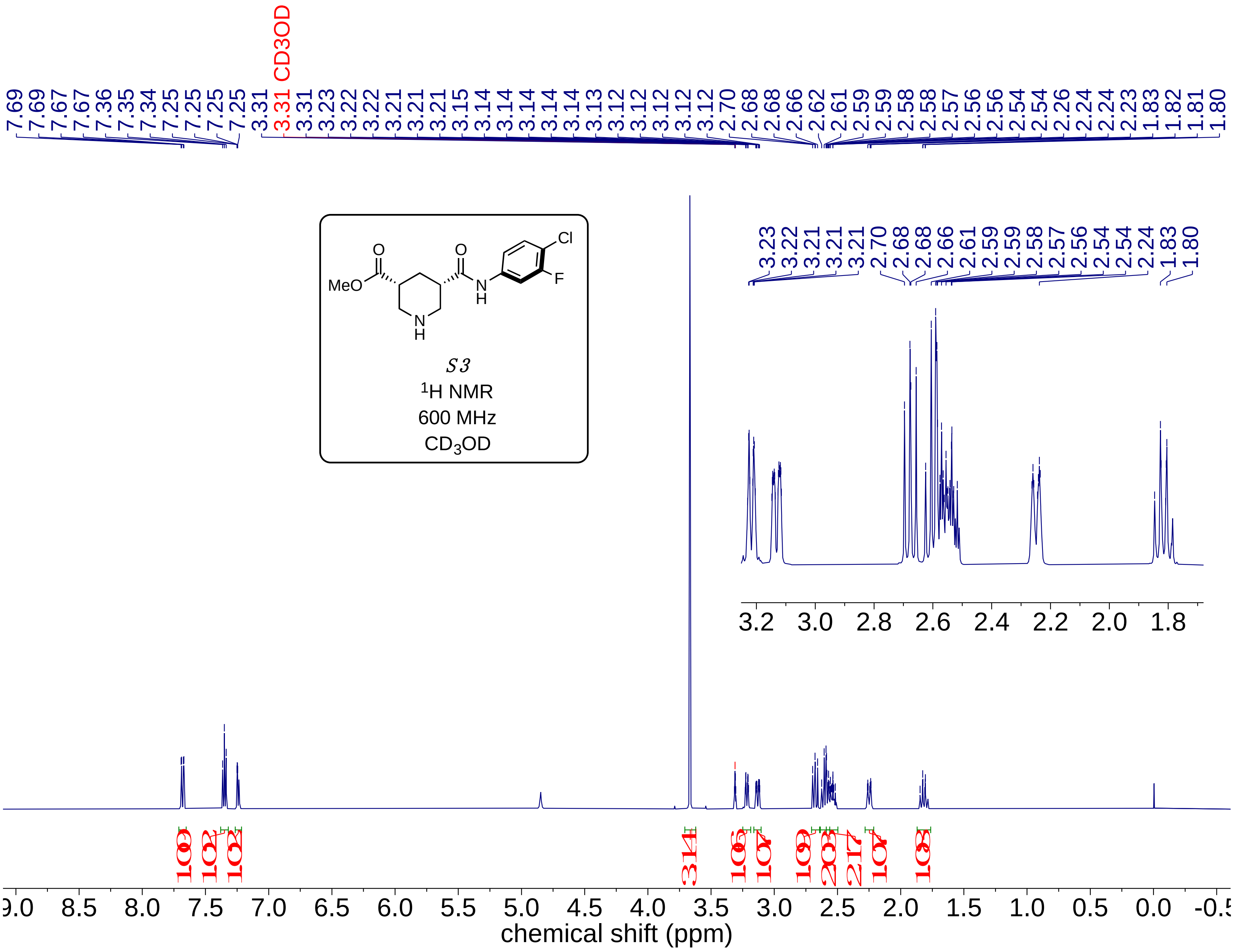

S3
^{1}H NMR
600 MHz
CD_3OD
CD3OD
chemical shift (ppm)

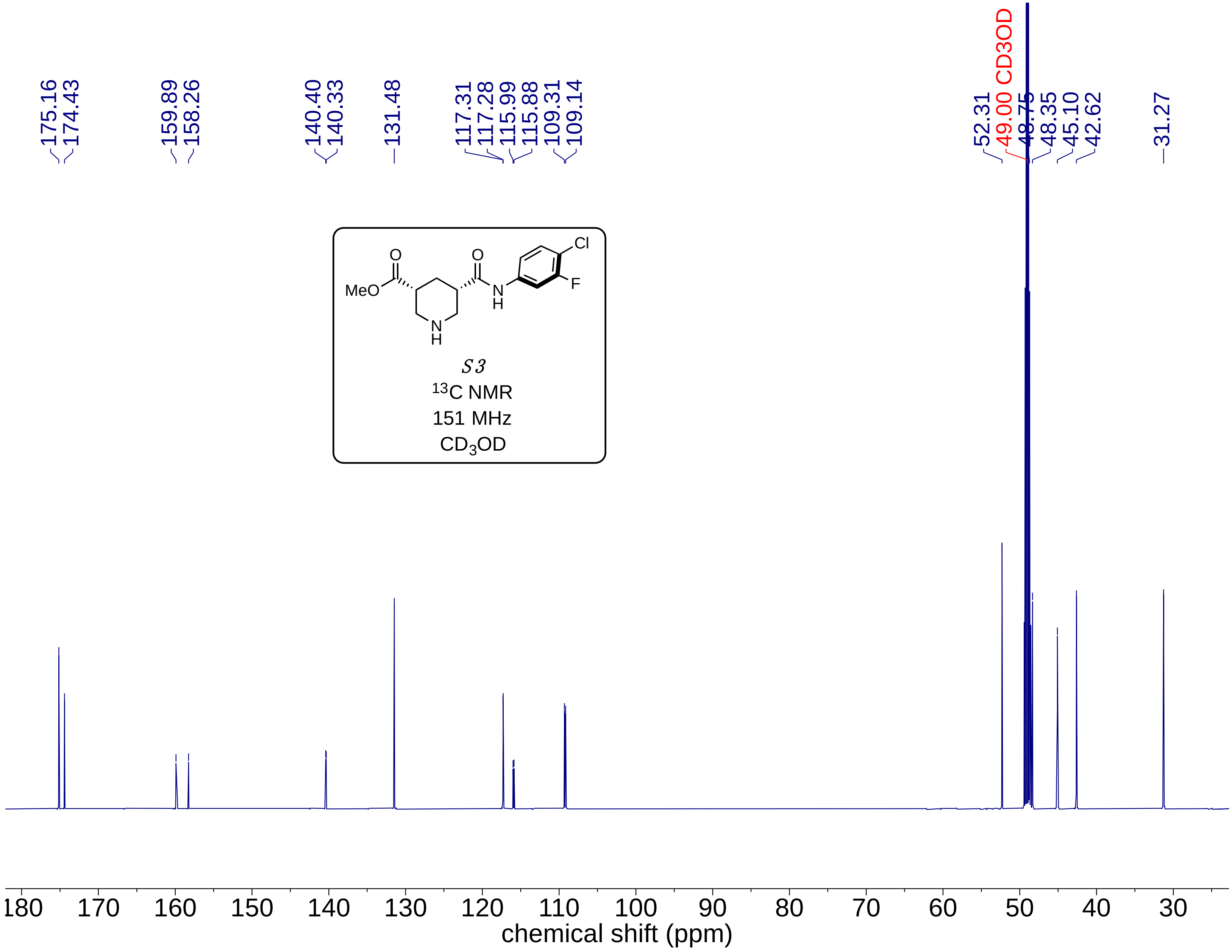

175.16
174.43
159.89
158.26
140.40
140.33
131.48
117.31
117.28
115.99
115.88
109.31
109.14
52.31
49.00 CD3OD
48.75
48.35
45.10
42.62
31.27
MeO
O
O
N
H
N
H
Cl
F
S3
^{13}C NMR
151 MHz
CD_3OD
180
170
160
150
140
130
120
110
100
90
80
70
60
50
40
30
chemical shift (ppm)

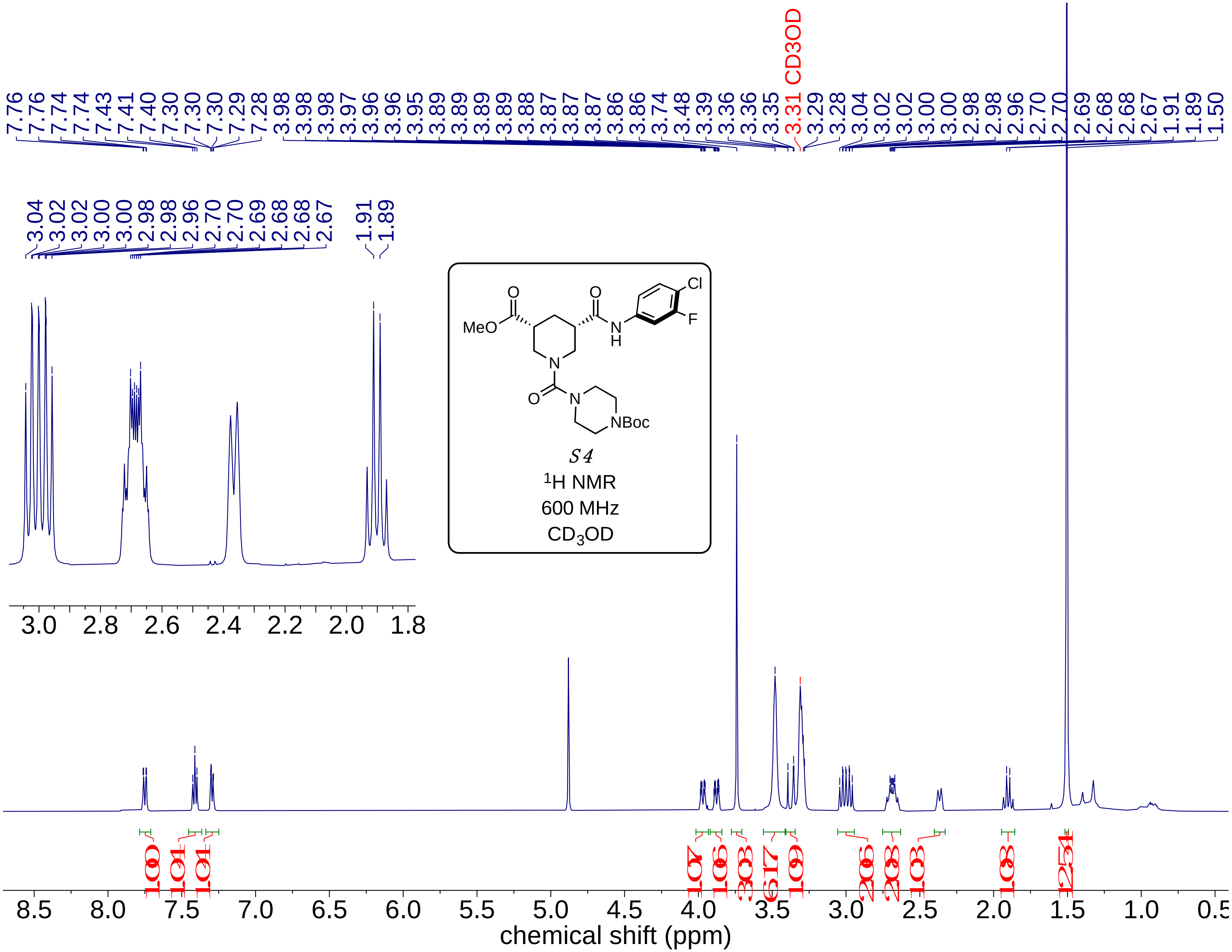

7.76 7.76 7.74 7.74 7.43 7.41 7.40 7.30 7.30 7.30 7.29 7.28 3.98 3.98 3.98 3.97 3.96 3.96 3.95 3.89 3.89 3.89 3.89 3.88 3.87 3.87 3.87 3.86 3.86 3.74 3.48 3.39 3.36 3.36 3.35 3.31 CD3OD 3.29 3.28 3.04 3.02 3.02 3.00 3.00 2.98 2.98 2.96 2.70 2.70 2.69 2.68 2.68 2.67 1.91 1.89 1.50
3.04 3.02 3.02 3.00 3.00 2.98 2.98 2.96 2.70 2.70 2.69 2.68 2.68 2.67 1.91 1.89
3.0 2.8 2.6 2.4 2.2 2.0 1.8
MeO
O
N
H
Cl
F
NBoc
S4
¹H NMR
600 MHz
CD₃OD
8.5 8.0 7.5 7.0 6.5 6.0 5.5 5.0 4.5 4.0 3.5 3.0 2.5 2.0 1.5 1.0 0.5
chemical shift (ppm)

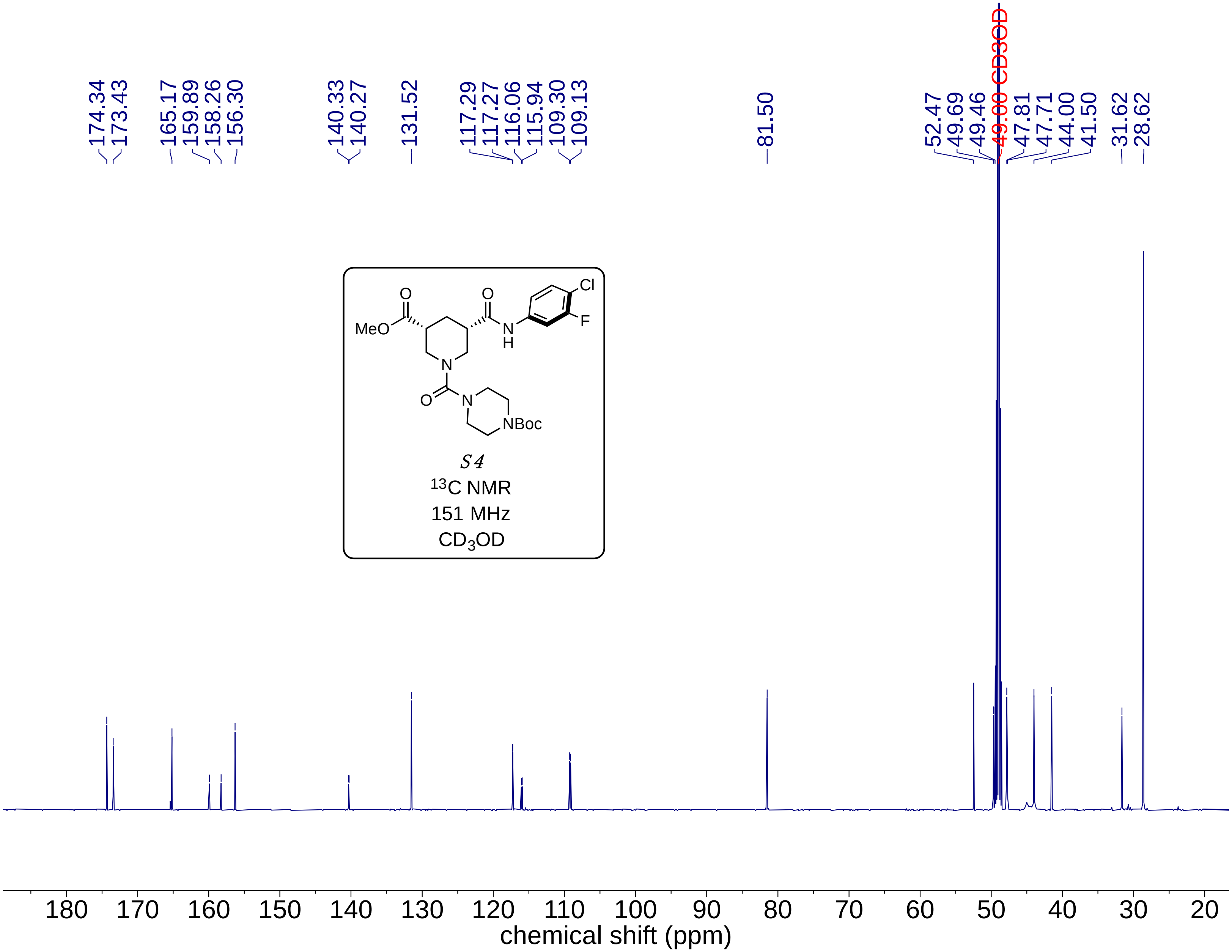

174.34
173.43
165.17
159.89
158.26
156.30
140.33
140.27
131.52
117.29
117.27
116.06
115.94
109.30
109.13
81.50
52.47
49.69
49.46
49.00 CD3OD
47.81
47.71
44.00
41.50
31.62
28.62
O
MeO
O
N
H
Cl
F
N
O
N
NBoc
S4
13C NMR
151 MHz
CD3OD
180 170 160 150 140 130 120 110 100 90 80 70 60 50 40 30 20
chemical shift (ppm)

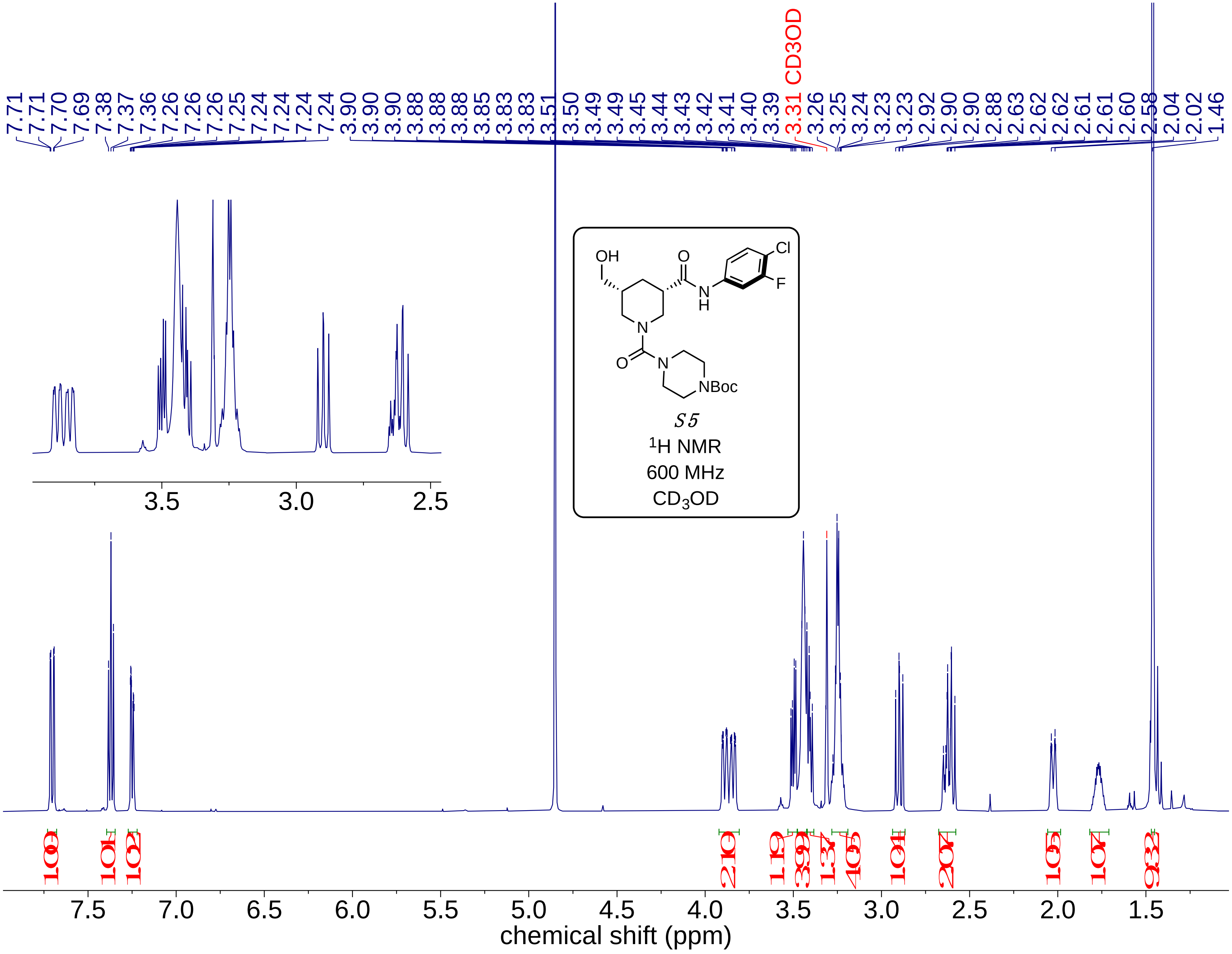
7.71 7.71 7.70 7.69 7.38 7.37 7.36 7.26 7.26 7.26 7.25 7.24 7.24 7.24 7.24 3.90 3.90 3.90 3.88 3.88 3.88 3.85 3.83 3.83 3.51 3.50 3.49 3.49 3.45 3.44 3.43 3.42 3.41 3.40 3.39 3.31 3.26 3.25 3.24 3.23 3.23 2.92 2.90 2.90 2.88 2.63 2.62 2.62 2.61 2.61 2.60 2.58 2.04 2.02 1.46
CD3OD
OH
O
Cl
F
N
H
N
O
N
NBoc
S5
1H NMR
600 MHz
CD3OD
chemical shift (ppm)

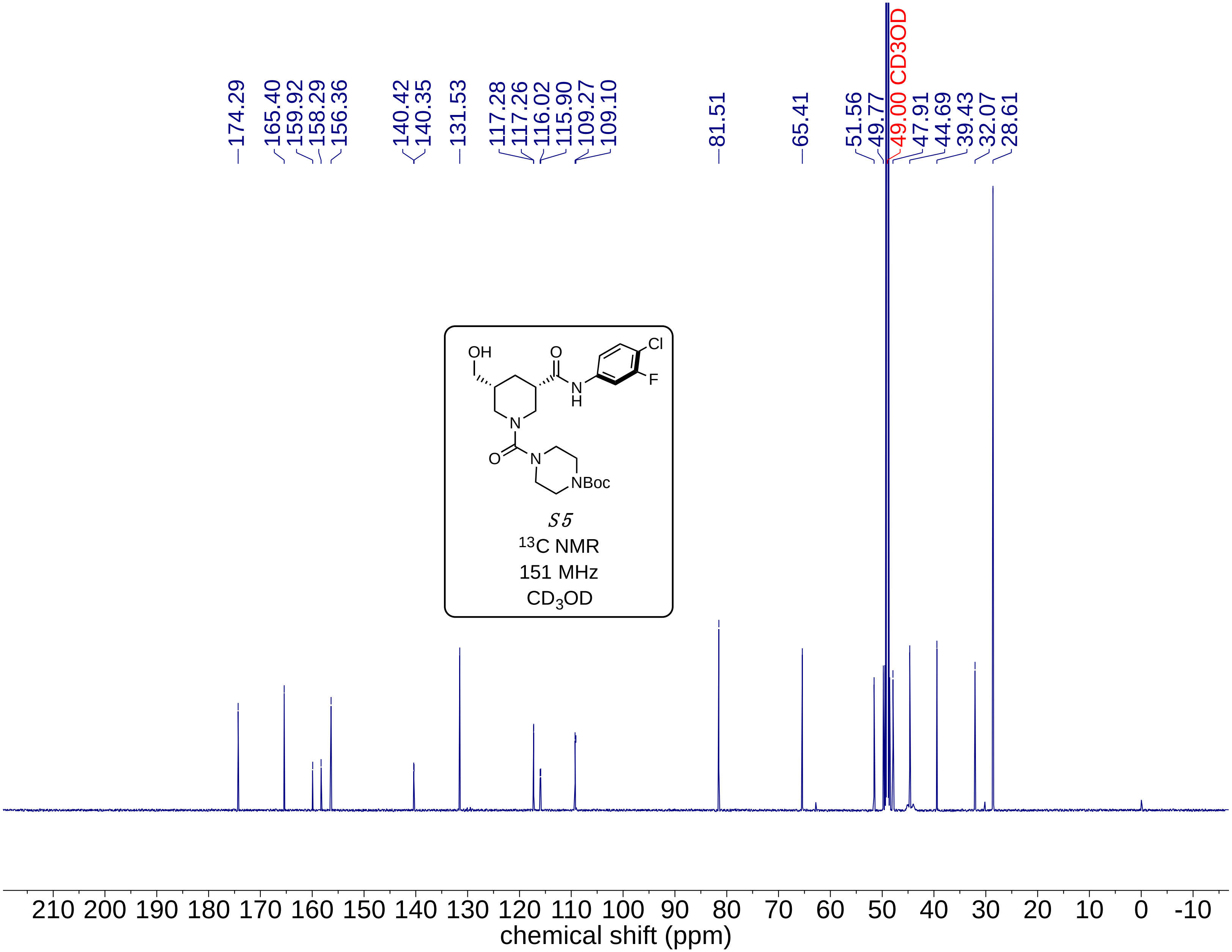
174.29
165.40
159.92
158.29
156.36
140.42
140.35
131.53
117.28
117.26
116.02
115.90
109.27
109.10
81.51
65.41
51.56
49.77
49.00 CD3OD
47.91
44.69
39.43
32.07
28.61
OH
O
Cl
F
N
H
N
O
N
NBoc
S5
13C NMR
151 MHz
CD3OD
210 200 190 180 170 160 150 140 130 120 110 100 90 80 70 60 50 40 30 20 10 0 -10
chemical shift (ppm)

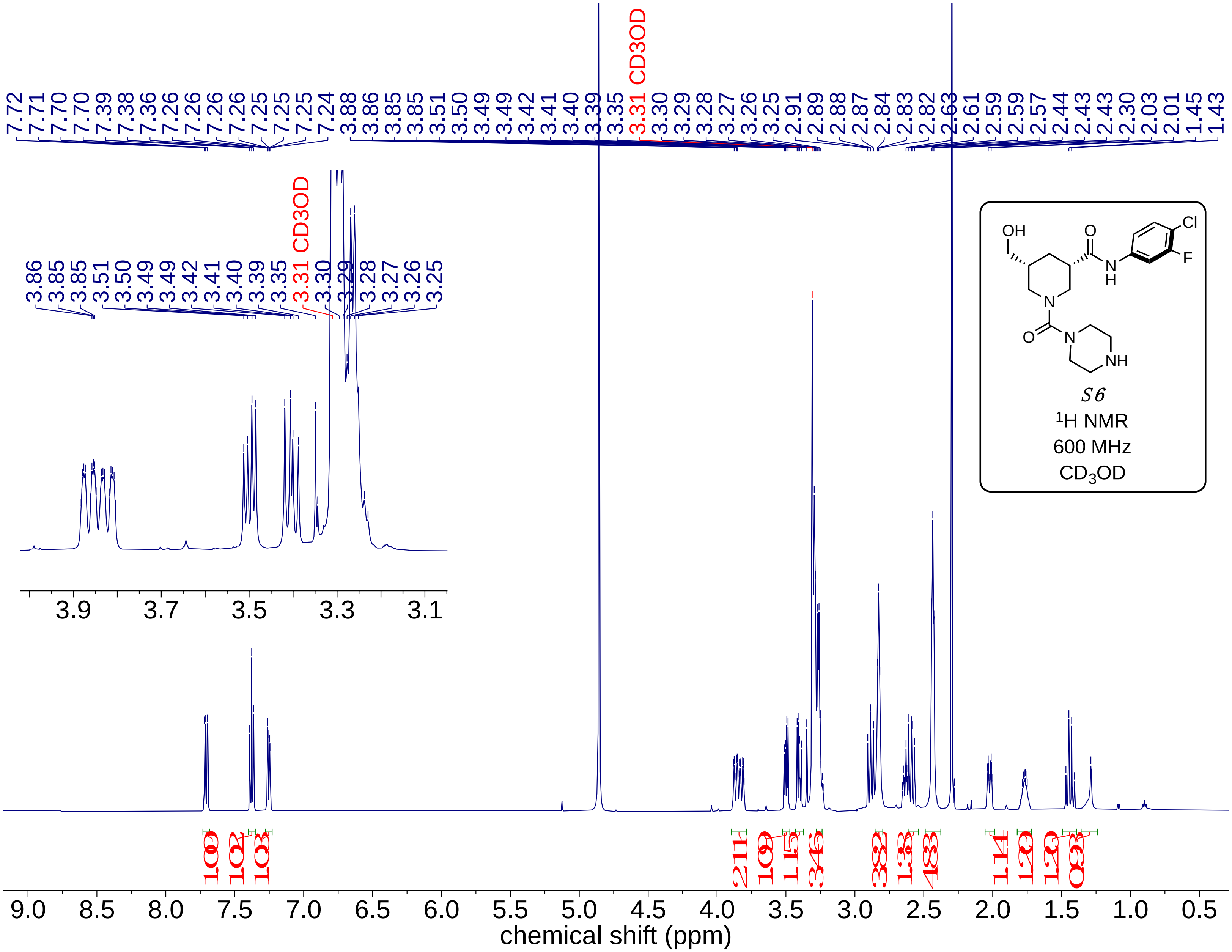
S6
1H NMR
600 MHz
CD3OD
CD3OD
chemical shift (ppm)

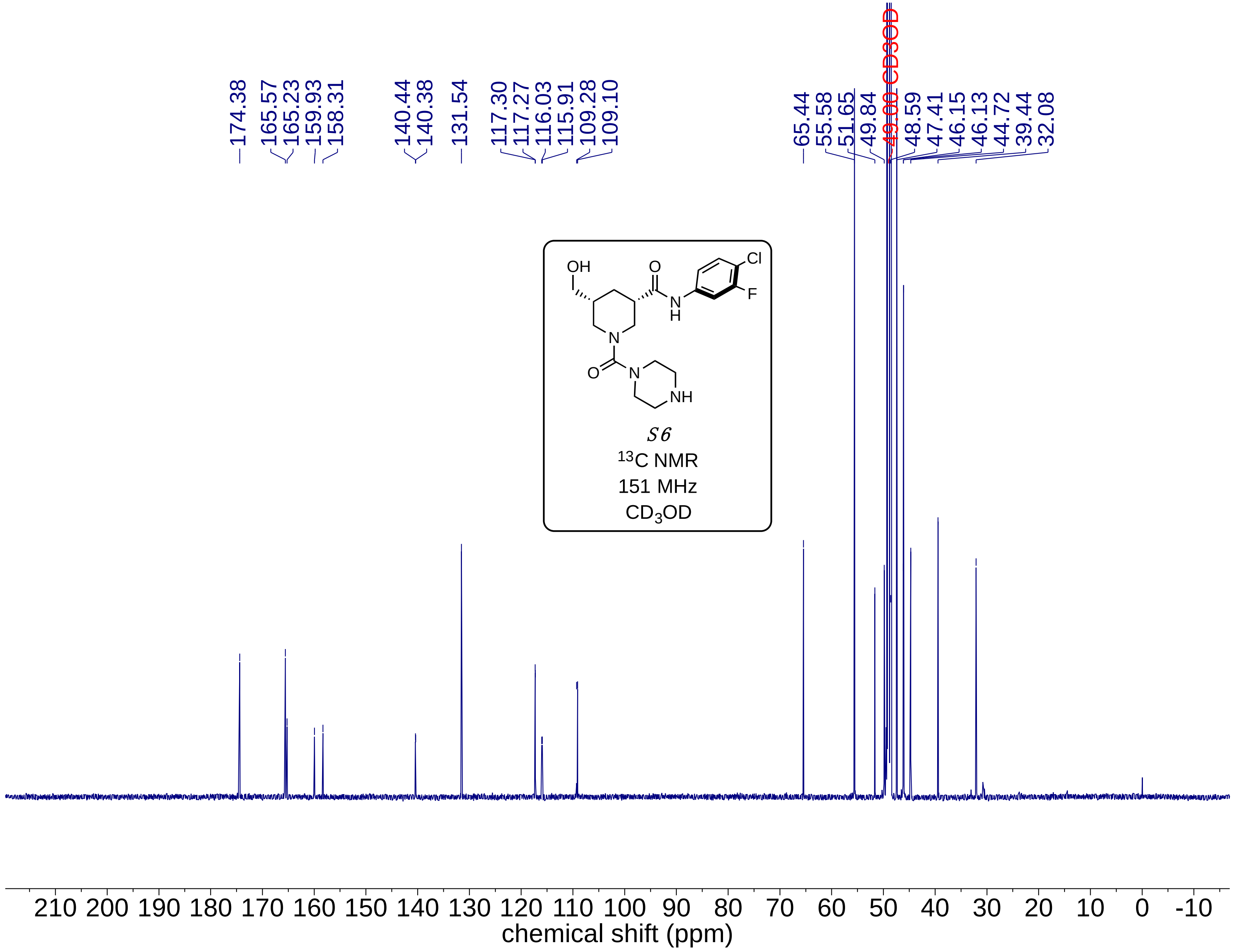
174.38
165.57
165.23
159.93
158.31
140.44
140.38
131.54
117.30
117.27
116.03
115.91
109.28
109.10
65.44
55.58
51.65
49.84
49.00 CD3OD
48.59
47.41
46.15
46.13
44.72
39.44
32.08
OH
O
Cl
N
H
F
N
O
N
NH
S6
13C NMR
151 MHz
CD3OD
210 200 190 180 170 160 150 140 130 120 110 100 90 80 70 60 50 40 30 20 10 0 -10
chemical shift (ppm)

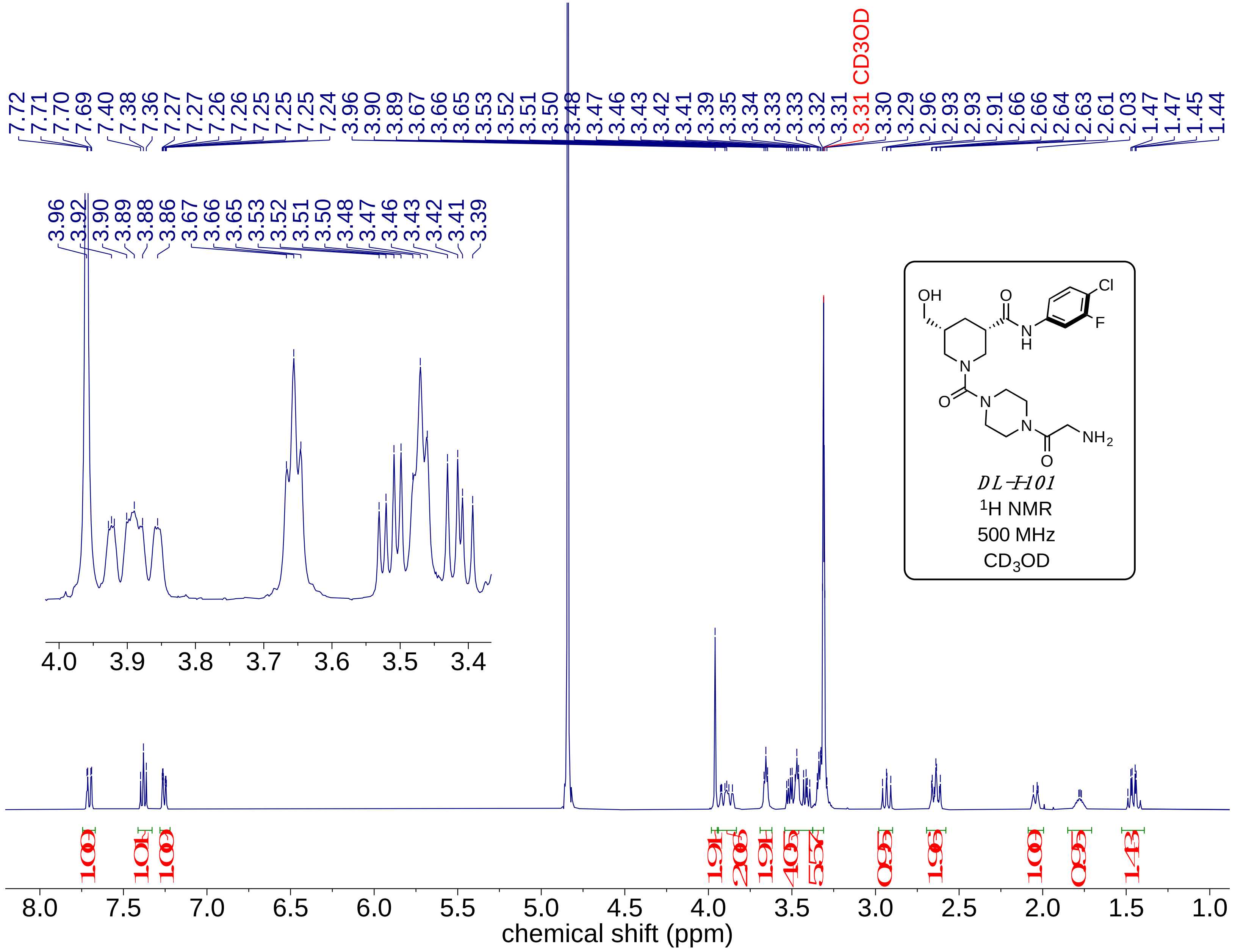

DL-I-101
1H NMR
500 MHz
CD3OD
CD3OD
chemical shift (ppm)

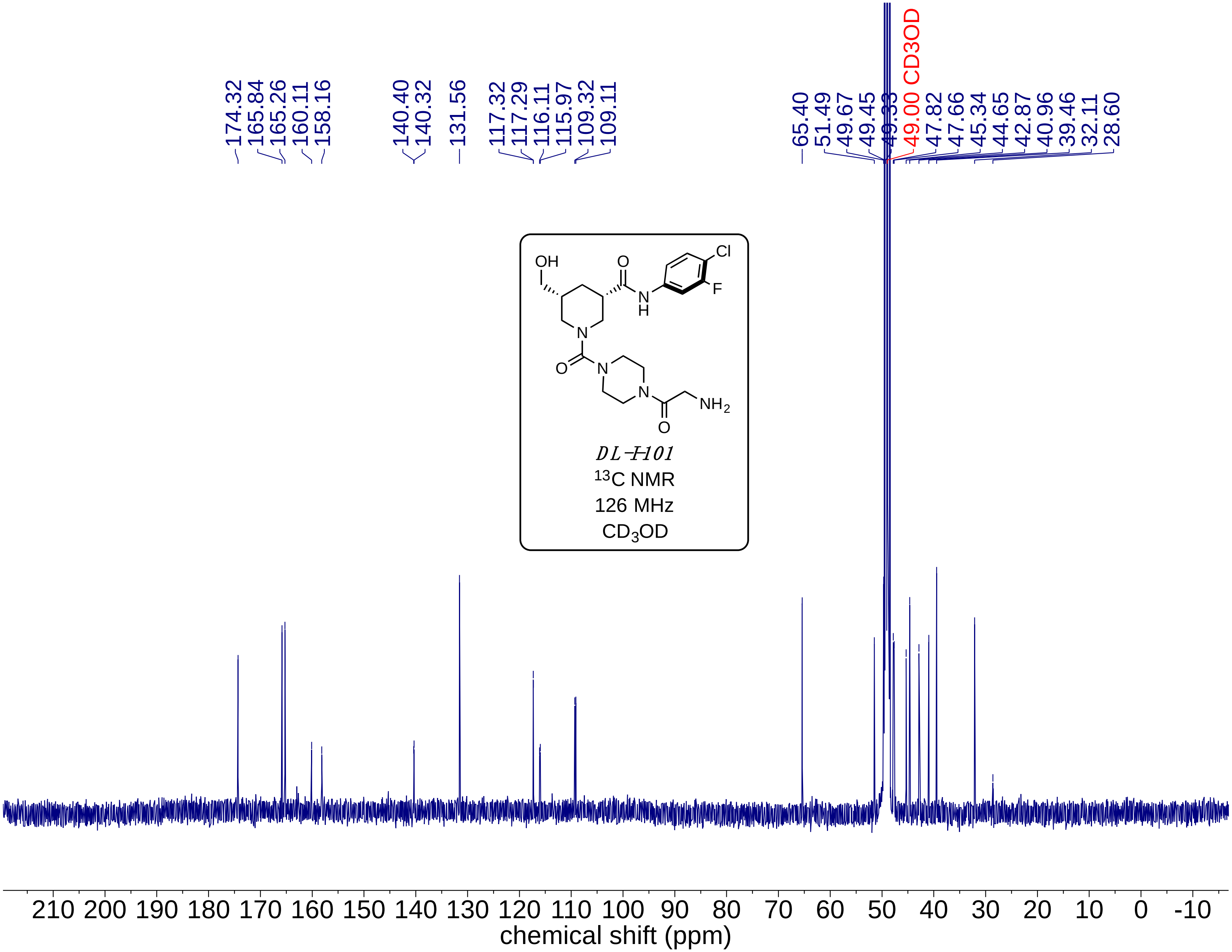
174.32
165.84
165.26
160.11
158.16
140.40
140.32
131.56
117.32
117.29
116.11
115.97
109.32
109.11
65.40
51.49
49.67
49.45
49.33
49.00 CD3OD
47.82
47.66
45.34
44.65
42.87
40.96
39.46
32.11
28.60
OH
O
Cl
F
N
H
N
O
N
N
NH2
O
DL-I-101
13C NMR
126 MHz
CD3OD
210 200 190 180 170 160 150 140 130 120 110 100 90 80 70 60 50 40 30 20 10 0 -10
chemical shift (ppm)

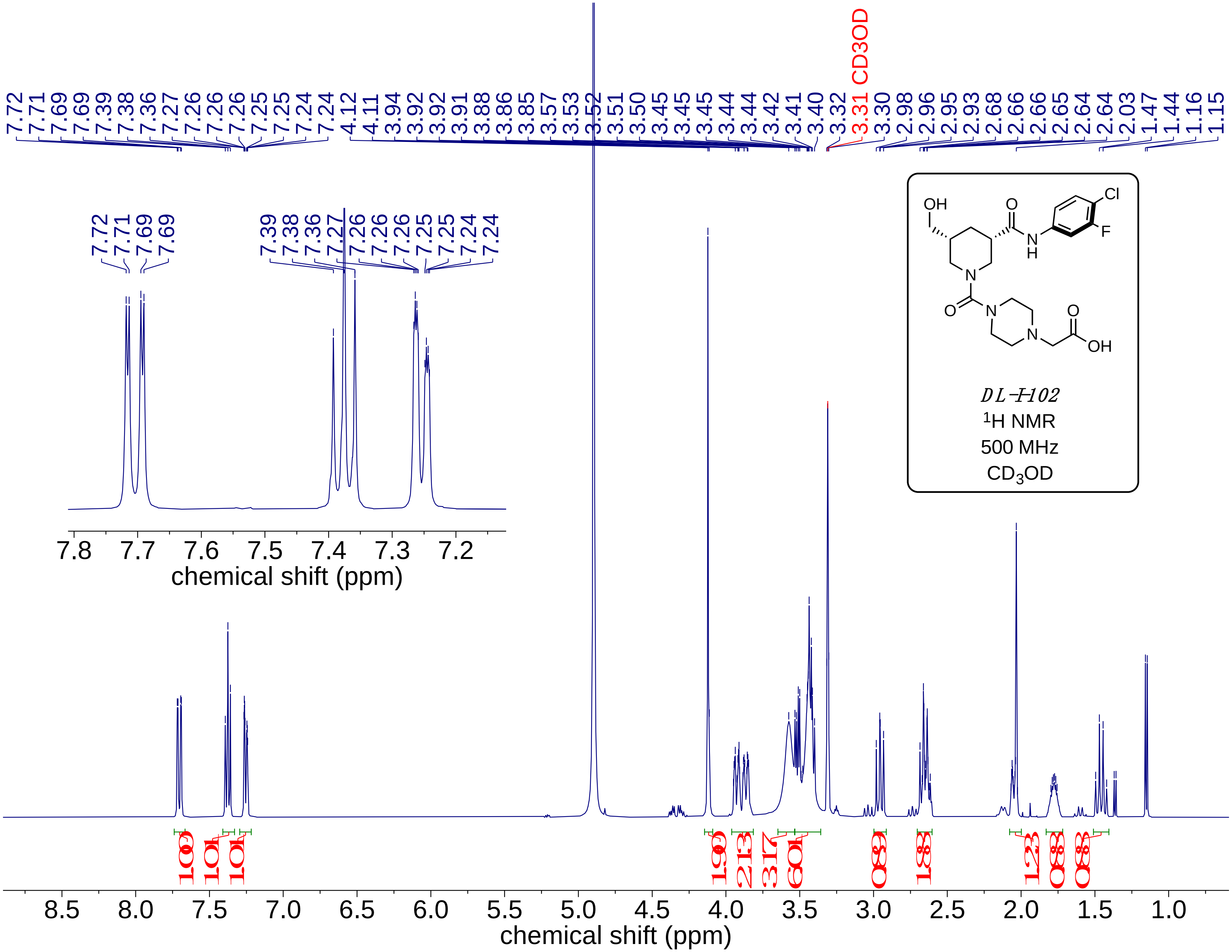

7.72 7.71 7.69 7.69 7.39 7.38 7.36 7.27 7.26 7.26 7.26 7.25 7.25 7.24 7.24 4.12 4.11 3.94 3.92 3.92 3.91 3.88 3.86 3.85 3.57 3.53 3.52 3.51 3.50 3.45 3.45 3.45 3.44 3.44 3.42 3.41 3.40 3.32 3.31 CD3OD 3.30 2.98 2.96 2.95 2.93 2.68 2.66 2.66 2.65 2.64 2.64 2.03 1.47 1.44 1.16 1.15
7.72 7.71 7.69 7.69 7.39 7.38 7.36 7.27 7.26 7.26 7.26 7.25 7.25 7.24 7.24
7.8 7.7 7.6 7.5 7.4 7.3 7.2
chemical shift (ppm)
OH
O
Cl
N
H
F
N
O
N
N
O
OH
^{1}H NMR
500 MHz
CD_3OD
8.5 8.0 7.5 7.0 6.5 6.0 5.5 5.0 4.5 4.0 3.5 3.0 2.5 2.0 1.5 1.0
chemical shift (ppm)

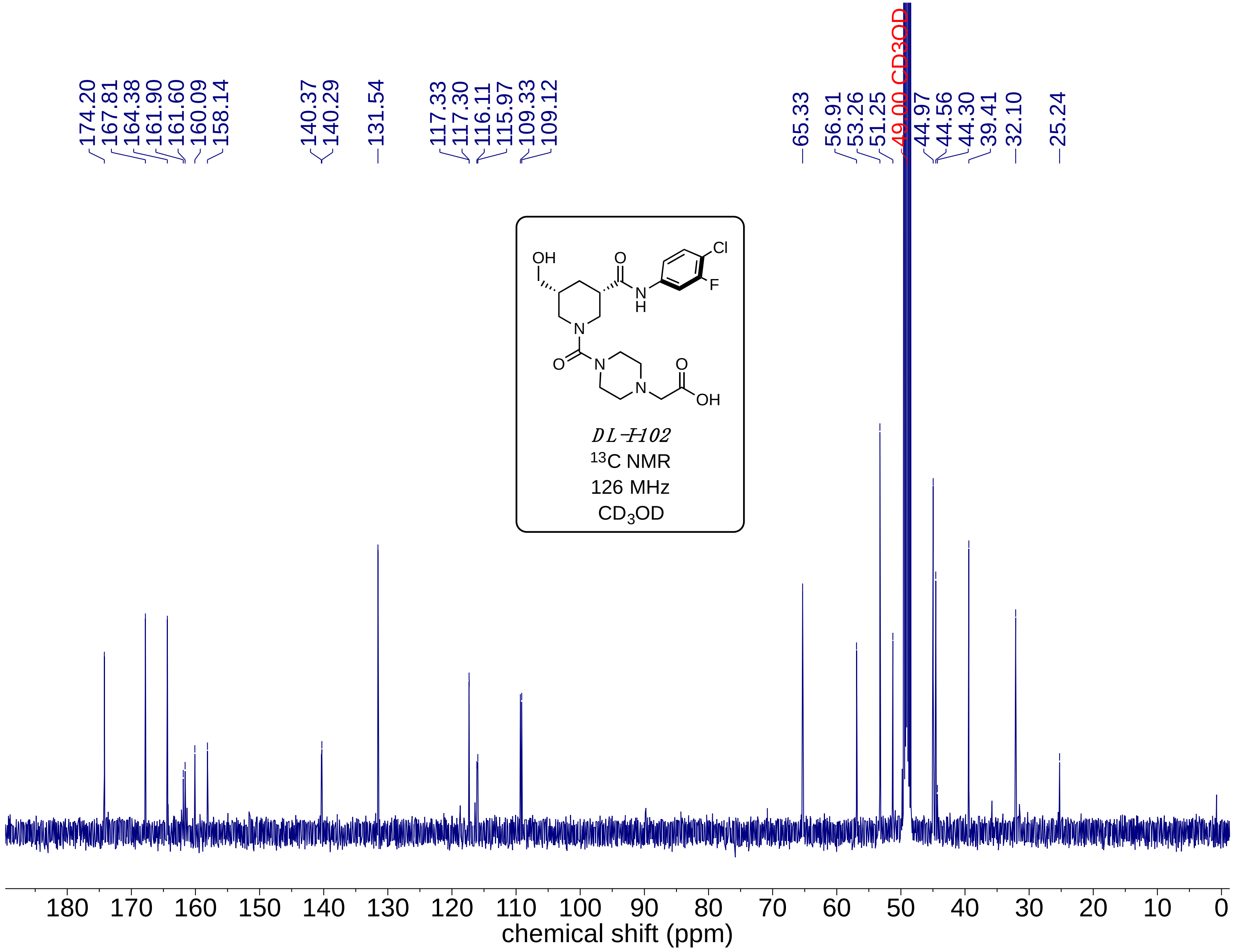

174.20
167.81
164.38
161.90
161.60
160.09
158.14
140.37
140.29
131.54
117.33
117.30
116.11
115.97
109.33
109.12
65.33
56.91
53.26
51.25
49.00 CD3OD
44.97
44.56
44.30
39.41
32.10
25.24
OH
O
Cl
F
N
H
N
O
N
N
O
OH
DL-H102
^{13}C NMR
126 MHz
CD_3OD
180 170 160 150 140 130 120 110 100 90 80 70 60 50 40 30 20 10 0
chemical shift (ppm)

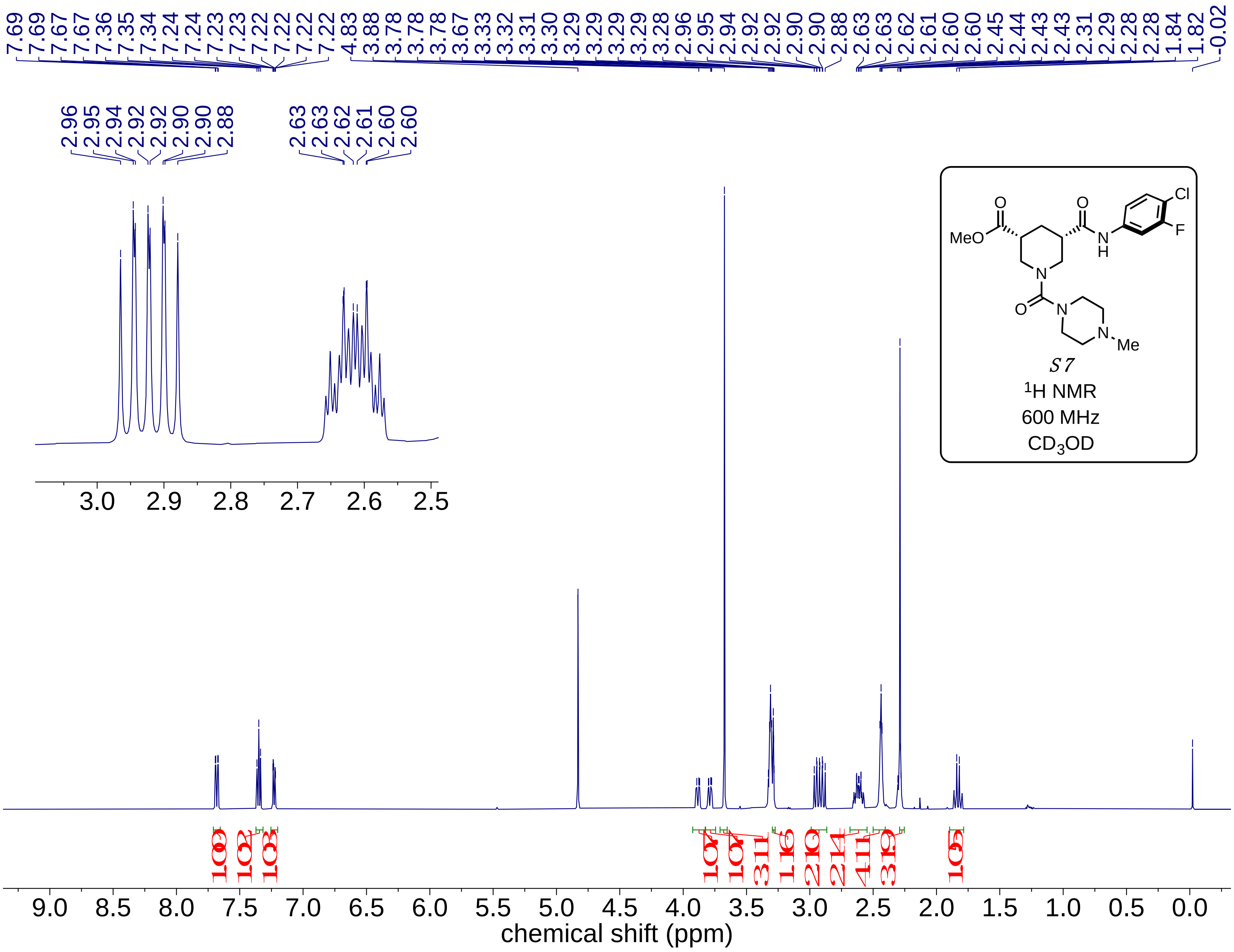

7.69 7.69 7.67 7.67 7.36 7.35 7.34 7.24 7.24 7.23 7.23 7.22 7.22 7.22 7.22 4.83 3.88 3.78 3.78 3.78 3.67 3.33 3.32 3.31 3.30 3.29 3.29 3.29 3.29 3.28 2.96 2.95 2.94 2.92 2.92 2.90 2.90 2.88 2.63 2.63 2.62 2.61 2.60 2.60 2.45 2.44 2.43 2.43 2.31 2.29 2.28 2.28 1.84 1.82 -0.02
2.96 2.95 2.94 2.92 2.92 2.90 2.90 2.88
2.63 2.63 2.62 2.61 2.60 2.60
3.0 2.9 2.8 2.7 2.6 2.5
MeO
O
O
N
H
Cl
F
N
O
N
N
Me
S7
¹H NMR
600 MHz
CD₃OD
9.0 8.5 8.0 7.5 7.0 6.5 6.0 5.5 5.0 4.5 4.0 3.5 3.0 2.5 2.0 1.5 1.0 0.5 0.0
chemical shift (ppm)

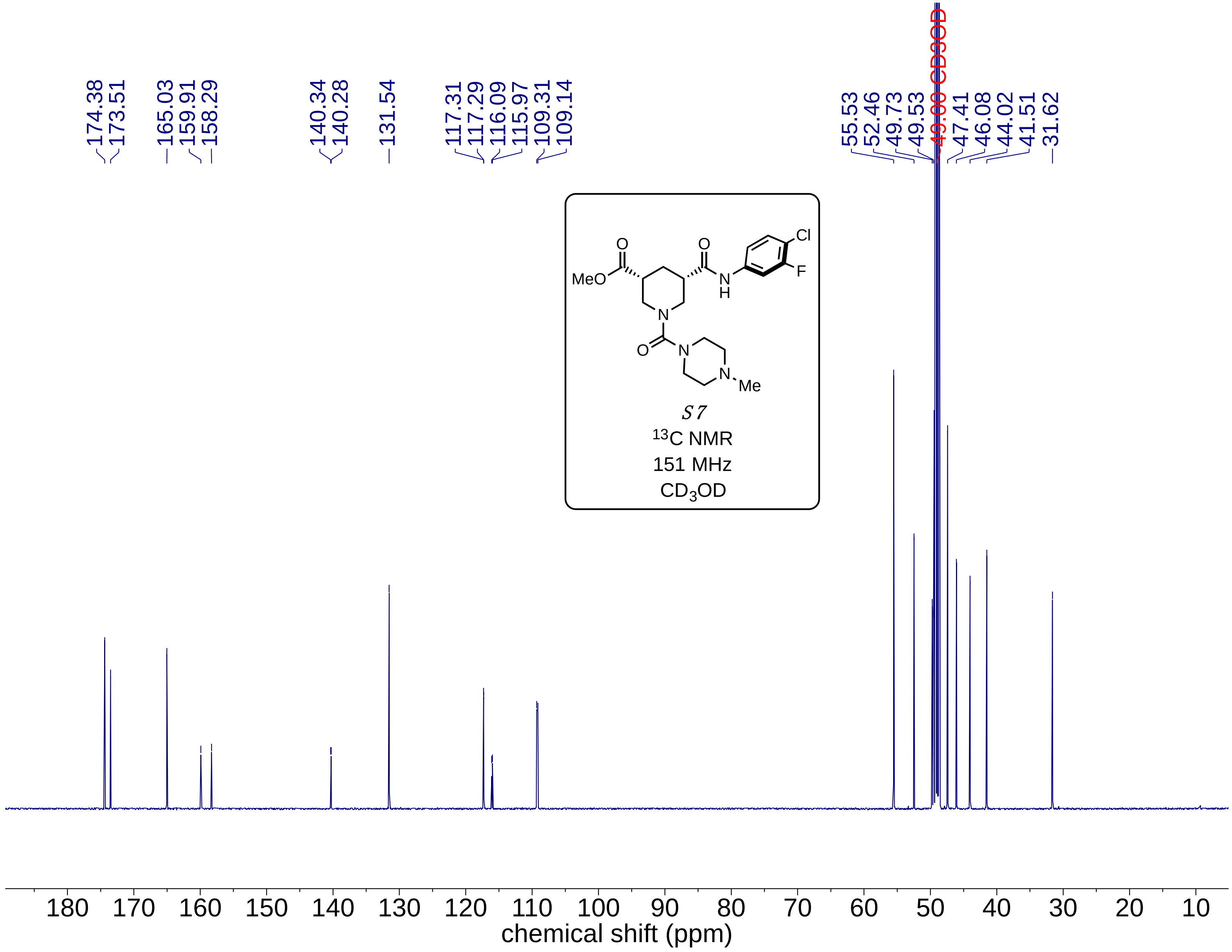
174.38
173.51
165.03
159.91
158.29
140.34
140.28
131.54
117.31
117.29
116.09
115.97
109.31
109.14
55.53
52.46
49.73
49.53
49.00 CD3OD
47.41
46.08
44.02
41.51
31.62
MeO
O
N
H
Cl
F
Me
S7
13C NMR
151 MHz
CD3OD
180
170
160
150
140
130
120
110
100
90
80
70
60
50
40
30
20
10
chemical shift (ppm)

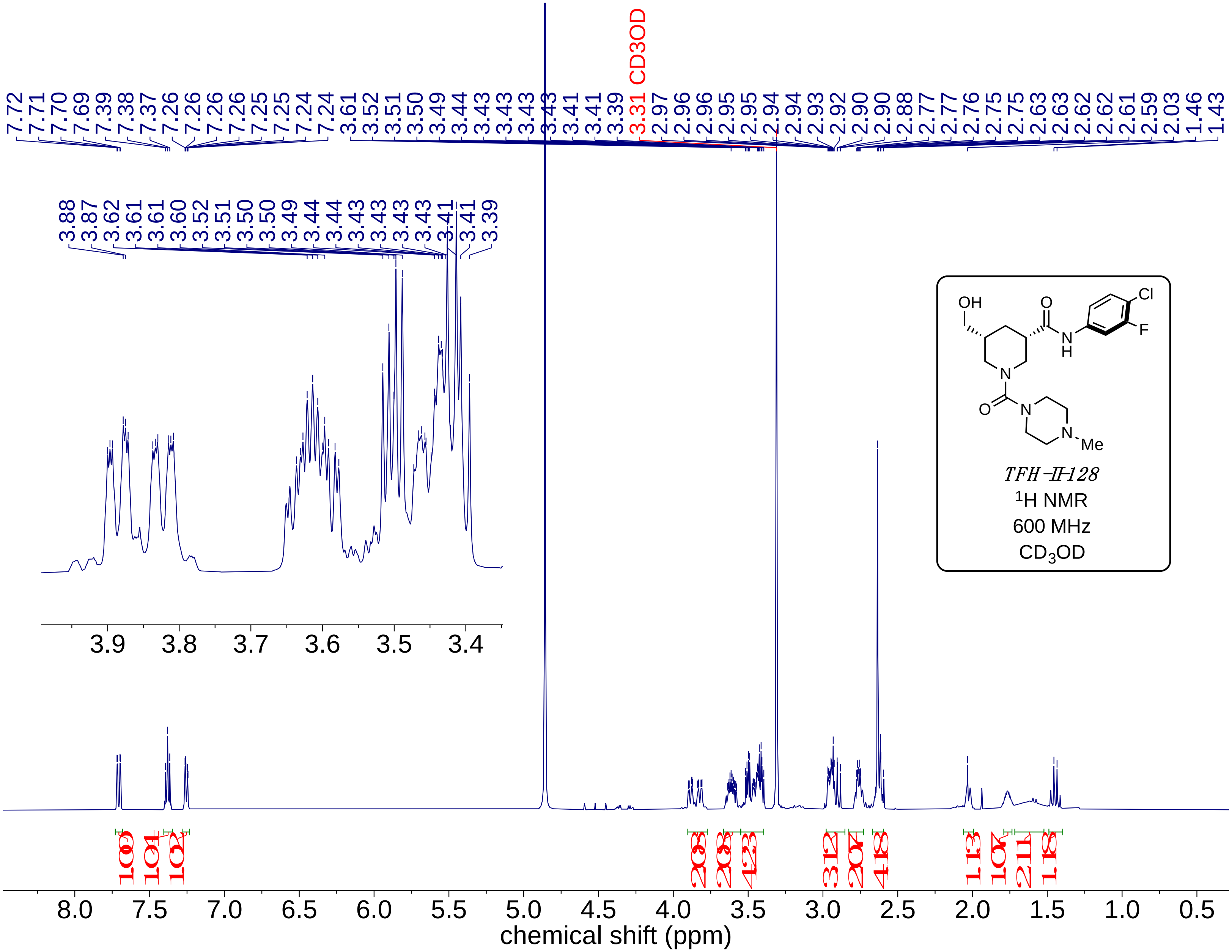

7.72 7.71 7.70 7.69 7.39 7.38 7.37 7.26 7.26 7.26 7.26 7.25 7.25 7.24 7.24 3.61 3.52 3.51 3.50 3.49 3.44 3.43 3.43 3.43 3.43 3.41 3.41 3.39 3.31 2.97 2.96 2.96 2.95 2.95 2.94 2.94 2.93 2.92 2.90 2.90 2.88 2.77 2.77 2.76 2.75 2.75 2.63 2.63 2.62 2.62 2.61 2.59 2.03 1.46 1.43
CD3OD
3.88 3.87 3.62 3.61 3.61 3.60 3.52 3.51 3.50 3.50 3.49 3.44 3.44 3.43 3.43 3.43 3.43 3.41 3.41 3.41 3.39
3.9 3.8 3.7 3.6 3.5 3.4
OH
O
Cl
F
N
H
N
O
N
N
Me
TFH-II-128
^{1}H NMR
600 MHz
CD_3OD
8.0 7.5 7.0 6.5 6.0 5.5 5.0 4.5 4.0 3.5 3.0 2.5 2.0 1.5 1.0 0.5
chemical shift (ppm)

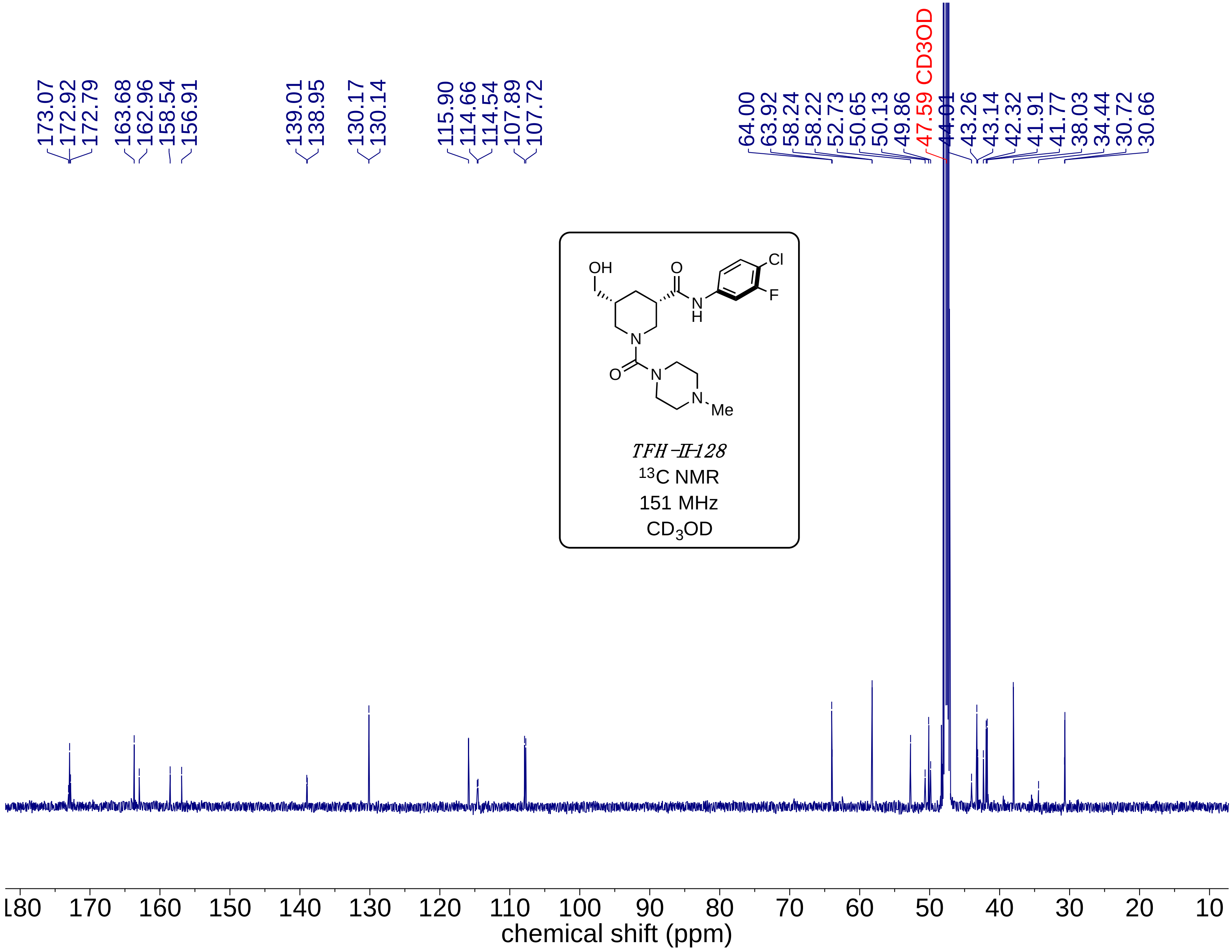
173.07
172.92
172.79
163.68
162.96
158.54
156.91
139.01
138.95
130.17
130.14
115.90
114.66
114.54
107.89
107.72
64.00
63.92
58.24
58.22
52.73
50.65
50.13
49.86
47.59 CD3OD
44.01
43.26
43.14
42.32
41.91
41.77
38.03
34.44
30.72
30.66
OH
O
N
H
Cl
F
N
O
N
N
Me
TFH-II-128
13C NMR
151 MHz
CD3OD
180
170
160
150
140
130
120
110
100
90
80
70
60
50
40
30
20
10
chemical shift (ppm)

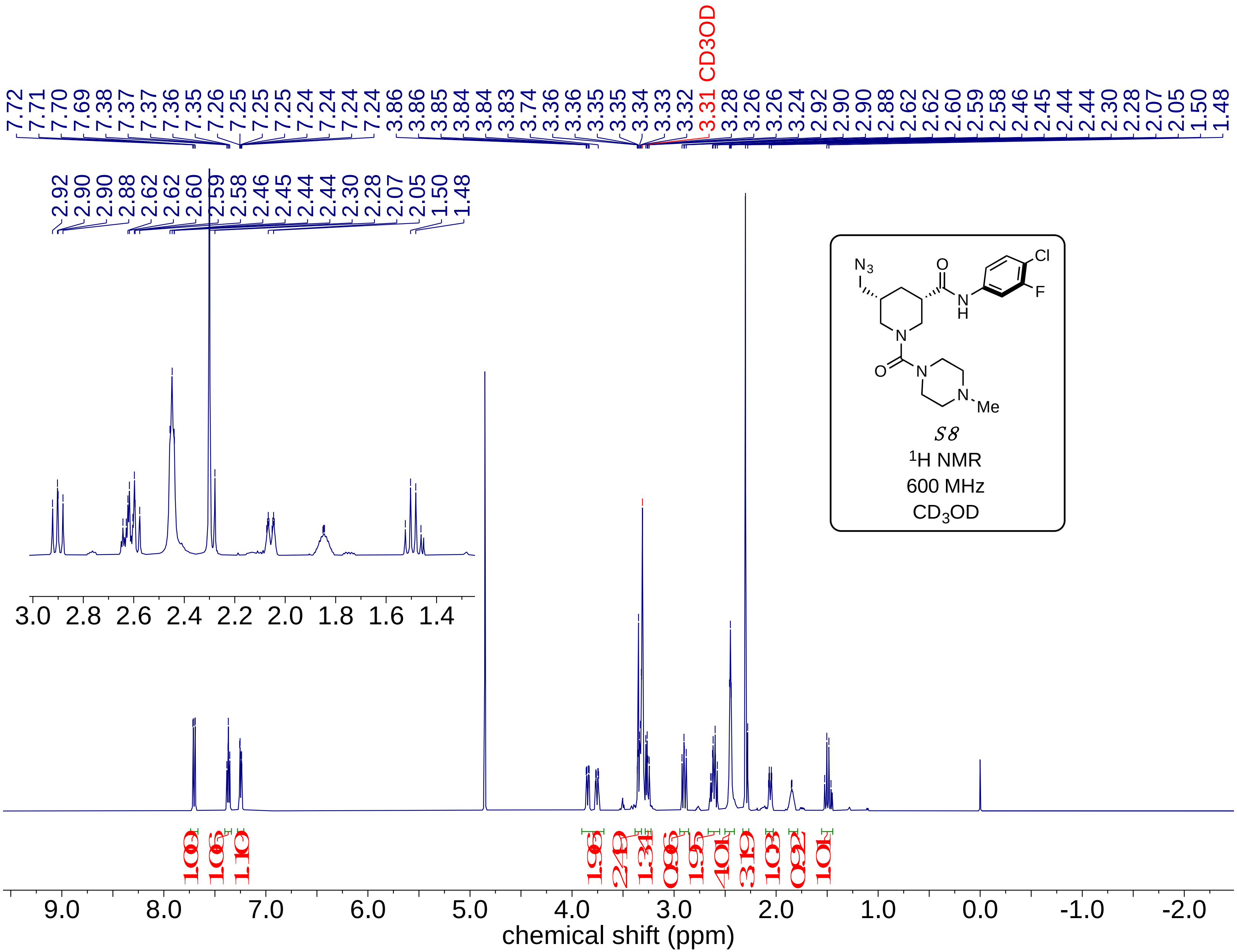

S8
^{1}H NMR
600 MHz
CD_3OD
CD3OD
chemical shift (ppm)

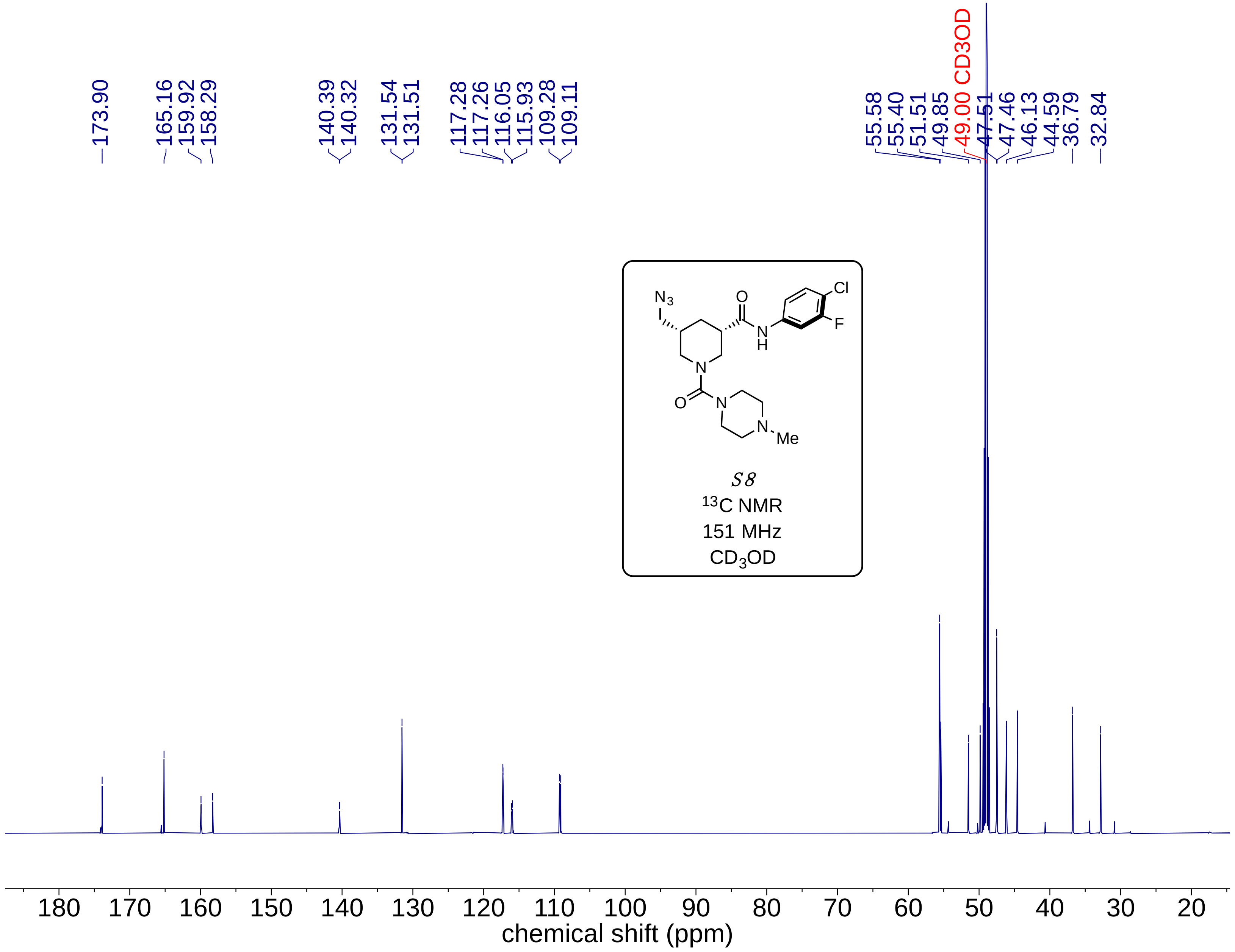

173.90
165.16
159.92
158.29
140.39
140.32
131.54
131.51
117.28
117.26
116.05
115.93
109.28
109.11
55.58
55.40
51.51
49.85
49.00 CD3OD
47.51
47.46
46.13
44.59
36.79
32.84
N3
O
Cl
F
N
H
N
O
N
N
Me
S8
13C NMR
151 MHz
CD3OD
180
170
160
150
140
130
120
110
100
90
80
70
60
50
40
30
20
chemical shift (ppm)

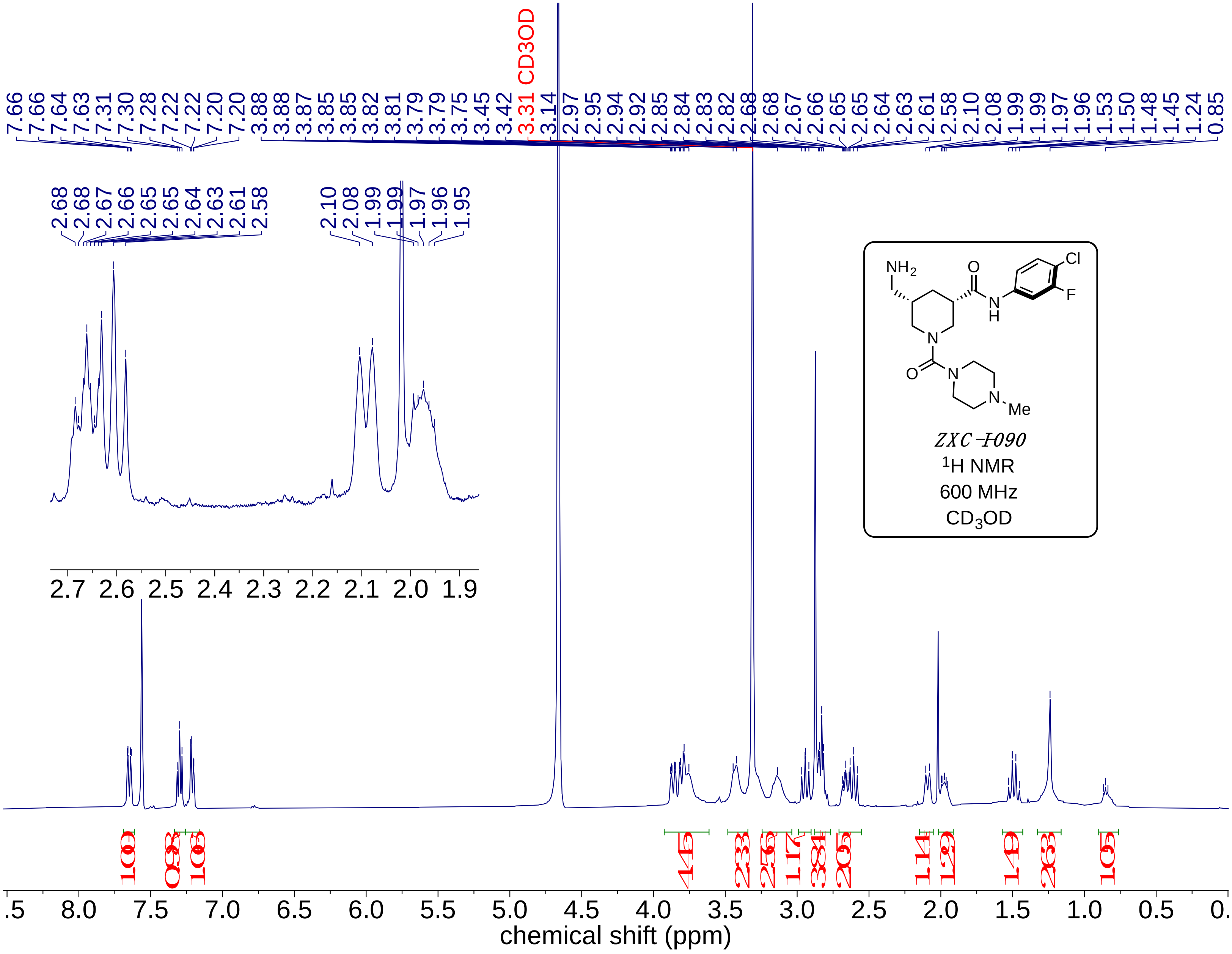
7.66 7.66 7.64 7.63 7.31 7.30 7.28 7.22 7.22 7.20 7.20 3.88 3.88 3.87 3.85 3.85 3.82 3.81 3.79 3.79 3.75 3.45 3.42 3.31 CD3OD 3.14 2.97 2.95 2.94 2.92 2.85 2.84 2.83 2.82 2.68 2.68 2.67 2.66 2.65 2.65 2.64 2.63 2.61 2.58 2.10 2.08 1.99 1.99 1.97 1.96 1.53 1.50 1.48 1.45 1.24 0.85
2.68 2.68 2.67 2.66 2.65 2.65 2.64 2.63 2.61 2.58
2.10 2.08 1.99 1.99 1.97 1.96 1.95
2.7 2.6 2.5 2.4 2.3 2.2 2.1 2.0 1.9
NH2
O
Cl
N
H
F
N
O
N
N
Me
ZXC-I-090
1H NMR
600 MHz
CD3OD
.5 8.0 7.5 7.0 6.5 6.0 5.5 5.0 4.5 4.0 3.5 3.0 2.5 2.0 1.5 1.0 0.5 0.
chemical shift (ppm)

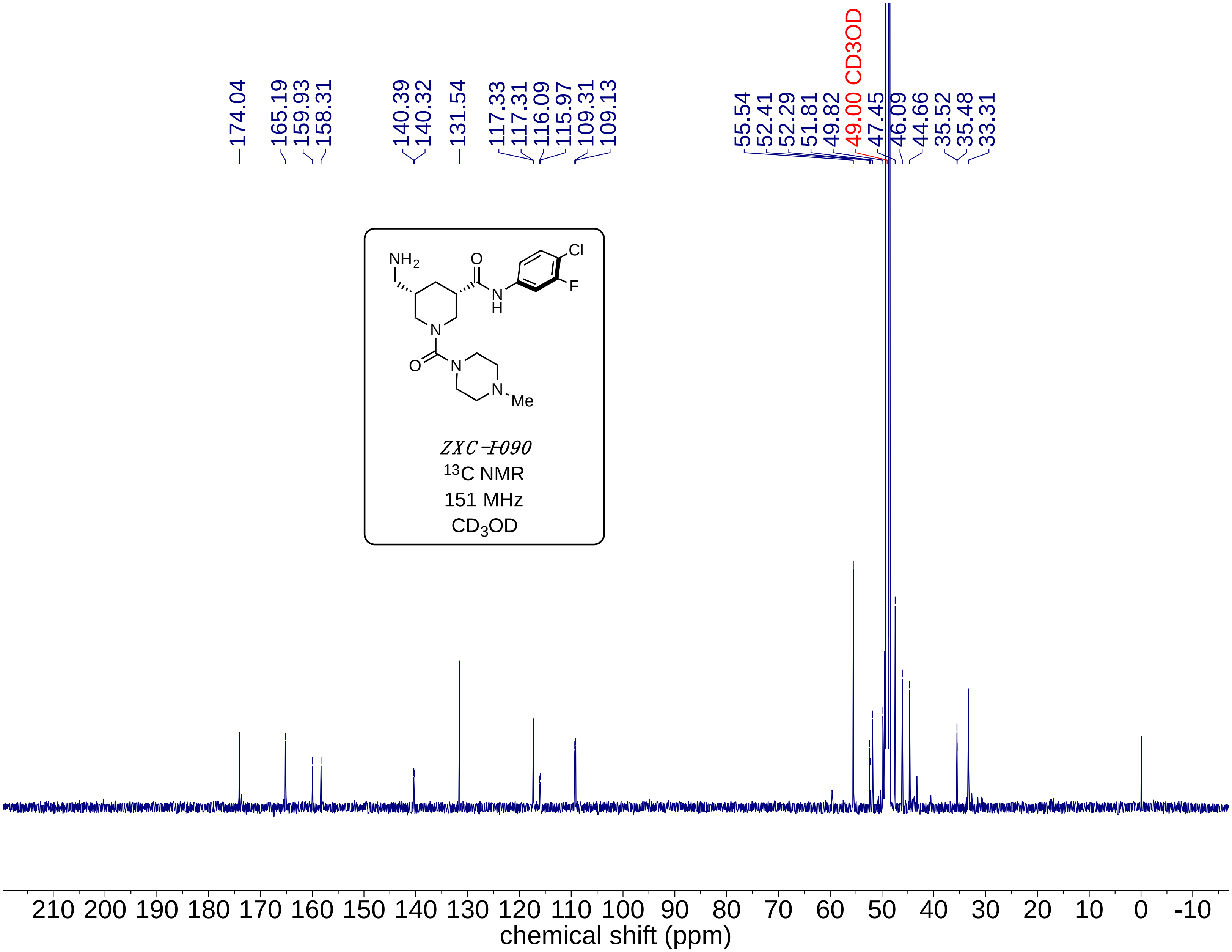

174.04
165.19
159.93
158.31
140.39
140.32
131.54
117.33
117.31
116.09
115.97
109.31
109.13
55.54
52.41
52.29
51.81
49.82
49.00 CD3OD
47.45
46.09
44.66
35.52
35.48
33.31
NH2
O
Cl
N
H
F
N
O
N
N
Me
ZXC-I-090
13C NMR
151 MHz
CD3OD
210 200 190 180 170 160 150 140 130 120 110 100 90 80 70 60 50 40 30 20 10 0 -10
chemical shift (ppm)

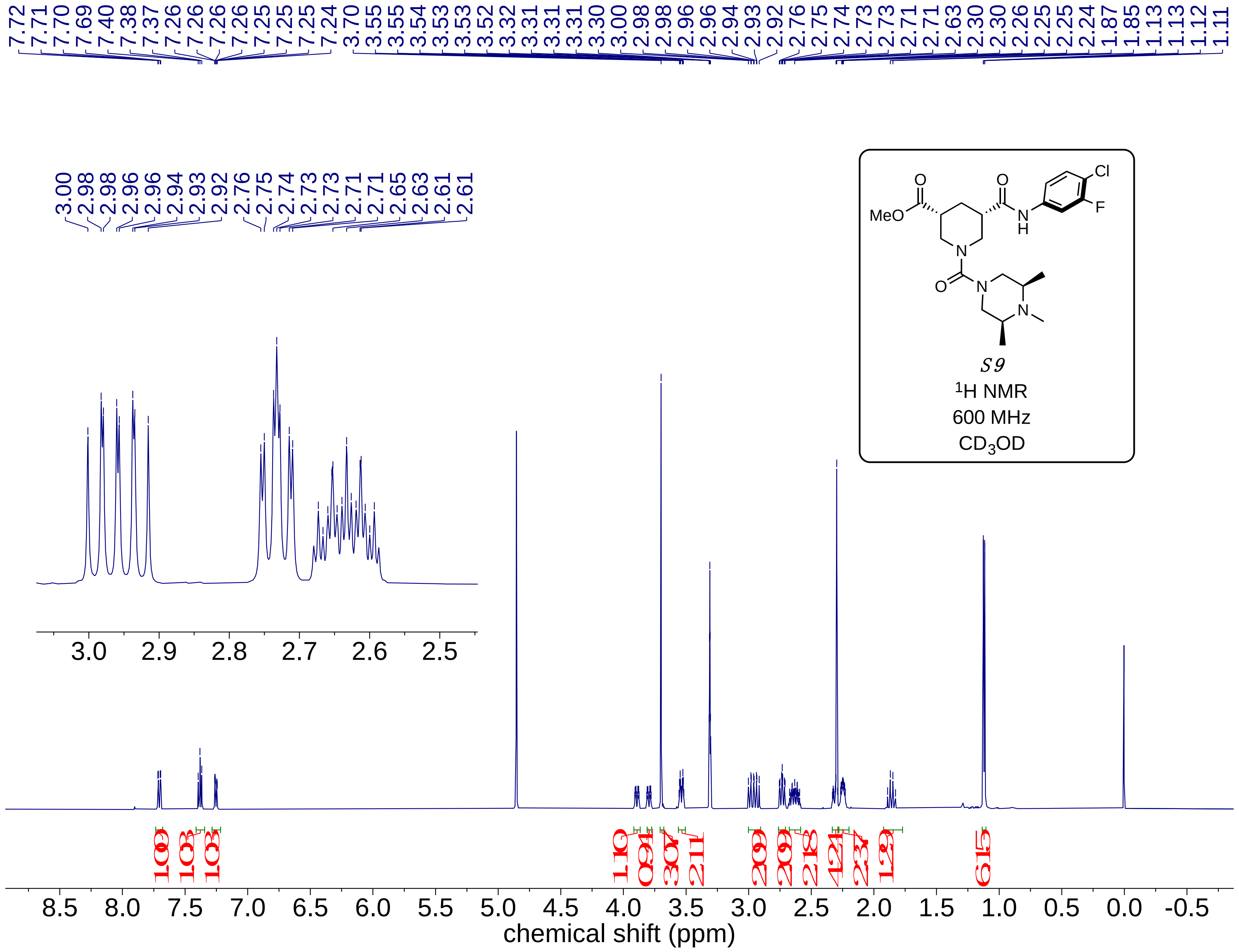

7.72 7.71 7.70 7.69 7.40 7.38 7.37 7.26 7.26 7.26 7.26 7.25 7.25 7.25 7.24 3.70 3.55 3.55 3.54 3.53 3.53 3.52 3.32 3.31 3.31 3.31 3.30 3.00 2.98 2.98 2.96 2.96 2.94 2.93 2.92 2.76 2.75 2.74 2.73 2.73 2.71 2.71 2.63 2.30 2.30 2.26 2.25 2.25 2.24 1.87 1.85 1.13 1.13 1.12 1.11
3.00 2.98 2.98 2.96 2.96 2.94 2.93 2.92 2.76 2.75 2.74 2.73 2.73 2.71 2.71 2.65 2.63 2.61 2.61
3.0 2.9 2.8 2.7 2.6 2.5
MeO
O
N
H
Cl
F
S9
^{1}H NMR
600 MHz
CD_3OD
8.5 8.0 7.5 7.0 6.5 6.0 5.5 5.0 4.5 4.0 3.5 3.0 2.5 2.0 1.5 1.0 0.5 0.0 -0.5
chemical shift (ppm)

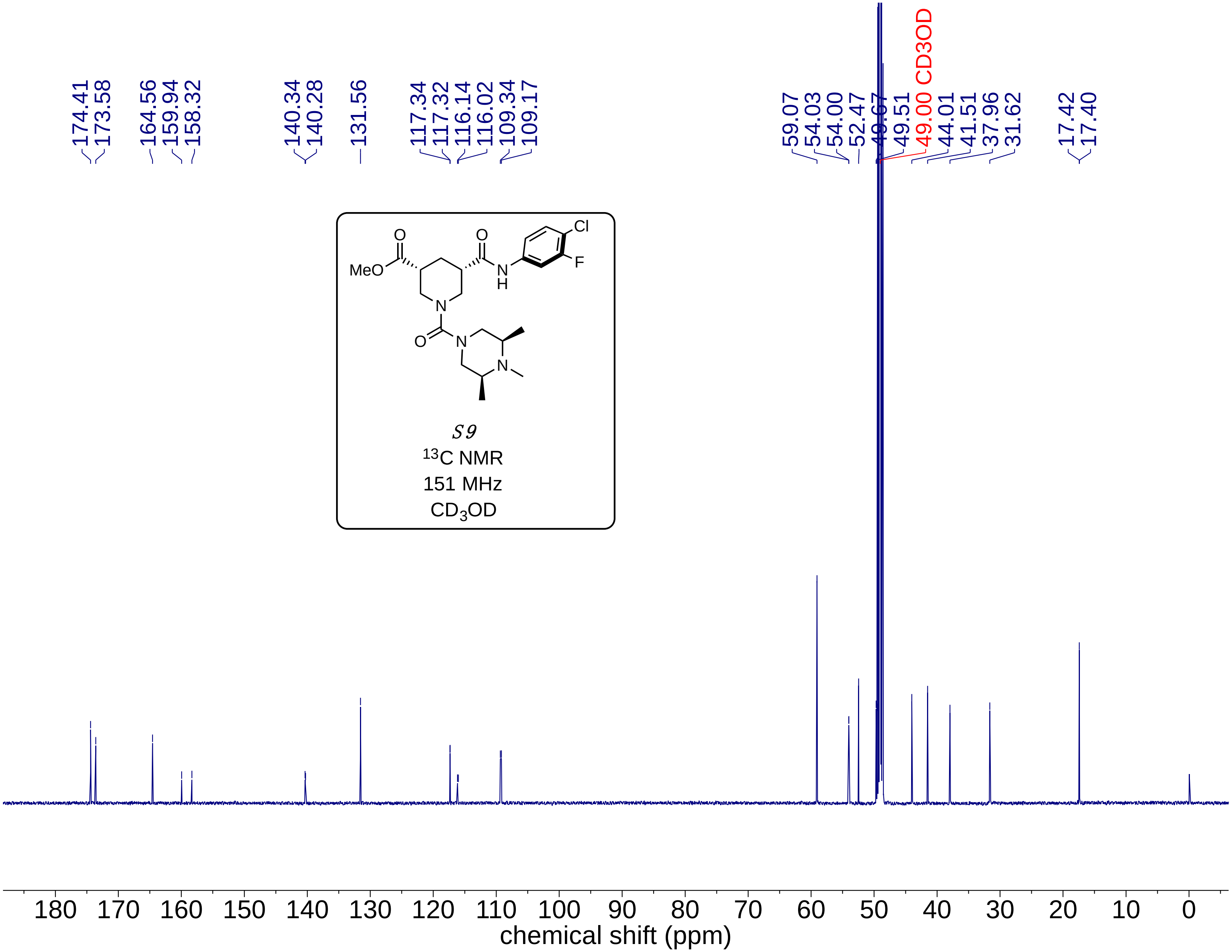
174.41
173.58
164.56
159.94
158.32
140.34
140.28
131.56
117.34
117.32
116.14
116.02
109.34
109.17
59.07
54.03
54.00
52.47
49.67
49.51
49.00 CD3OD
44.01
41.51
37.96
31.62
17.42
17.40
MeO
O
N
H
Cl
F
S9
13C NMR
151 MHz
CD3OD
180 170 160 150 140 130 120 110 100 90 80 70 60 50 40 30 20 10 0
chemical shift (ppm)

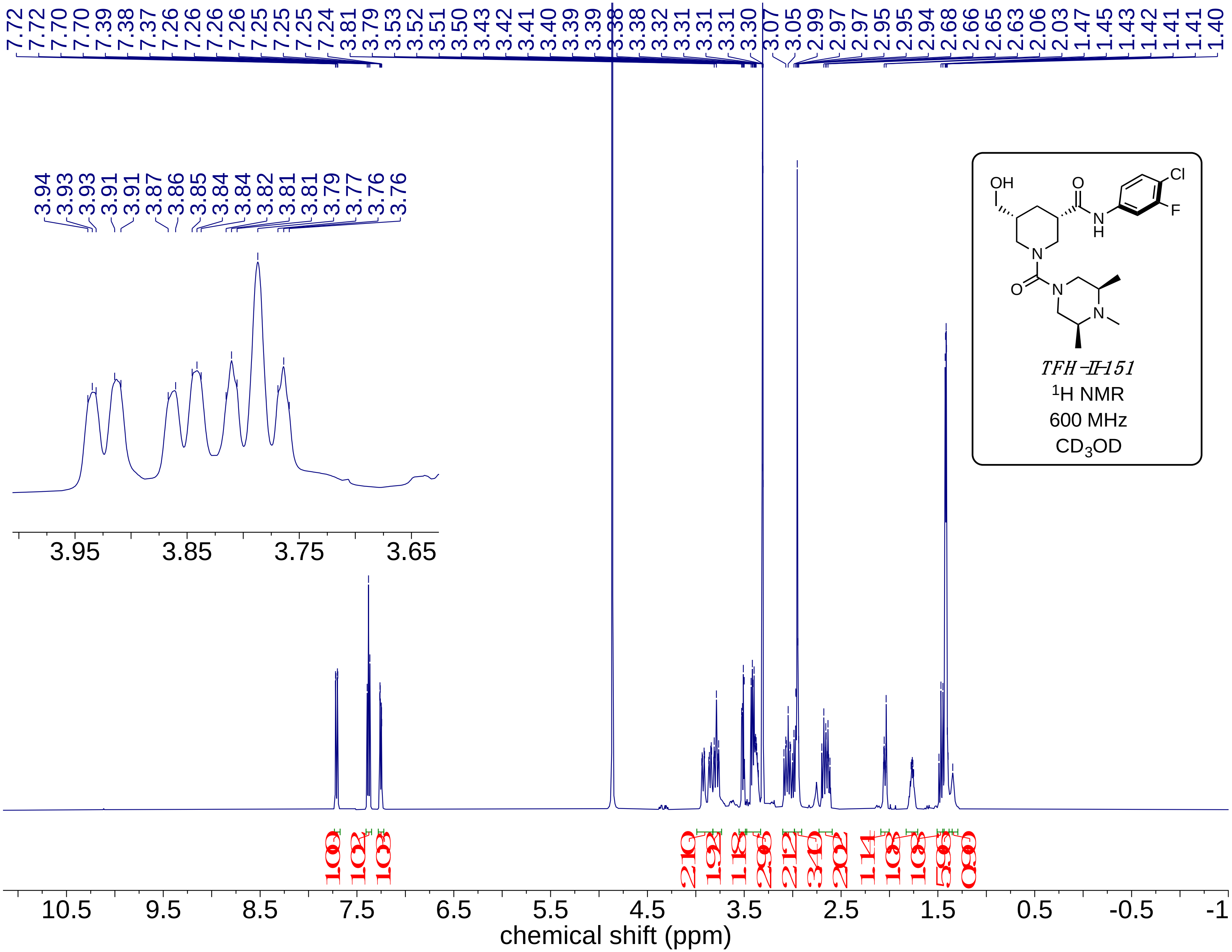

7.72 7.72 7.70 7.70 7.39 7.38 7.37 7.26 7.26 7.26 7.26 7.25 7.25 7.25 7.24 3.81 3.79 3.53 3.52 3.51 3.50 3.43 3.42 3.41 3.40 3.39 3.39 3.38 3.38 3.32 3.31 3.31 3.31 3.30 3.07 3.05 2.99 2.97 2.97 2.95 2.95 2.94 2.68 2.66 2.65 2.63 2.06 2.03 1.47 1.45 1.43 1.42 1.41 1.41 1.40
3.94 3.93 3.93 3.91 3.91 3.87 3.86 3.85 3.84 3.84 3.82 3.81 3.81 3.79 3.77 3.76 3.76
3.95
3.85
3.75
3.65
OH
O
Cl
F
N
H
N
O
N
N
TFH-II-151
^{1}H NMR
600 MHz
CD_3OD
10.5
9.5
8.5
7.5
6.5
5.5
4.5
3.5
2.5
1.5
0.5
-0.5
-1
chemical shift (ppm)

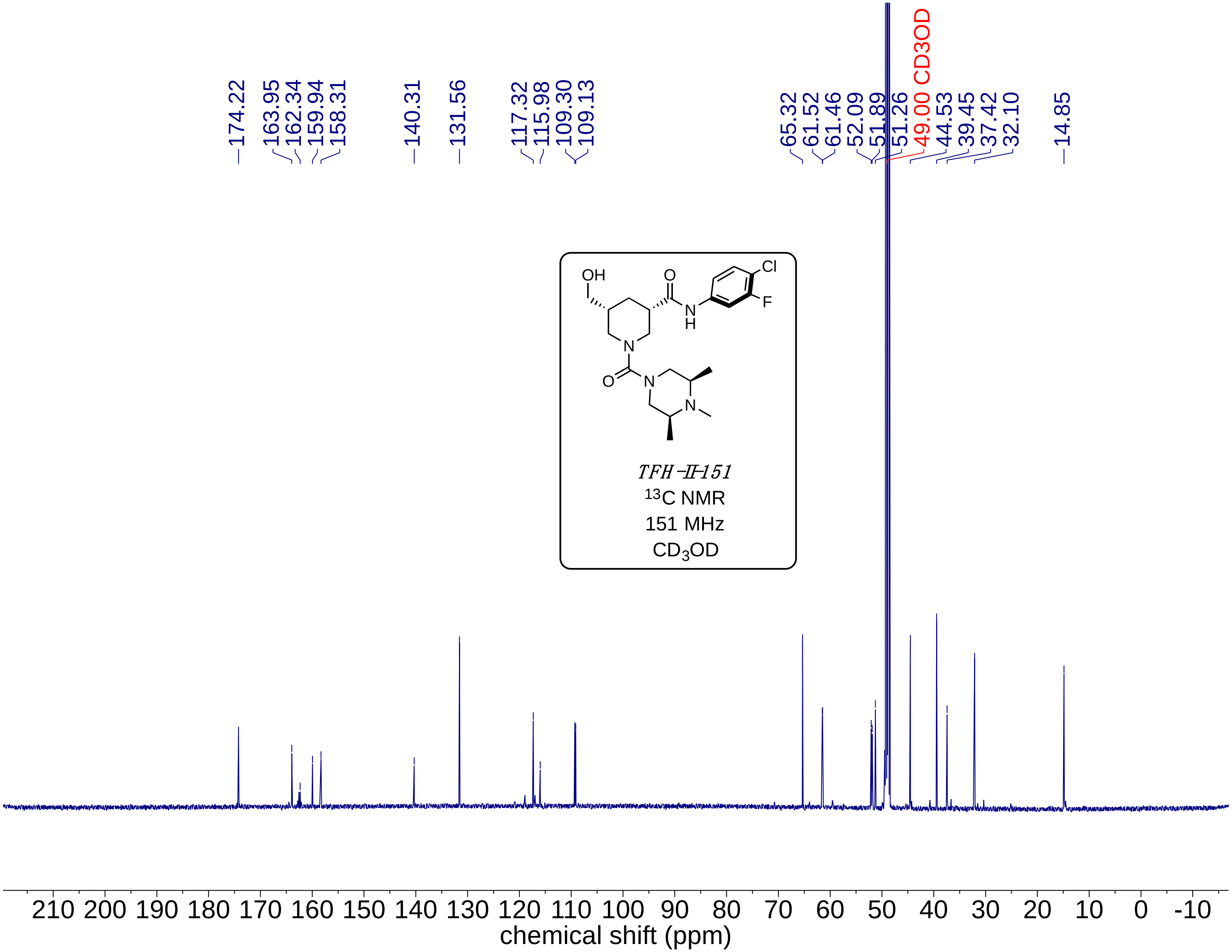

174.22
163.95
162.34
159.94
158.31
140.31
131.56
117.32
115.98
109.30
109.13
65.32
61.52
61.46
52.09
51.89
51.26
49.00 CD3OD
44.53
39.45
37.42
32.10
14.85
OH
O
Cl
F
N
H
N
O
N
N
TFH-II-151
13C NMR
151 MHz
CD3OD
210 200 190 180 170 160 150 140 130 120 110 100 90 80 70 60 50 40 30 20 10 0 -10
chemical shift (ppm)

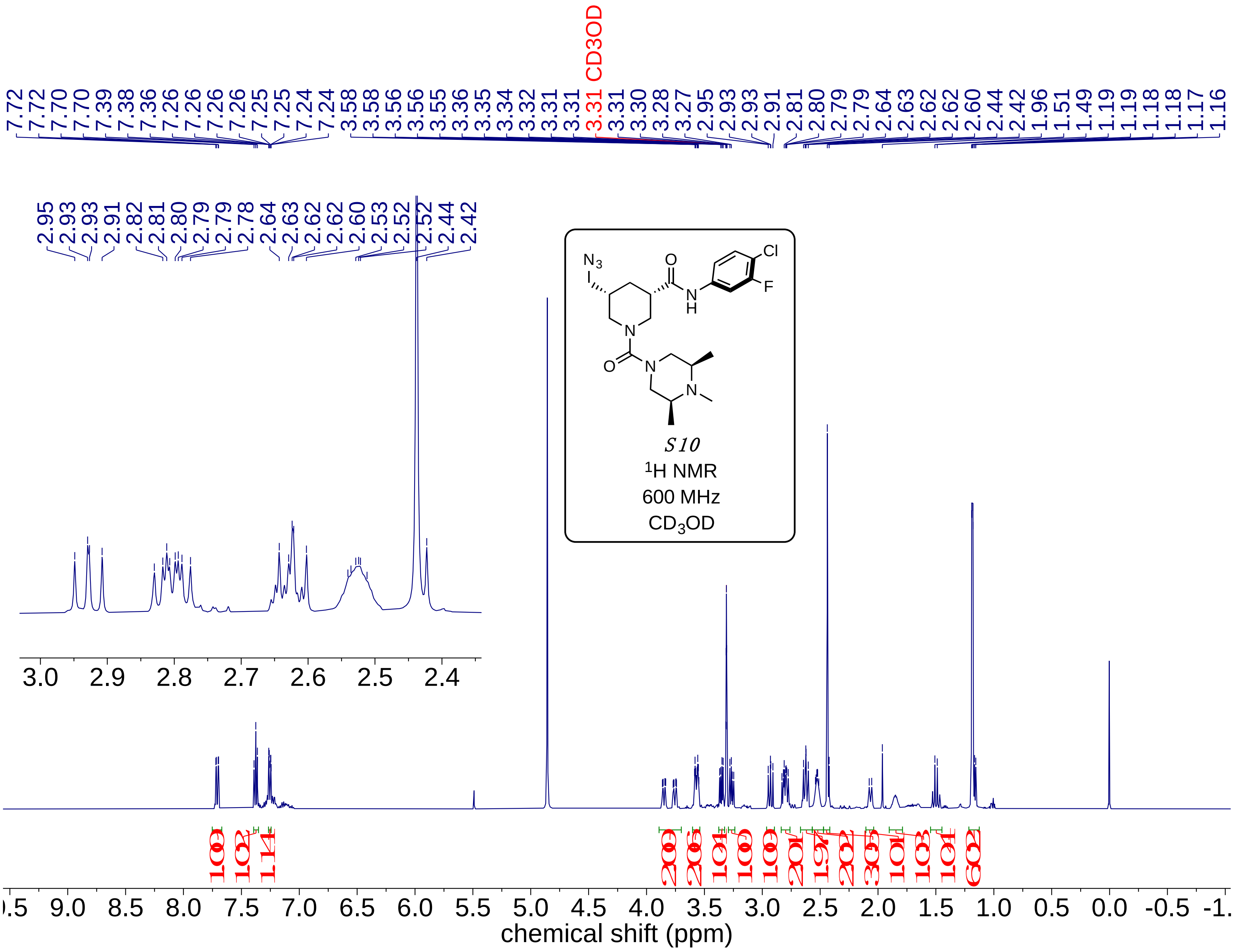
CD3OD
S10
^{1}H NMR
600 MHz
CD_3OD
chemical shift (ppm)

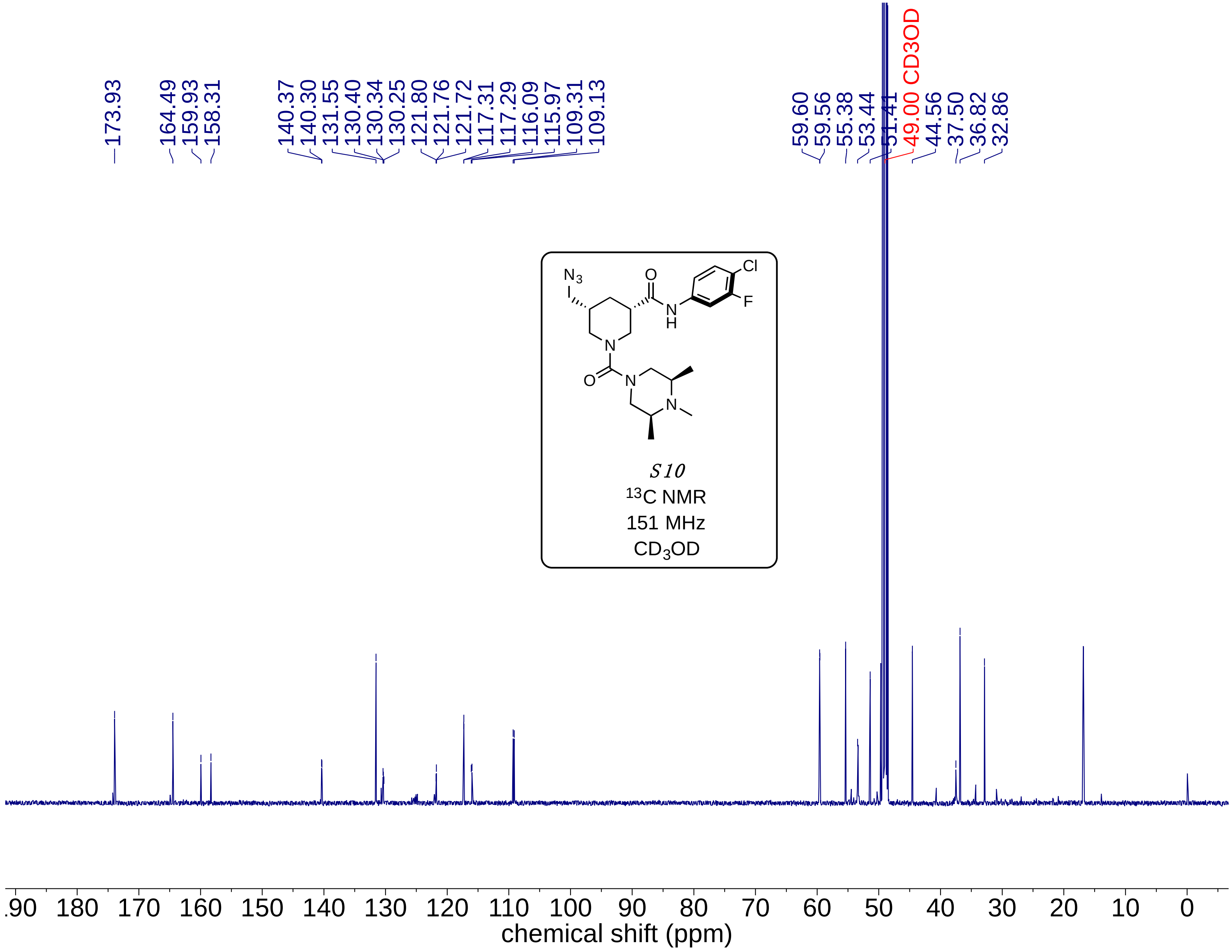

173.93
164.49
159.93
158.31
140.37
140.30
131.55
130.40
130.34
130.25
121.80
121.76
121.72
117.31
117.29
116.09
115.97
109.31
109.13
59.60
59.56
55.38
53.44
51.41
49.00 CD3OD
44.56
37.50
36.82
32.86
N3
O
Cl
F
N
H
N
O
N
N
S10
13C NMR
151 MHz
CD3OD
190 180 170 160 150 140 130 120 110 100 90 80 70 60 50 40 30 20 10 0
chemical shift (ppm)

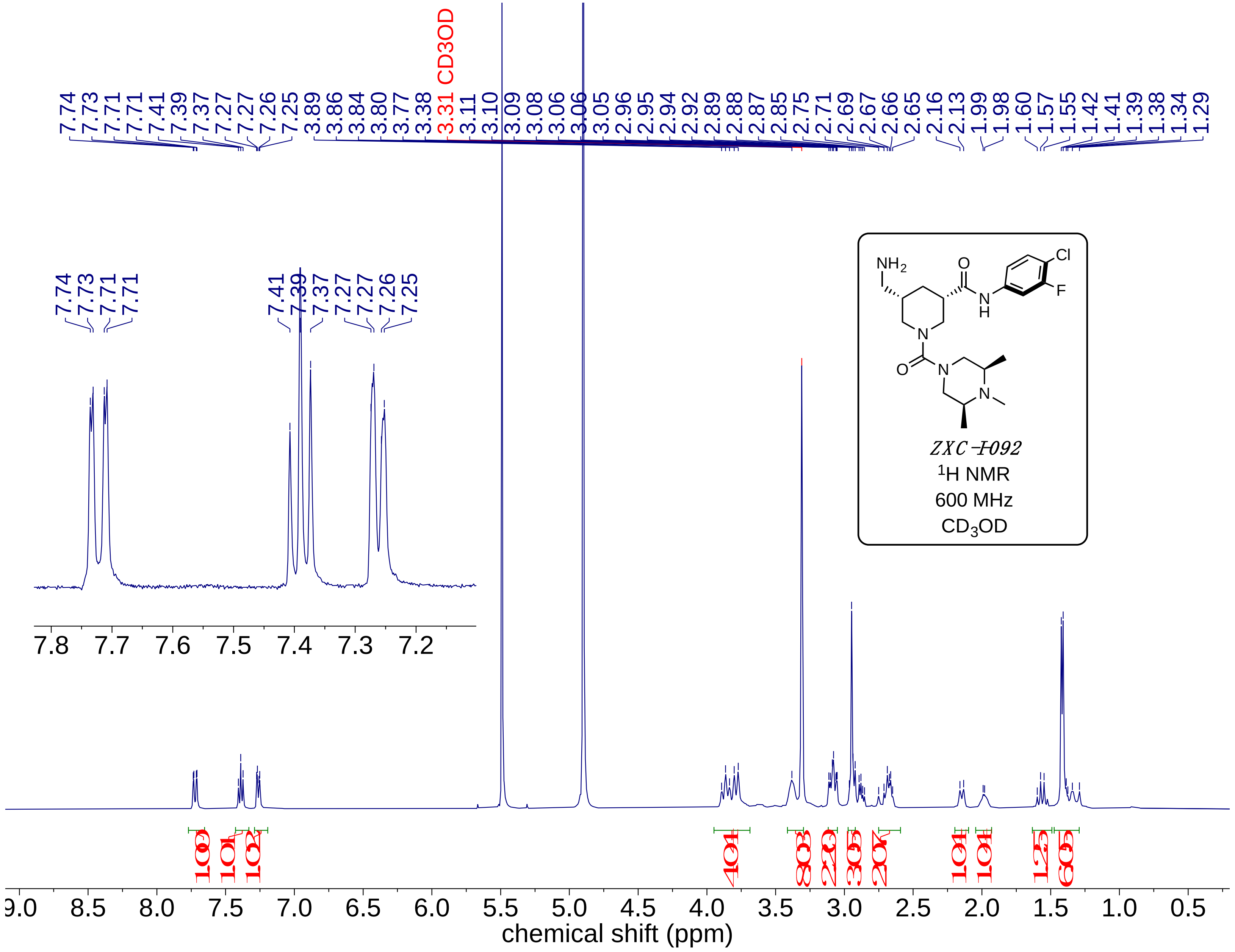
ZXC-I-092
^{1}H NMR
600 MHz
CD_3OD
CD3OD
chemical shift (ppm)

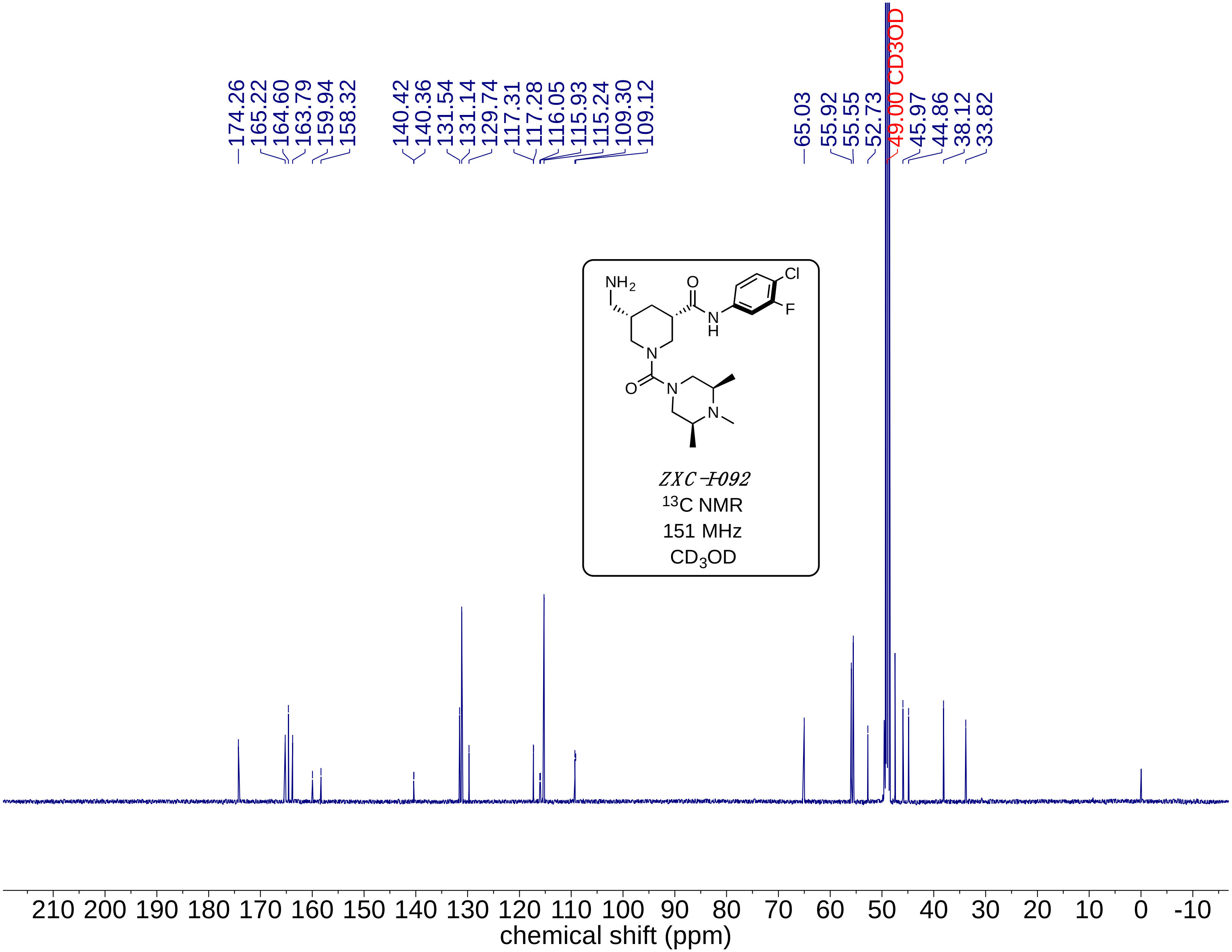

174.26
165.22
164.60
163.79
159.94
158.32
140.42
140.36
131.54
131.14
129.74
117.31
117.28
116.05
115.93
115.24
109.30
109.12
65.03
55.92
55.55
52.73
49.00 CD3OD
45.97
44.86
38.12
33.82
NH2
O
Cl
F
N
H
N
O
N
N
ZXC-I-092
13C NMR
151 MHz
CD3OD
210 200 190 180 170 160 150 140 130 120 110 100 90 80 70 60 50 40 30 20 10 0 -10
chemical shift (ppm)

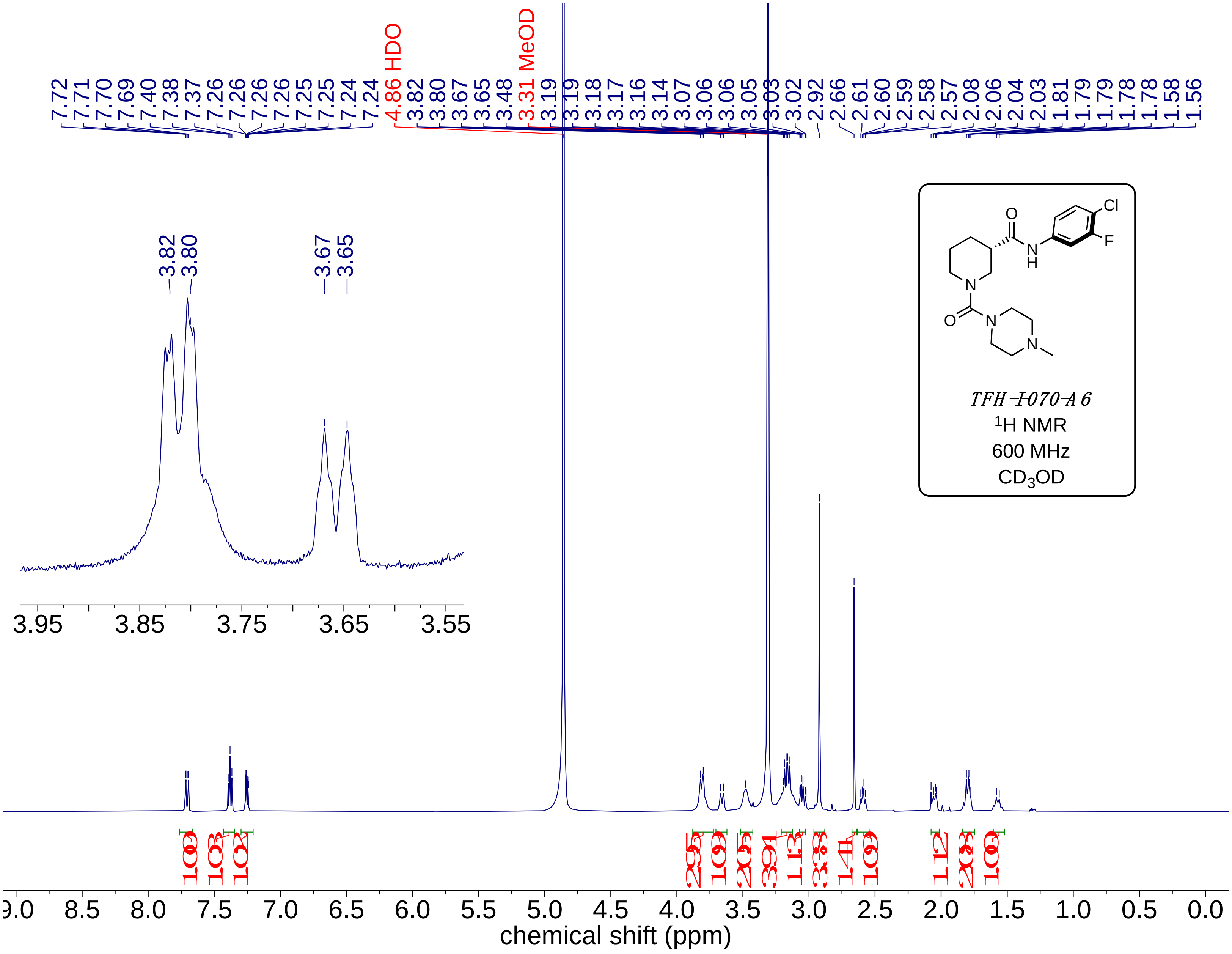

7.72 7.71 7.70 7.69 7.40 7.38 7.37 7.26 7.26 7.26 7.26 7.25 7.25 7.24 7.24
4.86 HDO
3.82 3.80 3.67 3.65 3.48
3.31 MeOD
3.19 3.19 3.18 3.17 3.16 3.14 3.07 3.06 3.06 3.05 3.03 3.02 2.92 2.66 2.61 2.60 2.59 2.58 2.57 2.08 2.06 2.04 2.03 1.81 1.79 1.79 1.78 1.78 1.58 1.56
3.82 3.80 3.67 3.65
3.95 3.85 3.75 3.65 3.55
TFH-I-070-A6
^{1}H NMR
600 MHz
CD_3OD
9.0 8.5 8.0 7.5 7.0 6.5 6.0 5.5 5.0 4.5 4.0 3.5 3.0 2.5 2.0 1.5 1.0 0.5 0.0
chemical shift (ppm)

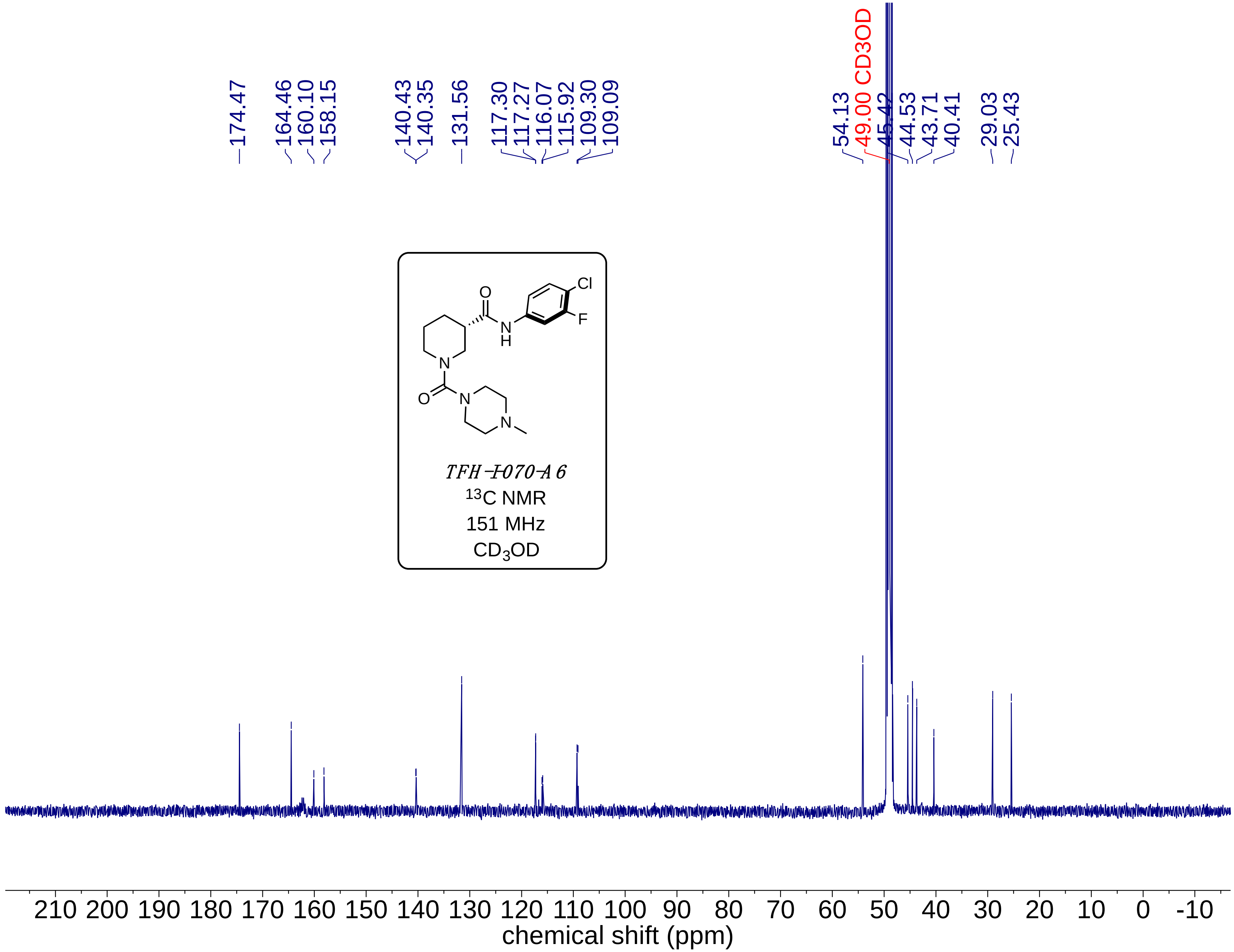
174.47
164.46
160.10
158.15
140.43
140.35
131.56
117.30
117.27
116.07
115.92
109.30
109.09
54.13
49.00 CD3OD
45.42
44.53
43.71
40.41
29.03
25.43
TFH-I-070-A 6
13C NMR
151 MHz
CD3OD
210 200 190 180 170 160 150 140 130 120 110 100 90 80 70 60 50 40 30 20 10 0 -10
chemical shift (ppm)

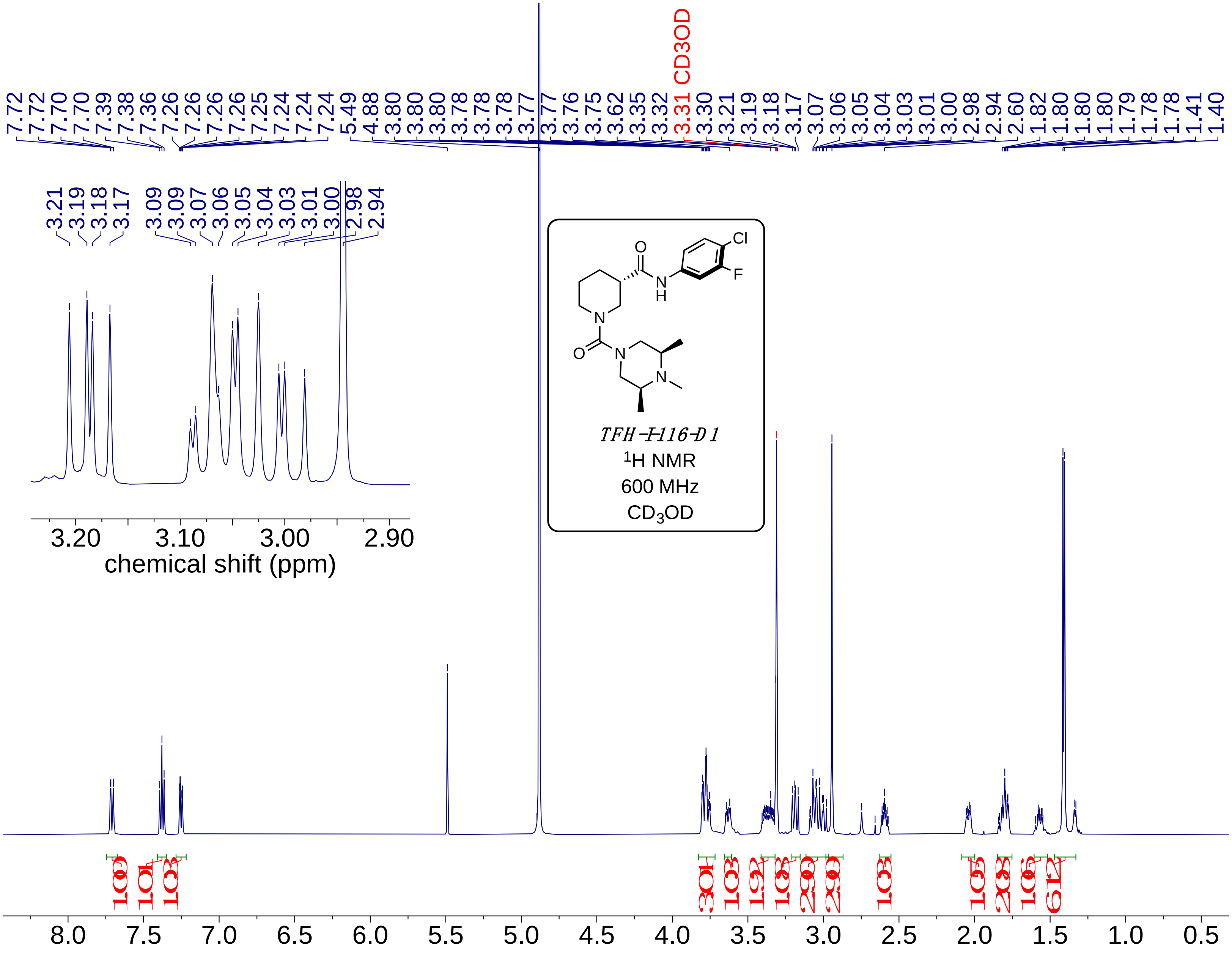
7.72 7.72 7.70 7.70 7.39 7.38 7.36 7.26 7.26 7.26 7.26 7.25 7.24 7.24 7.24 5.49 4.88 3.80 3.80 3.80 3.78 3.78 3.78 3.77 3.77 3.76 3.75 3.62 3.35 3.32 3.31 CD3OD 3.30 3.21 3.19 3.18 3.17 3.07 3.06 3.05 3.04 3.03 3.01 3.00 2.98 2.94 2.60 1.82 1.80 1.80 1.80 1.79 1.78 1.78 1.41 1.40
3.21 3.19 3.18 3.17 3.09 3.09 3.07 3.06 3.05 3.04 3.03 3.01 3.00 2.98 2.94
3.20 3.10 3.00 2.90
chemical shift (ppm)
O
Cl
N
H
F
N
O
N
N
TFH-H116-D1
^{1}H NMR
600 MHz
CD_3OD
8.0 7.5 7.0 6.5 6.0 5.5 5.0 4.5 4.0 3.5 3.0 2.5 2.0 1.5 1.0 0.5

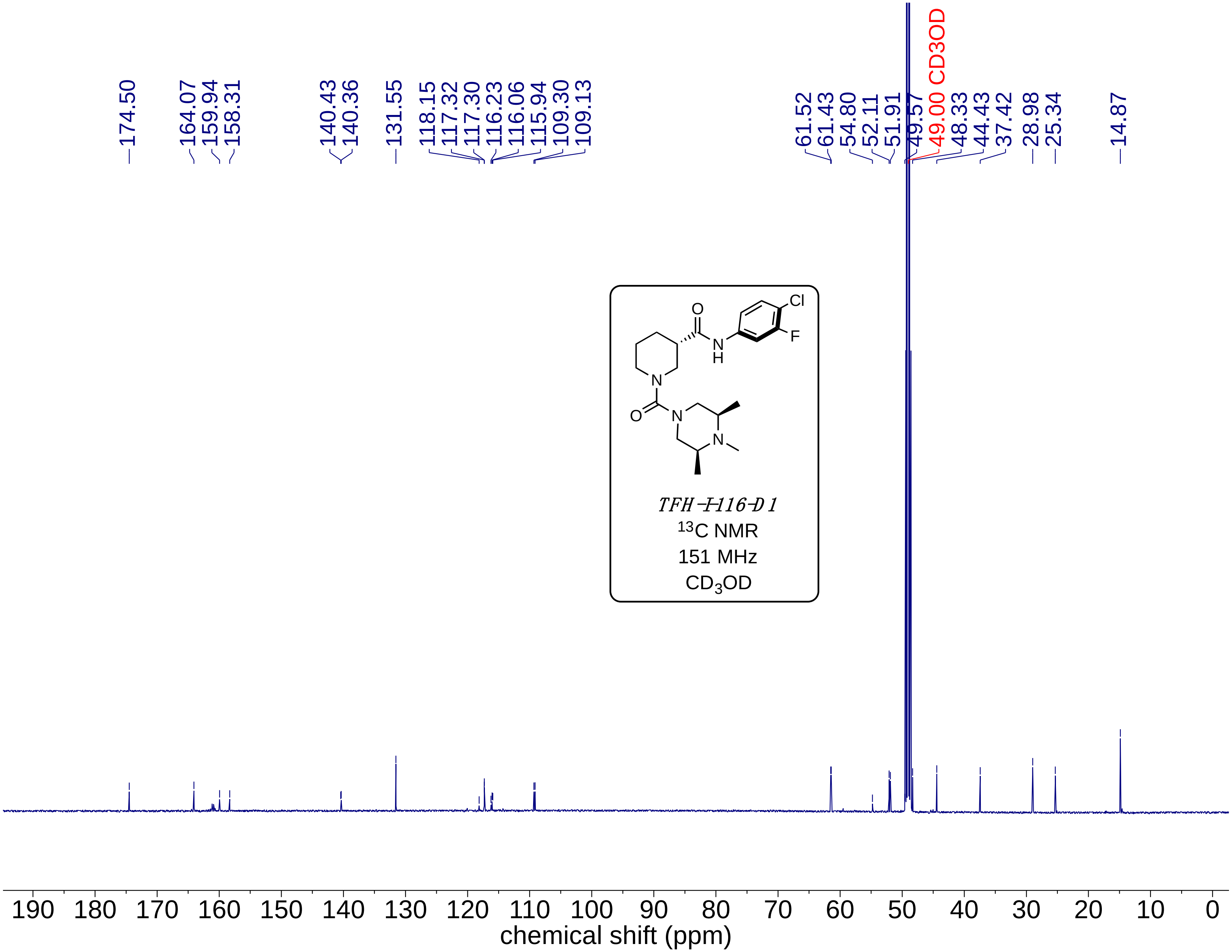
174.50
164.07
159.94
158.31
140.43
140.36
131.55
118.15
117.32
117.30
116.23
116.06
115.94
109.30
109.13
61.52
61.43
54.80
52.11
51.91
49.57
49.00 CD3OD
48.33
44.43
37.42
28.98
25.34
14.87
O
Cl
N
H
F
N
O
N
N
TFH-I-116-D1
^{13}C NMR
151 MHz
CD_3OD
190 180 170 160 150 140 130 120 110 100 90 80 70 60 50 40 30 20 10 0
chemical shift (ppm)
